# ZIKV Phylogenetic Characterization Reveals Evolutionary Diversity, Regional Dissemination, and Emergence of African Lineages in Brazil

**DOI:** 10.1002/jobm.70122

**Published:** 2025-10-28

**Authors:** Thayane da Encarnação Sá‐Guimarães, Pedro Panzenhagen, Anita Ferreira do Valle, Carlos Adam Conte Junior, Mônica Ferreira Moreira

**Affiliations:** ^1^ Instituto de Química, Departamento de Bioquímica, Laboratório de Bioquímica e Biologia Molecular de Vetores Universidade Federal do Rio de Janeiro Rio de Janeiro Brasil; ^2^ Instituto de Química, Departamento de Bioquímica, Programa de Pós‐Graduação em Bioquímica Universidade Federal do Rio de Janeiro Rio de Janeiro Brasil; ^3^ Instituto de Química, Departamento de Bioquímica, Laboratório de Estudos Aplicados em Fotossíntese Universidade Federal do Rio de Janeiro Rio de Janeiro Brasil; ^4^ Instituto de Química, Departamento de Bioquímica, Núcleo de Análises de Alimentos, Laboratório de Apoio ao Desenvolvimento Tecnológico Universidade Federal do Rio de Janeiro Rio de Janeiro Brasil; ^5^ Instituto de Química, Departamento de Bioquímica, Laboratório de Análises Avançadas em Bioquímica e Biologia Molecular Universidade Federal do Rio de Janeiro Rio de Janeiro Brasil; ^6^ Instituto Nacional de Ciência e Tecnologia em Entomologia Molecular ‐ INCT‐EM Universidade Federal do Rio de Janeiro Rio de Janeiro Brasil

**Keywords:** epidemiological surveillance, polymorphism, Zika virus

## Abstract

During the last epidemic in 2015–2016, Zika virus (ZIKV) gaining worldwide attention due to its association with neurological complications such as microcephaly and Guillain‐Barré syndrome. ZIKV can be transmitted through vector‐borne, sexual, maternal‐fetal, and blood transfusion transmission. This study aimed to investigate the shedding pattern of ZIKV using genomic data deposited in NCBI and compare clinical and mosquito samples to explore transmission patterns. A total of 1889 genomic deposits were analyzed, with the majority originating from the Americas (59%) and Asia (27%), where the Asian lineage predominates. In Africa, the West African lineage is more prevalent. Interestingly, field‐collected mosquito eggs samples, tested positive for the East African ZIKV lineage, suggesting the possibility of mosquito eggs serving as viral reservoirs. Sequencing of the C‐prM fragment from both clinical and mosquito samples enabled the construction of a phylogenetic tree with sequences from NCBI. Revealing a significant divergence, with samples clustering with African lineages, particularly the East African lineage, indicating a potential introduction of these lineages into regions outside Africa. This study emphasizes the value of genomic data to uncover the origins, transmission patterns, and evolutionary dynamics of ZIKV, providing key insights into the lineage diversity and epidemiological significance of this arbovirus.

**Abbreviations**
BLASTn: Basic Local Alignment Search Tool (nucleotide version)
CAUAP–UFRJ: Ethics Committee on Animal Use in Research – Federal University of Rio de Janeiro
cDNA: complementary deoxyribonucleic acid
C–prM: genomic region encoding the capsid (C) and precursor membrane (prM) proteins
E: envelope protein
GFP: green fluorescent protein.
HUCFF/FM/UFRJ: Clementino Fraga Filho University Hospital/School of Medicine/Federal University of Rio de Janeiro
LB: Luria–Bertani broth/agar
M: membrane protein
MAFFT: Multiple Alignment using Fast Fourier Transform
NCBI: National Center for Biotechnology Information
NS: nonstructural protein
NS1, NS3, NS5: nonstructural proteins 1, 3, and 5
PCR: polymerase chain reaction
prM: precursor membrane protein
qPCR: quantitative polymerase chain reaction
RPM: revolutions per minute
SNP: single‐nucleotide polymorphism
WGS: whole genome sequencing
ZIKV: Zika virus

## 1 Introduction

The Zika virus (ZIKV) was first identified in Africa during research funded by the Rockefeller Foundation that aimed to investigate the enzootic or sylvatic cycle of the yellow fever virus and identify other arboviruses. Then the first description of ZIKV occurred in 1947 with the virus isolation from samples of sentinel monkey blood called Rhesus 766, which comes from the Uganda Zika Forest [1]. In 1954, ZIKV was described as a human pathogen after isolation from a human sample [2]. Sparse cases of Zika fever were reported in Africa and Asia until the first outbreak on Yap Island (Federated States of Micronesia) in 2007 [3]. The second outbreak was reported in 2013 and 2014 in French Polynesia [4], linked to Guillain‐Barré Syndrome in adults and subsequent association with microcephaly in neonates. In 2015 and 2016, in the last outbreak reported, the highest incidence of cases was in the Americas, although it also reached countries in Africa, Asia, and Oceania [5, 6, 7].

ZIKV transmission mainly occurs via vector route through mosquito bites, predominantly from the *Aedes* genus, with *Aedes aegypti* as the main transmitting species described in the Americas [8]. Virus transmission can also occur through non‐vector routes such as blood transfusion, sexually transmitted infection (oral, anal or vaginal) and vertical transmission (mother to fetus) [7]. The diagnosis is made by laboratory tests through serological and molecular methods. Molecular methods are more effective in avoiding cross‐reactions, frequent in serological methods, when other arboviruses co‐circulation present similar symptoms, such as dengue and chikungunya [7, 9].

The C‐prM region of the ZIKV is vital in the viral lifecycle, influencing assembly, maturation, and infectivity. This region encodes two structural proteins: the capsid (C) protein, which encapsulates the viral RNA, and the precursor membrane (prM) protein, which undergoes cleavage during maturation to form the membrane (M) protein [10]. It is well known that mutations within the C‐prM region can significantly affect viral assembly and pathogenicity. For instance, prM has been shown to influence virion stability and infectivity, with specific mutations altering its ability to modulate the viral lifecycle and induce cytopathic effects in host cells [10, 11]. Furthermore, mutations in this region have been associated with the increased neurovirulence and transmissibility observed in epidemic ZIKV strains, distinguishing them from historical strains [12]. Research highlights that prM cleavage products, such as the pr fragment, can trigger apoptotic pathways in host cells, contributing to the observed cytopathology in neural tissues [10]. Moreover, the C‐prM region's interaction with the envelope (E) protein is essential for virion assembly and entry into host cells, highlighting its diversified [13]. These characteristics make the C‐prM region relevant for understanding ZIKV evolution, pathogenesis, and potential therapeutic interventions.

Phylogenetic studies of circulating ZIKV strains have identified three major evolutionary lineages—East African (prototype group MR766), West African (Nigerian group), and Asian (Micronesia and Malaysia)—each evolving separately based on their geographic origins [14, 15, 16]. Among these, the Asian lineage has been predominantly associated with major ZIKV outbreaks of the 21st century, beginning with the 2007 outbreak in Micronesia, followed by the 2013 epidemic in French Polynesia, and the large 2015 outbreak in the Americas, including Brazil [15]. Although the Asian lineage is commonly identified as the main driver of these global epidemics, recent evidence challenges this narrative. In this context, two studies have identified East African lineage ZIKV strains in wild animals and mosquitoes in Brazilian states, suggesting a previously unrecognized presence of African lineages during the 2015–2016 epidemic in Brazil [17, 18]. This poses new questions about the simultaneous circulation of African lineages and their potential role in shaping the epidemiological and genetic landscape of ZIKV outbreaks. Investigating these findings is key to understanding the full scope of ZIKV evolution and its implications for disease transmission, pathogenesis, and public health interventions.

Considering the scientific literature, the molecular analysis of the C‐prM region in ZIKV sequences deposited in the NCBI, in addition to the new sequences provided by this study, brought valuable data on the dispersal patterns of ZIKV lineages, highlighting novel insights that can improve ZIKV epidemiological surveillance efforts.

## 2 Materials and Methods

### 2.1 ZIKV Sequence Prospection and Download

Using the search term “Zika virus,” a sequence database was constructed with all sequences found in the NCBI Genbank platform (https://www.ncbi.nlm.nih.gov/nuccore) from 1947 to October 2022. These sequences were analyzed based on collected information: Accession Number, Country of Isolate, Year of Isolate, Lineage, Host, whether the genome was Complete or Partial (Table S1). To ensure comprehensive analysis, missing parameters from NCBI were supplemented using the Virus Pathogen Resource (https://legacy.viprbrc.org/brc/home.spg?decorator=vipr) and the Bacterial and Viral Bioinformatics Resource Center (https://www.bv-brc.org/). This expanded dataset allowed for a more representative exploration of ZIKV genomic distribution and lineage characteristics.

From the 5523 sequences found in the NCBI, those with metadata lacking “country of origin” were automatically discarded, as well as sequences missing two or more parameters. Hence, 1889 sequences were analyzed, incorporating correlations between Country of Isolate, Year of Isolate, Lineage (East African, West African, or Asian), and Host (human, mosquito, or laboratory). This information allowed for a comprehensive evaluation of the worldwide distribution of ZIKV lineages and the unique characteristics of the virus in each region.

### 2.2 Sample Collection

Clinical samples of saliva, serum, and urine were collected from humans of both sexes, aged between 15 and 40 years, with and without Zika fever symptoms, during the ZIKV outbreak in the state of Rio de Janeiro from 2015 to 2017. This collection followed the protocols established by the committee of ethics in human experimentation (Ethics Protocol: 80709 HUCFF/FM/UFRJ) and were stored in 50 mL Falcon tubes (Corning) at −20°C for further molecular analyses.

Mosquitoes and *A. aegypti* vector eggs were collected from the field in the state of Rio de Janeiro, Brazil, using ozMozEco® traps for analysis and comparison with the profile observed in the selected ZIKV sequences. The genomic material of these insects was extracted and used for molecular analyses and sequencing. The eggs were divided into two groups: one for genomic material extraction and the other for incubation to allow hatching and subsequent analysis to verify the viability of the collected eggs.

*A. aegypti* mosquito eggs collected in the state of Rio de Janeiro were reared and maintained at a temperature of 28°C with 85% humidity (±5%) in the Department of Biochemistry, IQ, UFRJ, under a 12/12‐h light/dark photoperiod (Ethics Protocol CAUAP‐UFRJ under registration #IBqM011). The larvae and pupae were fed ad libitum with dog food and kept in containers with water.

### 2.3 RNA Extraction and cDNA Synthesis

Total RNA was extracted from 200 μL of supernatant obtained from clinical samples (saliva, serum, and urine) and viruses. Mosquitoes were grouped into pools of five insects, while eggs were grouped into pools of 30. RNA extraction was performed using the TRIzol method (Invitrogen, Carlsbad, CA, USA) following the manufacturer's instructions. The RNA was eluted in 25 μL of nuclease‐free water (GE Healthcare ‐ Hyclone, Utah, USA), and its concentration was estimated using a UV‐Vis Nanodrop™ spectrophotometer (Thermo Scientific) at a wavelength of 260 nm.

DNase treatment was performed with 8 μL of each RNA sample, 1 μL of 10x DNase I buffer, and 1 U RNase‐free DNase (Fermentas, Burling, Canada), following the manufacturer's instructions. The RNA was incubated with DNase for 30 min at 37°C, and the enzyme action was stopped using 1 μL of 50 nmol ethylenediaminetetraacetic acid (EDTA) at 65°C for 10 min. After DNase treatment, the next step was the synthesis of cDNAs from mRNA, using the SuperScript IV Reverse Transcriptase kit (Invitrogen, Carlsbad, CA, USA) according to the manufacturer's instructions. Finally, the cDNA concentration was estimated with a NanodropTM UV‐Vis spectrophotometer (Thermo Scientific) at a wavelength of 260 nm.

### 2.4 Primers

For heminested PCR and qPCR reactions, we used the primer common to Dengue 1‐4 and ZIKV viruses previously described by Chien et al. [19] named mD1‐F (5′‐TCAATATGCTGAAACGCGAGAGAAACCG‐3′). ZIKV‐specific primers designed in the C‐prM region by Sá‐Guimarães et al. [20] were also used: GTZK‐F (5′‐GTGTAAACCCCTTGGGAGGTTT‐3′) and STZK‐R (5′‐AAGTTGGTAGCAAAGGAGATGGC‐3′). These primers are like those described by Lanciotti et al. [21] and Salles et al. [22].

### 2.5 Synthetic ZIKV Plasmid and Virus Cloning in pGEM®‐T Easy Vector Systems

A synthetic ZIKV plasmid, 815 bp in length, was designed for the C‐prM region (Epoch Life Science) to serve as a positive control for heminested PCR and qPCR techniques. Genomic fragments from 29 clinical and mosquito samples were amplified using GoTaq® DNA polymerase enzyme (Promega) and cloned into the pGEM®‐T Easy Vector System, following the manufacturer's protocol. The cloning vector was introduced into *Escherichia coli* strain DH5‐α using the heat shock method. The transformed bacteria were incubated in Luria‐Bertani (LB) broth (Sigma‐Aldrich, St. Louis, USA) with agitation at 37°C for 1 h at 120 RPM in a refrigerated shaking incubator (TE‐424 Tecnal, Piracicaba, Brazil). After incubation, the bacterial suspension was plated onto solid LB agar and incubated at 37°C for 24 h. Colonies harboring the plasmid were screened using colony PCR with primers described by Sá‐Guimarães et al. [20]. Selected positive colonies were re‐cultivated in LB broth supplemented with 100 µg/mL ampicillin at 37°C for 24 h as modified from Figueira‐Mansur et al. [23]. Plasmid extraction was performed using the QIAprep Spin Miniprep Kit (Qiagen, Hilden, Germany) according to the manufacturer's instructions. Finally, plasmid concentration was quantified using a UV‐Vis Nanodrop™ spectrophotometer (Thermo Scientific) at a wavelength of 260 nm.

### 2.6 Heminested PCR

ZIKV detection by hemi‐nested PCR followed the protocol established by Sá‐Guimarães et al. [20], consisting of two reactions with a total reaction volume of 20 μL. In the first reaction, 1 μL of cDNA, 50 nmol of MgCl₂, 50 nmol of specific primers (mD1‐F/STZK‐R), and reagents from the Phusion High‐Fidelity DNA Polymerase Kit (Thermo Fisher, Waltham, MA, USA) were used according to the manufacturer's instructions. The amplification cycle included the following steps: 98°C for 3 min, 35 cycles of 98°C for 10 s, 54°C for 10 s, and 72°C for 30 s, final extension at 72°C for 10 min. In the second reaction, the product from the first reaction served as the template for amplification. One μL of the product and 50 nmol of specific primers (GTZK‐F/STZK‐R), were used. The amplification protocol for this reaction followed these steps: 98°C for 3 min, 25 cycles of 98°C for 10 s, 54°C for 10 s and 72°C for 30 s. Final extension at 72°C for 10 min. After each amplification reaction, a 5 μL aliquot of the product was analyzed by 1.5% agarose gel electrophoresis. ZIKV detection was confirmed by the presence of amplification products of specific sizes.

### 2.7 Quantitative PCR (qPCR)

The qPCR methodology using SYBR Green was performed on clinical and mosquito samples, with ZIKV cDNA and a synthetic ZIKV plasmid used as positive controls. The presence of ZIKV was evaluated using a primer pair that amplifies the C‐prM region of the Zika virus (GTZK‐F and STZK‐R) at a concentration of 400 nmol. All tests were performed in triplicate, as established by Sá‐Guimarães et al. [20]. The reactions were carried out using the SsoAdvanced Universal SYBR Green Supermix enzyme (Bio‐Rad, Hercules, CA, USA) according to the manufacturer's instructions. The amplification cycle consisted of the following steps: 98°C for 3 min, 40 cycles of 98°C for 10 s, 54°C for 15 s, and 72°C for 30 s. The reactions were run on the CFX96 Touch thermal cycler (Bio‐Rad, Hercules, CA, USA). To confirm the identity of the amplified products, a melting curve analysis was performed after amplification, as described by Morrison et al. [24]. The melting curve analysis included: a denaturation cycle at 65°C for 5 s, followed by a ramp to 95°C at a rate of 0.5°C/10 s, with continuous fluorescence measurements.

### 2.8 Samples Sequencing

The 29 ZIKV‐positive samples from heminested PCR and qPCR, including clinical and mosquito samples, were purified from agarose gel using the QIAquick Gel Extraction Kit (Qiagen, Hilden, Germany). The purified PCR products were cloned and further processed. Each purified product was then mixed with the GTZK‐F primer and the STZK‐R primer in individual tubes. Sequencing was performed using the ABI PRISM BigDye Terminator Cycle Sequencing Ready Reaction Kit and the ABI PRISM DNA Analyzer 3730 (Applied Biosystems, USA) at the DNA Sequencing Platform PDTIS/FIOCRUZ [25]. The obtained nucleotide sequences were assessed for quality and edited using Chromas (Technelysium DNA Sequencing Software). The sequences were then compared using the Basic Local Alignment Search Tool (BLASTn) from the NCBI‐NIH database to confirm their similarity to ZIKV.

### 2.9 Phylogenetics Reconstruction and DNA Variant Analysis

The 29 partial ZIKV sequences were aligned with a global dataset of 797 complete ZIKV genome sequences using the MAFFT software. A maximum likelihood phylogeny was constructed with IQtree software, employing the GTR + F + I + G4 substitution model. The phylogenetic tree was supported by 1000 bootstrap replications. Genomic variants within the ZIKV lineages were analyzed and compared to the reference strain MR766 (KU720415.1). This analysis included the identification of single nucleotide polymorphisms (SNPs) as well as other types of genetic variations, such as insertions and deletions (indels). The SNP matrix was generated using Geneious Prime 2023.0 software (Biomatters, Auckland, New Zealand). Nonsynonymous SNPs and other nonsense mutations, including insertions and deletions, were investigated across the genome.

## 3 Results

**FIGURE 1:**
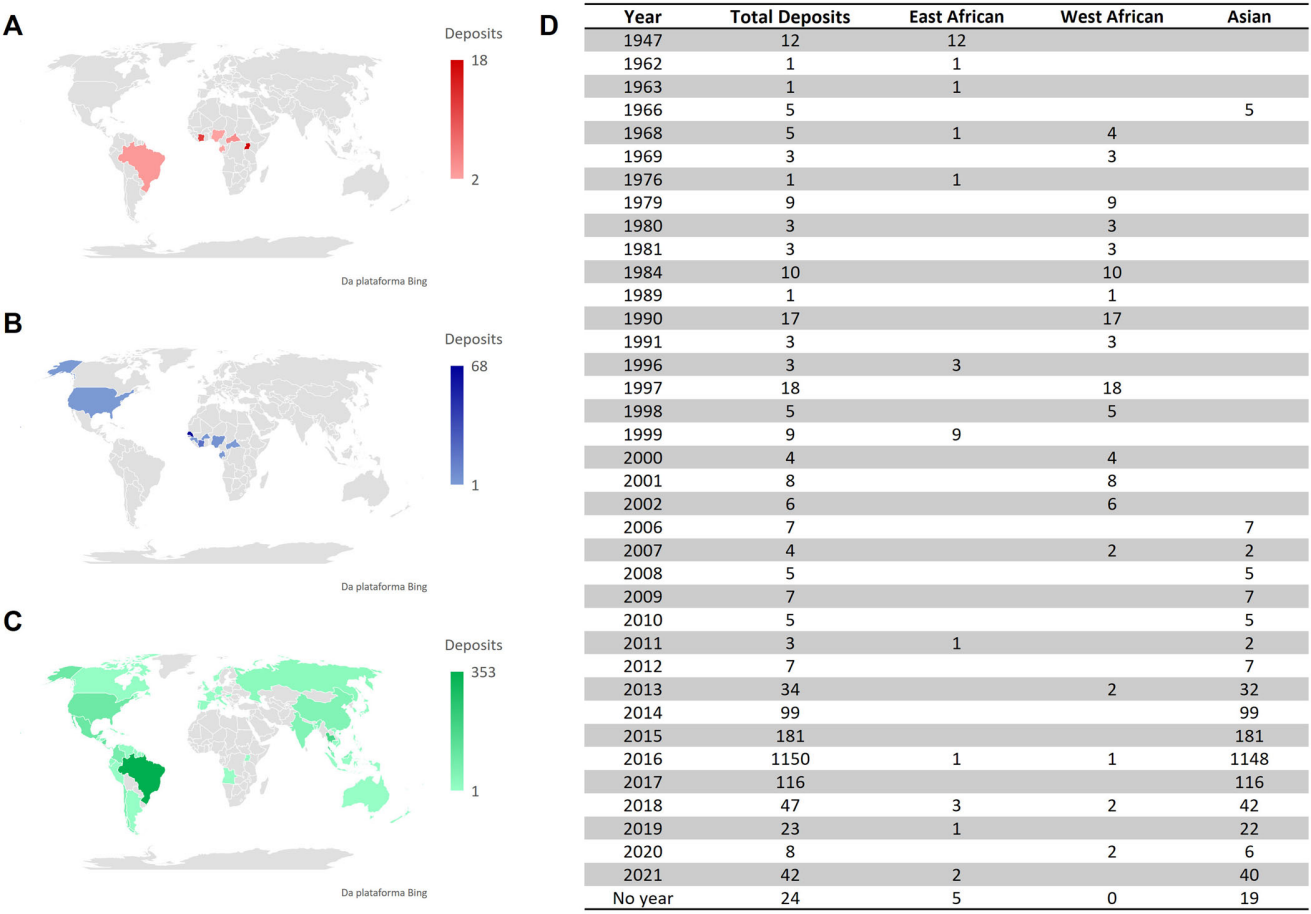
Distribution of ZIKV lineage deposits in the world from 1947 to October 2022. (A) Deposits referring to East African Lineage. (B) Deposits referring to West African lineage. (C) Deposits referring to Asian lineage. (D) Deposits of each ZIKV strain distributed by year.

In 27th of October 2022, we searched for available ZIKV sequences on the NCBI database to better comprehend the global spread of ZIKV. Using the keyword “Zika virus,” a total of 5523 sequences were identified (Figure 1A–D). Of these, 3634 sequences (66%) were excluded from the analysis as they did not meet the inclusion criteria. The remaining 1889 sequences (34%) comprising 833 complete sequences (44%) and 1056 partial sequences (56%) were used to analyze the spatiotemporal distribution of ZIKV and to observe the prevalence of each lineage from 1947 to October 2022, following the methodology of Giovanetti et al. [26] and Liang et al. [27]. Our prospection observed that over the course of 75 years, the ZIKV sequences were categorized into two main lineages: the Asian lineage (92%) and the African lineage (8%). The African lineage was further subdivided into West African (6%) and East African (2%).

In overall, the 1889 selected sequences are distributed from the year 1947 to 2021, with the highest concentration observed between 2013 and 2019 for the Asian lineage (Figure 1D). This period encompasses the three majors known ZIKV epidemics: 2007 on the island of Yap (Micronesia), 2013–2014 in French Polynesia, and 2015–2016 in the Americas [3, 15, 28]. The West African lineage was predominantly observed circulating between 1968 and 2007, with additional deposits in 2013, 2016, 2018, and 2020. In contrast, deposits for the East African lineage were concentrated primarily in 1947 and 1999, with sporadic entries in 1962, 1963, 1968, 1996, 2011, 2016, 2018, 2019, and 2021. These findings highlight the dominance of the Asian lineage during major ZIKV outbreaks. However, they also demonstrate the continued circulation of African lineages, despite their lack of association with the major known epidemics.

Most sequence deposits originate from the Americas (1124 sequences; 59%), followed by Asia (503 sequences; 27%) and Africa (148 sequences; 8%). Together, these three continents account for 94% of the total sequences analyzed in this study. The origins of the selected deposits were categorized into five groups: Human (1,454; 77%), animal (37; 2%), mosquito (280; 15%), laboratory (22; 1%), and unlisted (96; 5%). Regarding lineage distribution among these host categories, disparities were observed. In the Asian lineage, human samples predominated (1440 deposits), followed by mosquito isolates (265 deposits) and animal samples (27 deposits). In the West African lineage, sequences were distributed across three categories: humans (10 deposits), mosquitoes (10 deposits), and unlisted (79 deposits), with no isolates obtained from animals. Conversely, the East African lineage was primarily isolated from animals (10 deposits), followed by mosquito isolates (five deposits) and human samples (four deposits). The lack of detailed information about certain deposits complicates the identification of the primary vector sources and potential reservoirs for ZIKV.

In Brazil, 355 ZIKV sequences were deposited between 2015 and 2019, with two deposits lacking information about the year of collection. These deposits include 123 complete sequences (35%) and 232 partial sequences (65%), distributed between the East African lineage (three deposits; 1%) and the Asian lineage (352 deposits; 99%). No deposits of the West African lineage were identified in Brazil. Human fluid samples constituted many deposits (311; 88%), followed by mosquito samples (31 deposits; 9%), animal samples (nine deposits; 2%), and laboratory‐origin samples (four deposits; 1%). The peak of deposits occurred during the 2015–2016 ZIKV epidemic, with 79 deposits in 2015 and 215 deposits in 2016, followed by a decline from 2017 to 2019. Of the three deposits classified as the East African lineage, two originated from animals (*Alouatta guariba*) in 2018 and 2019 in the state of Rio Grande do Sul, and one originated from a mosquito (*Aedes albopictus*) in 2019 in the state of Rio de Janeiro [17, 18].

A global dispersion of ZIKV was previously reconstructed and analyzed using the phylogenetic method maximum likelihood [29]. Their study identified two main clades of ZIKV genomes: one composed of the African lineages (East and West) and the other of the Asian lineage. We analyzed viral dispersion using sequence deposits from NCBI, as described by Kasprzykowski et al. [30], to investigate the possible prior circulation of African lineages (East and West) in Brazil before the 2015–2016 Zika fever epidemic. Additionally, we aimed to understand how the sequences obtained from clinical samples and mosquitoes aligned with the East African lineage clade.

According to Chien et al. [19], the C‐prM region was identified as the most genetically diverse among flaviviruses. Hence, we selected the C‐prM region for analysis, using the MR766 strain (East African lineage; Accession Number: NC_012532) as the reference genome. Our objective was to determine the potential for differentiating the three ZIKV lineages (East African, West African, and Asian) through maximum likelihood analysis. Through the analysis of polymorphisms in the C‐prM region, we examined the genetic variability of ZIKV using 29 sequences from this study (Table S2) alongside 797 selected sequences from the 1889 identified ZIKV sequences. These sequences represent all three ZIKV lineages and were analyzed to determine molecular epidemiology, evolution, and phylogeny globally, with a focus on Brazil.

In general, partial sequences are avoided in phylogenetic analyses because they may introduce bias and reduce resolution [31]. However, due to the limited availability of complete ZIKV genomes in public databases, especially from animal hosts, partial sequences covering informative genomic regions were included always respecting the established criteria. This approach increased the representativeness of the dataset and enabled a broader understanding of ZIKV lineage diversity. Our results showed that the African lineages (East and West) and the Asian lineage show a different distribution. We identified African lineages circulating outside of Africa through the analysis of partial sequences. It was found West African sequences deposited in the USA (two deposits) and in East African sequences deposited in Brazil (three deposits).

To better understand the genetic basis underlying this lineage distribution, we next analyzed single nucleotide polymorphisms (SNPs) within the C‐prM region. The alignment of the 826 sequences (Figure S1) revealed 111 single nucleotide polymorphisms (SNPs) in the C‐prM region (Table 1). The SNPs were further evaluated to predict their effects on the biological structure of amino acids and proteins. The analysis included mutations specific to each lineage (Asian, East African, and West African) and mutations from samples compared across all three lineages.

**TABLE 1:** Analysis of ZIKV C‐prM region polymorphisms within 826 strains including this study representative sequences of Brazil.

Allele position (Ref.)	Locus	Codon change				Type of Polymorphism	Effect on amino acid^(*)^	Effect on protein
		East	West	Asian	Samples			
15	C	G	G	A	—	SNP (Transversion)	K → N	Substitution
72	C	A	A	G	A	SNP (Transition)	—	—
74	C	A	A	G	A	SNP (Transition)	N → S	Substitution
81	C	G	G	T	T	SNP (Transversion)	L → F	Substitution
84	C	A	G	G	A	SNP (Transition)	—	—
87	C	T	T	C	T	SNP (Transition)	—	—
126	C	A	A	G	A	SNP (Transition)	—	—
135	C	A	A	G	G	SNP (Transition)	—	—
141	C	T	T	C	T	SNP (Transition)	—	—
147	C	G	G	G	A	SNP (Transition)	—	—
150	C	A	A	T	A	SNP (Transversion)	—	—
168	C	C	T	T	C	SNP (Transition)	—	—
171	C	A	A	G	A	SNP (Transition)	—	—
192	C	C	C	T	C	SNP (Transition)	—	—
201	C	C	T	T	C	SNP (Transition)	—	—
211–213	C	ACC	TCC	TCA	TCT	Substitution	T → S	Substitution
222	C	A	G	A	A	SNP (Transition)	—	—
225	C	A	G	A	A	SNP (Transition)	—	—
252	C	C	C	C	T	SNP (Transition)	—	—
261	C	T	C	T	T	SNP (Transition)	—	—
264	C	T	T	G	T	SNP (Transversion)	—	—
274	C	T	T	C	T	SNP (Transition)	—	—
282	C	A	T	A	A	SNP (Transversion)	—	—
297	C	A	G	G	G	SNP (Transition)	—	—
302	C	G	G	A	G	SNP (Transition)	R → K	Substitution
312	C	T	T	A	T	SNP (Transversion)	—	—
315	C	C	C	C	T	SNP (Transition)	—	—
312	C	T	T	A	T	SNP (Transversion)	—	—
324	C	C	C	T	C	SNP (Transition)	—	—
327	C	C	C	T	C	SNP (Transition)	—	—
328	C	A	A	G	A	SNP (Transition)	I → V	Substitution
336	C	C	C	T	C	SNP (Transition)	—	—
337	C	A	G	G	A	SNP (Transition)	V → I	Substitution
339	C	T	C	T	T	SNP (Transition)	—	—
342	C	T	T	T	C	SNP (Transition)	—	—
354	C	T	C	C	T	SNP (Transition)	—	—
360	C	C	C	T	C	SNP (Transition)	—	—
369	C	A	C	G	G	A → C SNP (Transversion)	—	—
						A → G SNP (Transition)		
373	C	A	A	G	A	SNP (Transition)	I → V	Substitution
375	C	C	C	C	T	SNP (Transition)	—	—
378	C	T	T	T	C	SNP (Transition)	—	—
387	PR	G	G	G	A	SNP (Transition)	—	—
399	PR	C	C	T	C	SNP (Transition)	—	—
411	PR	T	C	C	C	SNP (Transition)	—	—
414	PR	G	G	A	G	SNP (Transition)	—	—
423	PR	C	T	T	T	SNP (Transversion)	—	—
426	PR	G	T	G	G	SNP (Transversion)	—	—
427	PR	A	G	G	A	SNP (Transition)	K → E	Substitution
435	PR	T	T	A	C	T → A SNP (Transversion)	—	—
						T → C SNP (Transition)		
442–444	PR	GCT	GCT	CCA	—	Substitution	A → P	—
444	PR	T	C	A	—	T → A SNP (Transversion)	—	—
						T → C SNP (Transition)		
451	PR	T	T	C	—	SNP (Transition)	—	—
457	PR	G	G	A	—	SNP (Transition)	V → M	Substitution
462	PR	C	C	G	—	SNP (Transition)	—	—
465	PR	G	A	G	—	SNP (Transition)	—	—
468	PR	C	C	T	—	SNP (Transition)	—	—
469	PR	C	C	T	—	SNP (Transition)	H → Y	Substitution
472–474	PR	GTG	GTG	ATA	—	Substitution	V → I	Substitution
486	PR	C	C	T	—	SNP (Transition)	—	—
489	PR	C	C	T	—	SNP (Transition)	—	—
492	PR	G	G	A	—	SNP (Transition)	—	—
504	PR	C	C	T	—	SNP (Transition)	—	—
516	PR	C	C	T	—	SNP (Transition)	—	—
522	PR	A	A	G	—	SNP (Transition)	—	—
528	PR	T	C	T	—	SNP (Transition)	—	—
534	PR	G	A	G	—	SNP (Transition)	—	—
537	PR	T	C	T	—	SNP (Transition)	—	—
543	PR	A	A	G	—	SNP (Transition)	—	—
549	PR	A	G	A	—	SNP (Transition)	—	—
558	PR	C	T	C	—	SNP (Transition)	—	—
567	PR	C	C	T	—	SNP (Transition)	—	—
582	PR	A	A	G	—	SNP (Transition)	—	—
585	PR	A	G	A	—	SNP (Transition)	—	—
609	PR	T	T	C	—	SNP (Transition)	—	—
615	PR	C	T	C	—	SNP (Transition)	—	—
627	PR	G	A	A	—	SNP (Transition)	—	—
633	PR	G	A	G	—	SNP (Transition)	—	—
634	PR	C	C	A	—	SNP (Transversion)	—	—
639	PR	T	C	T	—	SNP (Transition)	—	—
648	M	C	C	T	—	SNP (Transition)	—	—
657	M	C	T	C	—	SNP (Transition)	—	—
660	M	T	T	C	—	SNP (Transition)	—	—
663	M	T	T	C	—	SNP (Transition)	—	—
666	M	C	C	T	—	SNP (Transition)	—	—
669	M	T	C	C	—	SNP (Transition)	—	—
672	M	A	G	T	—	A → T SNP (Transversion)	—	—
						A → G SNP (Transition)		
678–679	M	GT	GC	GC	—	Substitution	—	—
699	M	C	T	C	—	SNP (Transition)	—	—
703	M	C	T	C	—	SNP (Transition)	—	—
705	M	A	A	G	—	SNP (Transition)	—	—
708	M	A	G	A	—	SNP (Transition)	—	—
730	M	T	C	T	—	SNP (Transition)	—	—
735	M	C	C	T	—	SNP (Transition)	—	—
737–738	M	AG	AG	GA	—	Substitution	K → R	Substitution
741	M	T	T	C	—	SNP (Transition)	—	—
747	M	C	T	T	—	SNP (Transition)	—	—
765	M	C	C	T	—	SNP (Transition)	—	—
768	M	G	G	C	—	T → G SNP (Transversion)	—	—
						T → C SNP (Transition)		
771–772	M	TG	TG	CG	—	Substitution	FT → FA	Substitution
775	M	C	C	T	—	SNP (Transition)	—	—
779	M	T	T	C	—	SNP (Transition)	V → A	Substitution
780	M	G	G	A	—	SNP (Transition)	—	—
783	M	C	T	A	—	T → A SNP (Transversion)	—	—
						T → C SNP (Transition)		
785	M	T	T	C	—	SNP (Transition)	—	—
789	M	C	T	C	—	SNP (Transition)	—	—
795	M	C	C	T	—	SNP (Transition)	—	—
801	M	T	C	T	—	SNP (Transition)	—	—
802	M	T	C	T	—	SNP (Transition)	—	—
813	M	G	G	A	—	SNP (Transition)	—	—
828	M	C	T	C	—	SNP (Transition)	—	—
847	M	C	T	C	—	SNP (Transition)	—	—
850	M	C	T	C	—	SNP (Transition)	—	—

Abbreviations: (*)A, Alanine; C, Cysteine; D, Aspartic acid; E, Glutamic acid; F, Phenylalanine; G, Glycine; H, Histidine; I, Isoleucine; K, Lysine; L, Leucine; M, Methionine; N, Asparagine; P, Proline; Q, Glutamine; R, Arginine; S, Serine; T, Threonine; V, Valine; W, Tryptophan; Y, Tyrosine.

Of the total 111 SNPs, 47 were identified in the 29 samples collected in Brazil, with 16 SNPs causing amino acid substitutions in proteins: 15 K → 15 N, 74 N → 74S, 81 L → 81 F, 211‐213 T → 211‐213S, 302 R → 302 K, 328I → 328 V, 337 V → 337I, 373I → 373 V, 427 K → 427E, 442‐444 A → 442‐444 P, 457 V → 457 M, 469H → 469Y, 472‐474 V → 472‐474I, 737‐738 K → 737‐738 R, 771‐772F‐T → 771‐772F‐A, and 779 V → 779 A. It should be noted that alleles 211‐213 differ across the four sequence sets (samples, East African, West African, and Asian lineages). The 81 allele in the sequenced samples is identical to the Asian lineage, while 373 and 427 alleles show SNPs consistent with those found in the East African lineages. Among the 111 SNPs, 58 differ between the African (East and West) and Asian lineages. Of these, 12 SNPs result in amino acid substitutions in proteins: 15, 74, 81, 302, 328, 373, 442–444, 457, 469, 472–474, 771–772, and 779.

These collective results suggest that an East African ZIKV strain circulated undetected in Brazil between 2017 and 2020. Our results on genetic polymorphisms in the C‐prM region of complete sequence files deposited in NCBI, along with the ZIKV samples sequenced in the present work, reinforces the possibility of co‐circulation of different ZIKV strains, including in humans.

## 4 Discussion

In 2007, ZIKV was recognized as an emerging virus following an outbreak in Micronesia and reemerged in 2013 during an epidemic in French Polynesia [32]. In 2015, the first autochthonous case of ZIKV in Brazil was reported in the Northeast region. By 2016, the virus had spread across all 26 states and the Federal District, establishing Brazil as the epicenter of the most recent epidemic in the Americas and the Caribbean [33, 34]. The increase in reported cases of neurological complications in tropical and subtropical regions, particularly beginning in 2015, garnered global attention to this virus [5, 32, 35, 36].

The lineages distribution of ZIKV strains may be attributed to strain differentiation events. Senegal has been identified as a primary source of ZIKV dissemination beyond the African continent, facilitating its migration within Africa and subsequent spread to Asia. The passage of the virus through various hosts is likely a key driver of differentiation, contributing to changes in the clinical manifestations of ZIKV infection. For instance, it is hypothesized that this process led to a potential “attenuation” in infection severity in pregnant women infected with the Asian lineage. The African lineages are theorized to cause a higher incidence of abortions, whereas the Asian lineage is predominantly associated with fetal abnormalities such as microcephaly and congenital Zika virus syndrome [14, 15, 37, 38].

The Asian lineage was found to be present across all continents except Antarctica, with a high concentration in the Americas and Asia, making it the only lineage with a globally distributed range for ZIKV [16]. In contrast, sequences identified as African lineages were exclusively deposited from Africa until 2018–2019, after which the East African lineage was detected in the South and Southeast regions of Brazil [17, 18]. This difference in lineage distribution may be attributed to the adaptation of the Asian lineage to new vectors and transmission routes, as well as the absence of specific surveillance systems for ZIKV before 2007 [14, 16, 39].

The Asian lineage is widely described in the literature as the sole circulating lineage during the ZIKV public health emergency in 2016 [40]. However, the findings of Almeida et al. [18], Alencar et al. [17], and Kasprzykowski et al. [30] suggest that the East African lineage was introduced into Brazil and is already being maintained in the wild. These results, combined with the findings of this study, indicate that the East African lineage may have circulated undetected during the epidemic and possesses the potential to cause a new epidemic. Viruses exhibit significant genetic variability due to mechanisms such as mutations, gene duplication, genome reorganization, and recombination. Rapid reproduction, high mutation rates, and gene recombination are hallmarks of viral evolution. In phylogenetic analyses, mutations, deletions, and insertions are utilized to infer genetic relationships, describe strain distribution in populations, and assess host and parasite risk factors in disease transmission.

The investigation of polymorphisms through molecular epidemiology offers valuable insights with direct impacts on medicine and public health. Such research facilitates the development of novel diagnostic tools and intervention methods [41]. The C‐prM region has been identified as the most genetically diverse region among flaviviruses [19], making it an ideal genomic target for differentiation. This region supports the identification of various flaviviruses within the same genomic region. Similarly, Cox et al. [42] emphasized the importance of the prM, E, NS1, NS3, and NS5 proteins in key functions related to ZIKV pathogenesis and infectivity.

African lineages were identified as responsible for ZIKV outbreaks until 2001. However, starting from 2007, the Asian lineage was primarily identified in countries in the Americas and Asia, and was considered the cause of recent epidemics in Micronesia, French Polynesia, New Caledonia, and Brazil [3, 4, 5, 16, 43, 44].

**FIGURE 2:**
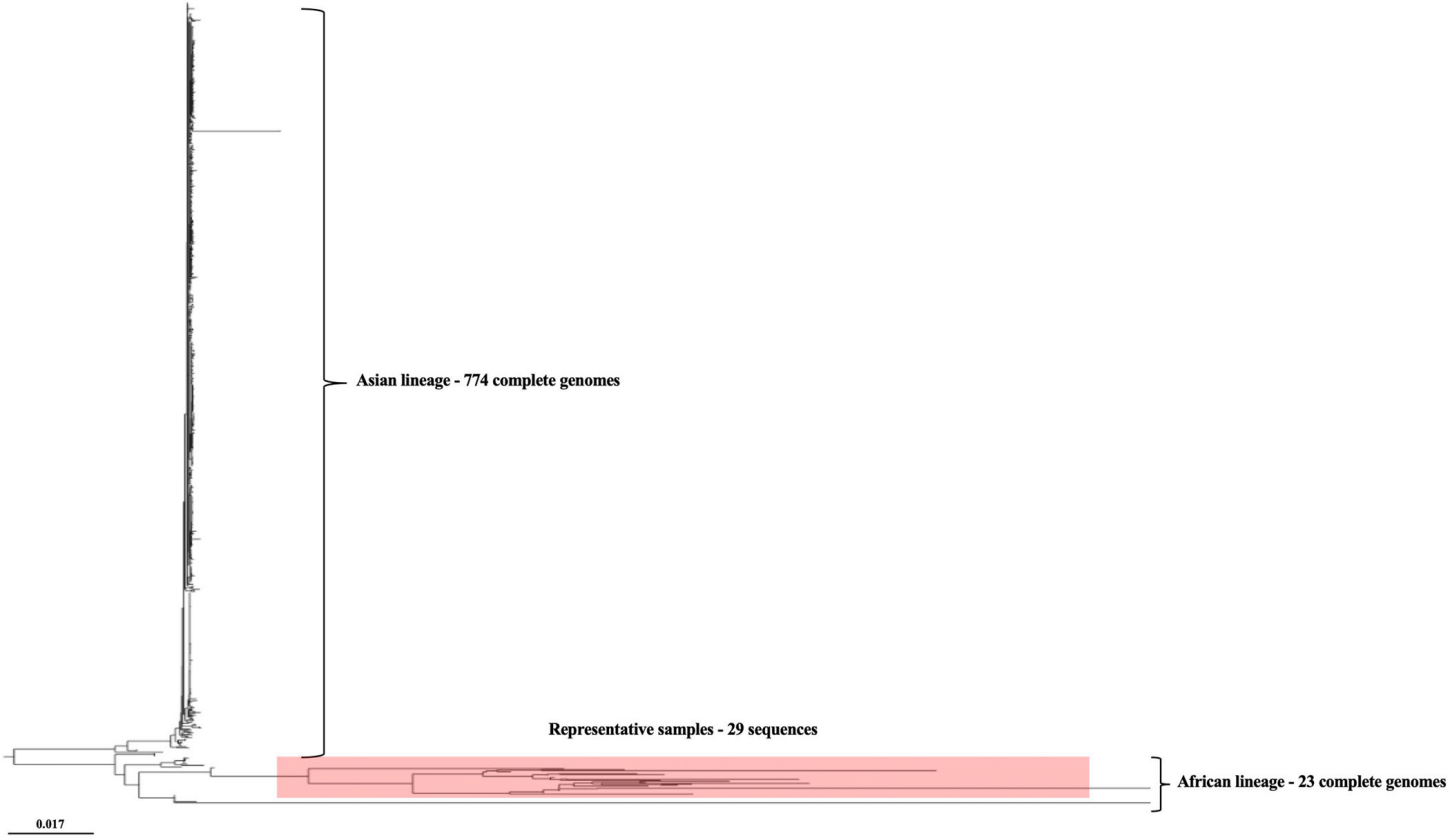
Maximum likelihood phylogenetic tree based on the analysis of 797 complete ZIKV genomes obtained at NCBI and 29 partial sequences of the ZIKV C‐prM region.

ZIKV has been divided into two distinct clades: one comprising the Asian lineage and the other including the two African lineages (Figure 2). This division aligns with results observed by Ebranati et al. [29]. Our study found that the sequenced samples from humans and mosquitoes were grouped within the East African lineage clade. This finding is corroborated by the work of Kasprzykowski et al. [30], where ZIKV sequences obtained from the Southern region of Brazil, in Rio Grande do Sul State [17], and from the Southeastern region, in Rio de Janeiro State [18], were also grouped within the East African lineage clade. These regions, separated by more than 1500 km, comprised samples originating from monkeys and mosquitoes naturally infected with the virus, respectively.

Shen et al. [16], based on the analysis of the sequences of the E and NS5 proteins, support the hypothesis that the three ZIKV lineages evolved separately and that recombination events contributed to the phylogenetic differences observed between ZIKV lineages. It is speculated that the constant exchange (import and export) of strains between Senegal and Côte d'Ivoire corresponds to the observed recombination events and may have contributed to the formation of distinct genotypes [45]. The study of polymorphism in ZIKV is essential to understanding the evolution of virus and its relative degree of infectiousness likely happened with Dengue virus.

The spread of ZIKV throughout the Pacific and the Americas led to the selection of new mutations, some of which may be associated with increased infectivity and virulence [46]. However, Zhao et al. [47] suggest that mutations observed during the epidemic in Brazil did not affect the virus's replication or virulence. Notably, a single mutation, the Ser139→Asn139 mutation in the prM protein, has been shown to influence ZIKV neurotropism, potentially contributing to the increased incidence of microcephaly observed in Brazil and French Polynesia [48]. The study on ZIKV evolution and spread in the Americas [31] identified mutations with potential functional implications for ZIKV biology, pathogenesis, and the effectiveness of diagnostic tests. These authors found numerous sites where the probe or primer did not match alleles present in the 174 ZIKV genomes analyzed. In most cases, the discordant allele was shared by all outbreak samples, likely due to its presence in the Asian lineage that entered the Americas. These mismatches could affect the performance of diagnostic assays during the outbreak. Additionally, mismatches from new mutations arising after ZIKV's entry into the Americas were identified. Most of these mutations were present in less than 10% of samples, although one was observed in 29% of samples. These observations suggest that genome evolution has not caused widespread degradation in diagnostic performance during the outbreak. However, ongoing monitoring is necessary as mutations continue to accumulate. In general, the Asian lineage is associated with neurotropism, leading to microcephaly and other congenital anomalies, while strains from African lineages are linked to miscarriages when infections occur early in pregnancy [42, 49]. Our analysis also showed that ZIKV lineages are > 95% identical.

However, differentiation between the lineages is better achieved through analysis of the E protein, as African lineages exhibit a deletion of six amino acids in the 155–160 region, which can serve as a criterion to distinguish African lineages from Asian lineages [42, 49]. Additionally, mutations in protein C, such as the substitution of threonine for alanine at the 106th residue described by Yu et al. [50], have been associated with increased viral infectivity in hosts (mammals) and vectors (mosquitoes). This mutation may enhance the ZIKV epidemic by facilitating transmission cycles. Ye et al. [51] identified 19 amino acid substitutions in sequences coding for ZIKV structural proteins, whereas we observed 16 substitutions in the C‐prM region (Table 1—Column “Effect on Amino Acid”). This discrepancy is likely due to the completeness of the sequenced regions. It was utilized 58 complete sequences from NCBI, whereas we employed Sanger sequencing for the C‐prM region, where the precise start and end of sequencing are less well‐defined. This may have resulted in the non‐identification of three substitutions compared to those described by Ye et al. [51].

The genetic differences observed in the C‐prM region in this study highlight that variations among ZIKV genotypes may contribute to differences in virulence and epidemic potential, similar to what has been demonstrated for dengue virus (DENV) [52]. Wang et al. [53] reported that the prM region exhibited the highest percentage of variability among African and Asian lineages, which may correlate with differences in the pathogenesis of the Asian lineage. The exclusive polymorphisms of Brazilian‐East African sequences likely emerged due to environmental and host‐imposed selective pressures, driving modifications in antigenic properties, biology, and pathogenesis of ZIKV [28].

Findings of ZIKV in monkeys [18] and mosquitoes [17], along with partial ZIKV sequences isolated from mosquitoes, underscore the need to expand knowledge about ZIKV's enzootic cycle. A study by Vasilakis et al. [54] suggested that the emergence of endemic DENV‐2 strains from sylvatic strains did not require significant adaptation to replicate efficiently in human hosts. Similarly, the re‐emergence of human‐to‐human transmission of sylvatic DENV‐2 strains may occur without substantial genetic or phenotypic changes. ZIKV, like DENV, appears to thrive within a specific range of vertebrate hosts, primarily primates, which act as amplification and reservoir hosts. Whether Asia, like Africa, has *enzootic foci* of ZIKV circulation remains uncertain and would require studies in remote forests away from human‐mosquito interactions or evidence of evolutionarily independent ZIKV lineages associated with enzootic cycles, as documented for dengue viruses [53].

A significant limitation of this study was the inability to perform whole genome sequencing (WGS) on the analyzed samples due to RNA degradation, resulting from storage conditions that were not designed for long‐term genomic studies. The genomic material was of insufficient quality to generate complete genomes from the 2015 to 2017 samples obtained from humans and mosquitoes. Furthermore, despite extensive sampling efforts between 2017 and 2022, no specimens in adequate condition for WGS were found. This limitation, as also noted by Metsky et al. [31], highlights the inherent challenges of retrospective studies that rely on archived biological material. However, through our examination of sequences deposited in NCBI, we were able to highly accurate compare C‐prM region of the emergent ZIKV, lineage diversity and global distribution. Complementary analyses indicated that many incomplete sequences could be assigned to the Asian lineage supporting its global dominance. Most importantly, our data indicate the emergence of a new ZIKV lineage in Brazil, belonging to the East African lineage, confirmed in silico analyses, polymorphism assessments in the C‐prM region and sequencing results. This lineage was also confirmed through sequences obtained from mosquitoes and nonhuman primates.

In conclusion, although new sequence data were limited to Brazilian samples only, our findings highlight the contribution of regional studies in the efforts to understand ZIKV lineage dynamics. Overall, this study serves as an important reminder of the urgent need for continued monitoring and sequencing efforts to better characterize ZIKV biology and pathogenesis, especially as new lineages arise and spread, which may have implications for public health. In future, the confirmation of the East African lineage with available ZIKV samples could reshape the understanding of ZIKV epidemiology in Brazil, addressing many unresolved questions from the ZIKV outbreak. Additional sequencing, though challenging, remains essential for further investigations into ZIKV biology and pathogenesis. Genomic surveillance, integrating genomic, clinical, and epidemiological data, is crucial for tracking variant emergence, viral spread, and ensuring sustainable monitoring of viruses such as ZIKV.

## Author Contributions

**Thayane da Encarnação Sá‐Guimarães:** investigation, validation, formal analysis, conceptualization, visualization, writing – original draft. **Pedro Panzenhagen:** investigation, writing – review and editing, software, formal analysis, visualization. **Anita Ferreira do Valle:** writing – review and editing. **Carlos Adam Conte Junior:** writing – review and editing. **Mônica Ferreira Moreira:** conceptualization, investigation, funding acquisition, supervision, writing – review and editing, visualization, validation, formal analysis, data curation.

## Conflicts of Interest

The authors declare no conflicts of interest.

## Supporting information


**Figure S1:** Illustrative image of the alignment of the C‐prM genomic region from the 826 sequences used in the polymorphism analysis of ZIKV lineages.

**Table S1:** Summary of Zika virus sequences retrieved from GenBank (https://www.ncbi.nlm.nih.gov/nuccore) from 1947 to October 2022. Metadata includes accession number, country and year of isolation, lineage, host, and genome type (complete or partial).

**Table S2:** Identification of the 29 ZIKV samples analyzed in this study based on the C‐prM region. Clinical samples were obtained from saliva, urine, and serum of infected individuals, while entomological samples were derived from mosquito pools (eggs, larvae, and adult mosquitoes).

## Data Availability

The data that support the findings of this study are available in the supporting material of this article.
