# Supplementary figures and images for: ZIKV Phylogenetic Characterization Reveals Evolutionary Diversity, Regional Dissemination, and Emergence of African Lineages in Brazil
Source: J Basic Microbiol. 2025 Oct 28;66(1):e70122. doi: 10.1002/jobm.70122 (PMC12706136; doi:10.1002/jobm.70122)

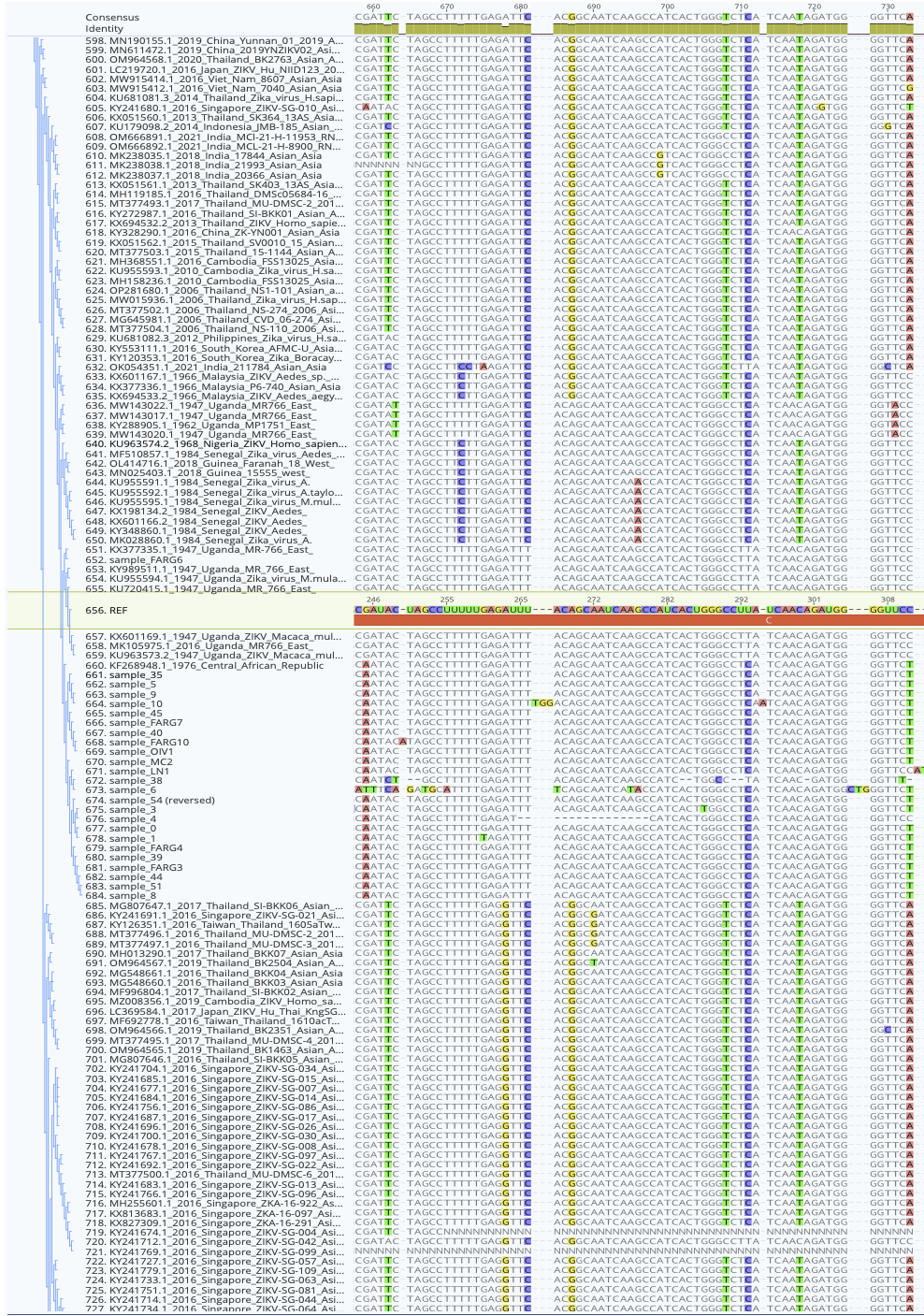

Supplement: Supplementary file 1 — Figure S1: Illustrative image of the alignment of the C‐prM genomic region from the 826 sequences used in the polymorphism analysis of ZIKV lineages. [file JOBM-66-e70122-s002.png]
