# Supplementary material for: ZIKV Phylogenetic Characterization Reveals Evolutionary Diversity, Regional Dissemination, and Emergence of African Lineages in Brazil
Source: J Basic Microbiol. 2025 Oct 28;66(1):e70122. doi: 10.1002/jobm.70122 (PMC12706136; doi:10.1002/jobm.70122)
Supplement: Supplementary file 2 — Table S1: Summary of Zika virus sequences retrieved from GenBank (https://www.ncbi.nlm.nih.gov/nuccore) from 1947 to October 2022. Metadata includes accession number, country and year of isolation, lineage, host, and genome type (complete or partial). [file JOBM-66-e70122-s001.docx]

| **GenBank Accessions** | **Collection Year** | **Isolation Country** | **Lineage** | **Host Group** | **Genome Status** | **Sequencing Platform** |
| --- | --- | --- | --- | --- | --- | --- |
| KU681081 | 2014 | Thailand | Asian | Human | Complete | Illumina |
| MT439645 | 2016 | Brazil | Asian | Human | Complete | MinIon - Oxford Nanopore |
| MT439646 | 2016 | Brazil | Asian | Human | Complete | MinIon - Oxford Nanopore |
| MT439647 | 2016 | Brazil | Asian | Human | Complete | MinIon - Oxford Nanopore |
| MT483911 | 2016 | Brazil | Asian | Human | Complete |  |
| MT505349 | 2020 | USA | West African | Laboratory | Complete | Sanger dideoxy sequencing |
| MT505350 | 2020 | USA | West African | Laboratory | Complete | Sanger dideoxy sequencing |
| MF629796 | 2011 | Nigeria | East African | Human | Partial | Sanger dideoxy sequencing |
| MF629797 | 2013 | Nigeria | West African | Human | Partial | Sanger dideoxy sequencing |
| MF629798 | 2013 | Senegal | West African | Human | Partial | Sanger dideoxy sequencing |
| MF629799 | 2000 | Senegal | West African | Human | Partial | Sanger dideoxy sequencing |
| KU681082 | 2012 | Philippines | Asian | Human | Complete | Illumina |
| MF664436 | 2016 | Russia | Asian | Human | Complete | Sanger dideoxy sequencing |
| MT507047 | 2017 | Mexico | Asian | Human | Complete | Illumina |
| MT507048 | 2017 | Mexico | Asian | Human | Complete | Illumina |
| MT507049 | 2017 | Mexico | Asian | Human | Complete | Illumina |
| MT507050 | 2017 | Mexico | Asian | Human | Complete | Illumina |
| MH916801 | 2016 | USA | Asian | Animal | Complete | Ion Torrent;Illumina |
| MF692778 | 2016 | Taiwan | Asian | Human | Complete | Sanger dideoxy sequencing |
| MH916802 | 2016 | USA | Asian | Animal | Complete | Ion Torrent;Illumina |
| MH916803 | 2016 | USA | Asian | Animal | Complete | Ion Torrent;Illumina |
| MH916804 | 2016 | USA | Asian | Animal | Complete | Ion Torrent;Illumina |
| KU686218 | 2015 | Mexico | Asian | Human | Partial | IonTorrent |
| MH916805 | 2016 | USA | Asian | Animal | Complete | Ion Torrent;Illumina |
| MH916806 | 2016 | USA | Asian | Animal | Complete | Ion Torrent;Illumina |
| KX117076 | 2016 | China | Asian | Human | Complete | Sanger dideoxy sequencing; IonTorrent |
| MF783072 | 2016 | Haiti | Asian | Mosquito | Complete | Sanger dideoxy sequencing |
| MH938334 | 2015 | Brazil | Asian | Human | Partial | Sanger dideoxy sequencing |
| MF783073 | 2016 | Haiti | Asian | Mosquito | Complete | Sanger dideoxy sequencing |
| MF794971 | 2016 | Ecuador | Asian | Human | Complete | Illumina |
| MF801377 | 2016 | El Salvador | Asian | Human | Partial | Illumina |
| MF801378 | 2016 | Guatemala | Asian | Human | Complete | Illumina |
| MF801379 | 2016 | Guatemala | Asian | Human | Partial | Illumina |
| KU707826 | 2015 | Brazil | Asian | Human | Complete | IonTorrent |
| MF801380 | 2016 | Guatemala | Asian | Human | Partial | Illumina |
| MF801381 | 2016 | Honduras | Asian | Human | Complete | Illumina |
| MF801382 | 2016 | Honduras | Asian | Human | Partial | Illumina |
| MF801383 | 2016 | Honduras | Asian | Human | Partial | Illumina |
| MF801384 | 2016 | Honduras | Asian | Human | Complete | Illumina |
| MF801385 | 2016 | Honduras | Asian | Human | Partial | Illumina |
| MF801386 | 2016 | Honduras | Asian | Human | Partial | Illumina |
| MF801387 | 2016 | Honduras | Asian | Human | Complete | Illumina |
| MF801388 | 2016 | Honduras | Asian | Human | Partial | Illumina |
| MF801389 | 2016 | Honduras | Asian | Human | Complete | Illumina |
| KU724096 | 2015 | Panama | Asian | Human | Partial | Sanger dideoxy sequencing |
| MF801390 | 2016 | Mexico | Asian | Human | Partial | Illumina |
| MF801391 | 2016 | Mexico | Asian | Human | Complete | Illumina |
| MF801392 | 2016 | Mexico | asian | Human | Partial | Illumina |
| MF801393 | 2016 | Mexico | Asian | Human | Partial | Illumina |
| MF801394 | 2016 | Mexico | Asian | Human | Partial | Illumina |
| MF801395 | 2016 | Mexico | Asian | Human | Complete | Illumina |
| MF801396 | 2016 | Mexico | Asian | Human | Complete | Illumina |
| KX156774 | 2015 | Panama | Asian | Human | Complete | Illumina |
| MF801397 | 2016 | Mexico | Asian | Human | Partial | Illumina |
| KX156775 | 2015 | Panama | Asian | Human | Complete | Illumina |
| KU720415 | 1947 | Uganda | East African | Laboratory | Complete | Illumina |
| MF801398 | 2016 | Mexico | Asian | Human | Complete | Illumina |
| KX156776 | 2015 | Panama | Asian | Human | Complete | Illumina |
| MF801399 | 2016 | Mexico | Asian | Human | Partial | Illumina |
| MF801400 | 2016 | Mexico | Asian | Human | Partial | Illumina |
| MF801401 | 2016 | Mexico | Asian | Human | Partial | Illumina |
| MF801402 | 2016 | Mexico | Asian | Human | Complete | Illumina |
| MF801403 | 2016 | Mexico | Asian | Human | Complete | Illumina |
| MF801404 | 2016 | Mexico | Asian | Human | Complete | Illumina |
| MF801405 | 2016 | Mexico | Asian | Human | Partial | Illumina |
| MF801406 | 2016 | Mexico | Asian | Human | Complete | Illumina |
| KU724097 | 2015 | Panama | Asian | Human | Partial | Sanger dideoxy sequencing |
| MF801407 | 2016 | Mexico | Asian | Human | Complete | Illumina |
| MF801408 | 2016 | Mexico | Asian | Human | Complete | Illumina |
| MF801409 | 2016 | Mexico | Asian | Human | Complete | Illumina |
| MF801410 | 2016 | Mexico | Asian | Human | Complete | Illumina |
| MF801411 | 2016 | Mexico | Asian | Human | Partial | Illumina |
| MF801412 | 2016 | Mexico | Asian | Human | Complete | Illumina |
| MF801413 | 2016 | Mexico | Asian | Human | Complete | Illumina |
| MF801414 | 2016 | Mexico | Asian | Human | Complete | Illumina |
| MF801415 | 2016 | Mexico | Asian | Human | Partial | Illumina |
| MF801416 | 2016 | Mexico | Asian | Human | Partial | Illumina |
| KU724098 | 2015 | Panama | Asian | Human | Partial | Sanger dideoxy sequencing |
| MF801417 | 2016 | Mexico | Asian | Human | Complete | Illumina |
| MF801418 | 2016 | Mexico | Asian | Human | Complete | Illumina |
| MF801419 | 2016 | Mexico | Asian | Human | Partial | Illumina |
| MF801420 | 2016 | Mexico | Asian | Human | Complete | Illumina |
| MF801421 | 2016 | Mexico | Asian | Human | Partial | Illumina |
| MF801422 | 2016 | Mexico | Asian | Human | Partial | Illumina |
| MF801423 | 2016 | Mexico | Asian | Human | Complete | Illumina |
| MF801424 | 2016 | Mexico | Asian | Human | Partial | Illumina |
| MF801425 | 2016 | Nicaragua | Asian | Human | Partial | Illumina |
| MF801426 | 2016 | Nicaragua | Asian | Human | Complete | Illumina |
| KU724099 | 2015 | Panama | Asian | Human | Partial | Sanger dideoxy sequencing |
| MF926508 | 2016 | Nigeria | West African | Human | Partial | Sanger dideoxy sequencing |
| MF964216 | 2016 | China | Asian | Mosquito | Partial | Sanger dideoxy sequencing |
| MN100039 | 2015 | Panama | Asian | Laboratory | Complete |  |
| MN101548 | 2017 | Brazil | Asian | Mosquito | Complete | IonTorrent |
| MK028538 | 2016 | Thailand | Asian | Mosquito | Partial |  |
| MK028539 | 2016 | Thailand | Asian | Mosquito | Partial |  |
| MK028540 | 2016 | Thailand | Asian | Mosquito | Partial |  |
| MK028541 | 2016 | Thailand | Asian | Mosquito | Partial |  |
| MK028542 | 2017 | Thailand | Asian | Mosquito | Partial |  |
| MK028543 | 2017 | Thailand | Asian | Mosquito | Partial |  |
| KU724100 | 2015 | Panama | Asian | Human | Partial | Sanger dideoxy sequencing |
| MK028544 | 2017 | Thailand | Asian | Mosquito | Partial |  |
| MK028545 | 2017 | Thailand | Asian | Mosquito | Partial |  |
| MK028546 | 2017 | Thailand | Asian | Mosquito | Partial |  |
| MK028547 | 2017 | Thailand | Asian | Mosquito | Partial |  |
| MK028548 | 2017 | Thailand | Asian | Mosquito | Partial |  |
| MK028549 | 2017 | Thailand | Asian | Mosquito | Partial |  |
| MK028550 | 2017 | Thailand | Asian | Mosquito | Partial |  |
| MK028551 | 2017 | Thailand | Asian | Mosquito | Partial |  |
| MK028552 | 2017 | Thailand | Asian | Mosquito | Partial |  |
| MK028553 | 2018 | Thailand | Asian | Mosquito | Partial |  |
| KU729217 | 2015 | Brazil | Asian | Human | Complete | Ion and 454 |
| MK028554 | 2018 | Thailand | Asian | Mosquito | Partial |  |
| MK028555 | 2017 | Thailand | Asian | Mosquito | Partial |  |
| MK028556 | 2017 | Thailand | Asian | Mosquito | Partial |  |
| MT636065 | 2016 | Argentina | Asian | Human | Complete |  |
| MK028557 | 2017 | Thailand | Asian | Mosquito | Partial |  |
| MK028857 | 2015 | Puerto Rico | Asian | Human | Complete | PacBio; Illumina |
| MK028858 | 2015 | Panama | Asian | Human | Complete | PacBio; Illumina |
| MK028859 | 2015 | Panama | Asian | Human | Complete | PacBio; Illumina |
| MK028860 | 1984 | Senegal | West African | Mosquito | Complete | PacBio; Illumina |
| MK028861 | 2015 | Panama | Asian | Human | Complete | PacBio; Illumina |
| KU729218 | 2015 | Brazil | Asian | Human | Complete | Ion and 454 |
| MK028862 | 2010 | Cambodia | Asian | Human | Partial | PacBio; Illumina |
| MH675619 | 2016 | Puerto Rico | Asian | Human | Complete | Sanger dideoxy sequencing |
| MH675620 | 2016 | Puerto Rico | Asian | Human | Complete | Sanger dideoxy sequencing |
| MH675621 | 2016 | Puerto Rico | Asian | Human | Complete | Sanger dideoxy sequencing |
| MH675622 | 2016 | Puerto Rico | Asian | Human | Complete | Sanger dideoxy sequencing |
| MH675623 | 2016 | Puerto Rico | Asian | Human | Complete | Sanger dideoxy sequencing |
| MH675624 | 2016 | Puerto Rico | Asian | Human | Complete | Sanger dideoxy sequencing |
| MH675625 | 2016 | Puerto Rico | Asian | Human | Complete | Sanger dideoxy sequencing |
| MH675626 | 2016 | Puerto Rico | Asian | Human | Complete | Sanger dideoxy sequencing |
| MH675627 | 2016 | Puerto Rico | Asian | Human | Complete | Sanger dideoxy sequencing |
| MH675628 | 2016 | Puerto Rico | Asian | Human | Complete | Sanger dideoxy sequencing |
| MH675629 | 2016 | Puerto Rico | Asian | Human | Complete | Sanger dideoxy sequencing |
| MH675630 | 2016 | USA | Asian | Human | Complete | Sanger dideoxy sequencing |
| MT671183 | 2020 | Mexico | Asian | Animal | Partial | Sanger dideoxy sequencing |
| MT671184 | 2020 | Mexico | Asian | Animal | Partial | Sanger dideoxy sequencing |
| MK049245 | 2016 | Colombia | Asian | Human | Complete | Oxford Nanopore MinIon |
| MK049246 | 2016 | Colombia | Asian | Human | Complete | Oxford Nanopore MinIon |
| MK049247 | 2016 | Colombia | Asian | Mosquito | Complete | Oxford Nanopore MinIon |
| MK049248 | 2016 | Colombia | Asian | Human | Complete | Oxford Nanopore MinIon |
| MK049249 | 2016 | Colombia | Asian | Human | Complete | Oxford Nanopore MinIon |
| KU740199 | 2016 | China | Asian | Human | Partial | IonTorrent |
| MK049250 | 2016 | Colombia | Asian | Human | Partial | Oxford Nanopore MinIon |
| MK049251 | 2016 | Colombia | Asian | Human | Partial | Oxford Nanopore MinIon |
| MK049252 | 2016 | Colombia | Asian | Human | Complete | Oxford Nanopore MinIon |
| MK050090 | 2016 | Spain | Asian | Human | Partial | Sanger dideoxy sequencing |
| KX162585 | 2015 | Brazil | Asian | Animal | Partial | Sanger dideoxy sequencing |
| KX162586 | 2015 | Brazil | Asian | Animal | Partial | Sanger dideoxy sequencing |
| MN124090 | 2015 | Panama | Asian | Human | Complete | Illumina |
| MN124091 | 2015 | Panama | Asian | Human | Complete | Illumina |
| MK105975 | 2016 | Uganda | East African | Laboratory | Complete | Illumina |
| KY631492 | 2016 | Brazil | Asian | Human | Complete |  |
| KY631493 | 2015 | Mexico | Asian | Human | Complete | Illumina |
| KY631494 | 2015 | Mexico | Asian | Human | Complete | Illumina |
| KY648934 | 2016 | Mexico | Asian | Mosquito | Complete | Illumina |
| MG742364 | 2017 | Portugal | Asian | Human | Partial | Sanger dideoxy sequencing |
| MG751804 | 2016 | China | Asian | Human | Partial |  |
| MG751805 | 2016 | China | Asian | Human | Partial |  |
| MG770183 | 2017 | Brazil | Asian | Animal | Partial | Illumina |
| MG770184 | 2017 | Brazil | Asian | Animal | Partial | Illumina |
| KU752544 | 2015 | Portugal | Asian | Human | Partial | Sanger dideoxy sequencing |
| MG770185 | 2017 | Brazil | Asian | Animal | Partial | Illumina |
| MG770186 | 2017 | Brazil | Asian | Animal | Partial | Illumina |
| MG807646 | 2016 | Thailand | Asian | Human | Complete | IonTorrent |
| MG807647 | 2017 | Thailand | Asian | Human | Complete | IonTorrent |
| MG808108 | 2016 | Malaysia | Asian | Mosquito | Partial |  |
| MG808109 | 2016 | Malaysia | Asian | Mosquito | Partial |  |
| MG808110 | 2016 | Malaysia | Asian | Mosquito | Partial |  |
| MG808111 | 2016 | Malaysia | Asian | Mosquito | Partial |  |
| KU752545 | 2015 | Portugal | Asian | Human | Partial | Sanger dideoxy sequencing |
| MG808112 | 2016 | Malaysia | Asian | Mosquito | Partial |  |
| MG808113 | 2016 | Malaysia | Asian | Mosquito | Partial |  |
| MG808114 | 2016 | Malaysia | Asian | Mosquito | Partial |  |
| MG808115 | 2016 | Malaysia | Asian | Mosquito | Partial |  |
| MG808116 | 2016 | Malaysia | Asian | Mosquito | Partial |  |
| MG808117 | 2016 | Malaysia | Asian | Mosquito | Partial |  |
| MG808118 | 2016 | Malaysia | Asian | Mosquito | Partial |  |
| MG808119 | 2016 | Malaysia | Asian | Mosquito | Partial |  |
| MG808120 | 2016 | Malaysia | Asian | Mosquito | Partial |  |
| MG808121 | 2016 | Malaysia | Asian | Mosquito | Partial |  |
| KU758868 | 2016 | French Guiana | Asian | Human | Partial | Sanger dideoxy sequencing |
| MG808122 | 2016 | Malaysia | Asian | Mosquito | Partial |  |
| MG808123 | 2016 | Malaysia | Asian | Mosquito | Partial |  |
| MG808124 | 2016 | Malaysia | Asian | Mosquito | Partial |  |
| MG808125 | 2017 | Malaysia | Asian | Mosquito | Partial |  |
| MG808126 | 2017 | Malaysia | Asian | Mosquito | Partial |  |
| MG808127 | 2017 | Malaysia | Asian | Mosquito | Partial |  |
| MG808128 | 2017 | Malaysia | Asian | Mosquito | Partial |  |
| MG808129 | 2016 | Malaysia | Asian | Mosquito | Partial |  |
| MG808130 | 2016 | Malaysia | Asian | Mosquito | Partial |  |
| MG808131 | 2016 | Malaysia | Asian | Mosquito | Partial |  |
| KU758869 | 2016 | French Guiana | Asian | Human | Partial | Sanger dideoxy sequencing |
| MG808132 | 2016 | Malaysia | Asian | Mosquito | Partial |  |
| MG808133 | 2016 | Malaysia | Asian | Mosquito | Partial |  |
| MG808134 | 2016 | Malaysia | Asian | Mosquito | Partial |  |
| MG808135 | 2016 | Malaysia | Asian | Mosquito | Partial |  |
| MG808136 | 2016 | Malaysia | Asian | Mosquito | Partial |  |
| MG808137 | 2016 | Malaysia | Asian | Mosquito | Partial |  |
| MH718911 | 2015 | Russia | Asian | Human | Partial | Sanger dideoxy sequencing |
| MH718912 | 2015 | Russia | Asian | Human | Partial | Sanger dideoxy sequencing |
| MH718913 | 2013 | Russia | Asian | Human | Partial | Sanger dideoxy sequencing |
| MH718914 | 2012 | Russia | Asian | Human | Partial |  |
| KU758870 | 2015 | French Guiana | Asian | Human | Partial | Sanger dideoxy sequencing |
| MH718916 | 2013 | Russia | Asian | Human | Partial | Sanger dideoxy sequencing |
| MH718915 | 2013 | Russia | Asian | Human | Partial | Sanger dideoxy sequencing |
| MH718917 | 2013 | Russia | Asian | Human | Partial | Sanger dideoxy sequencing |
| MH718918 | 2013 | Russia | Asian | Human | Partial | Sanger dideoxy sequencing |
| MH718919 | 2012 | Russia | Asian | Human | Partial | Sanger dideoxy sequencing |
| MH718920 | 2013 | Russia | Asian | Human | Partial | Sanger dideoxy sequencing |
| MH718921 | 2013 | Russia | Asian | Human | Partial | Sanger dideoxy sequencing |
| MH718922 | 2012 | Russia | Asian | Human | Partial | Sanger dideoxy sequencing |
| KX173840 | 2016 | Brazil | Asian | Human | Partial | Sanger dideoxy sequencing |
| MH718923 | 2012 | Russia | Asian | Human | Partial | Sanger dideoxy sequencing |
| KU758871 | 2015 | French Guiana | Asian | Human | Partial | Sanger dideoxy sequencing |
| KX173841 | 2016 | Brazil | Asian | Human | Partial | Sanger dideoxy sequencing |
| KX173842 | 2016 | Brazil | Asian | Human | Partial | Sanger dideoxy sequencing |
| KX173843 | 2016 | Brazil | Asian | Human | Partial | Sanger dideoxy sequencing |
| KX173844 | 2016 | Brazil | Asian | Human | Partial | Sanger dideoxy sequencing |
| KX185891 | 2016 | China | Asian | Human | Complete | 454 |
| KX197192 | 2015 | Brazil | Asian | Human | Complete | Illumina |
| KX197205 | 2015 | Brazil | Asian | Human | Complete | Illumina |
| KX198134 | 1984 | Senegal | West African | Mosquito | Complete | Illumina |
| KX198135 | 2016 | Panama | Asian | Human | Complete | Illumina |
| KX212103 | 2015 | Brazil | Asian | Human | Partial |  |
| KU758872 | 2016 | French Guiana | Asian | Human | Partial | Sanger dideoxy sequencing |
| KY693676 | 2016 | Honduras | Asian | Human | Complete | Illumina |
| KY693677 | 2016 | Honduras | Asian | Human | Complete | Illumina |
| KX216632 | 2015 | Solomon Islands | Asian | Human | Partial | Sanger dideoxy sequencing |
| KY693678 | 2016 | Peru | Asian | Human | Complete | Illumina |
| KX216633 | 2016 | Viet Nam | Asian | Human | Partial | Sanger dideoxy sequencing |
| KY693679 | 2016 | Peru | Asian | Human | Complete | Illumina |
| KX216634 | 2016 | Tonga | Asian | Human | Partial | Sanger dideoxy sequencing |
| KY693680 | 2016 | Venezuela | Asian | Human | Complete | Illumina |
| KX216635 | 2016 | Tonga | Asian | Human | Partial | Sanger dideoxy sequencing |
| KX216636 | 2016 | Samoa | Asian | Human | Partial | Sanger dideoxy sequencing |
| KU758873 | 2016 | French Guiana | Asian | Human | Partial | Sanger dideoxy sequencing |
| KX216637 | 2016 | Guyana | Asian | Human | Partial | Sanger dideoxy sequencing |
| KX216638 | 2015 | El Salvador | Asian | Human | Partial | Sanger dideoxy sequencing |
| KX216639 | 2016 | Samoa | Asian | Human | Partial | Sanger dideoxy sequencing |
| KX216640 | 2014 | Cook Islands | Asian | Human | Partial | Sanger dideoxy sequencing |
| MH763832 |  | Brazil | Asian | Human | Complete | Illumina; Oxford Nanopore |
| MH763833 |  | Brazil | Asian | Human | Complete | Illumina; Oxford Nanopore |
| KY765303 | 2016 | Spain | Asian | Human | Partial | Sanger dideoxy sequencing |
| KY765304 | 2016 | Spain | Asian | Human | Partial | Sanger dideoxy sequencing |
| KY765305 | 2016 | Spain | Asian | Human | Partial | Sanger dideoxy sequencing |
| KY765306 | 2016 | Spain | Asian | Human | Partial | Sanger dideoxy sequencing |
| KU758874 | 2016 | French Guiana | Asian | Human | Partial | Sanger dideoxy sequencing |
| KY765307 | 2016 | Spain | Asian | Human | Partial | Sanger dideoxy sequencing |
| KY765308 | 2016 | Spain | Asian | Human | Partial | Sanger dideoxy sequencing |
| KY765309 | 2016 | Spain | Asian | Human | Partial | Sanger dideoxy sequencing |
| KY765317 | 2016 | Nicaragua | Asian | Human | Complete | Illumina |
| KY765318 | 2016 | Nicaragua | Asian | Human | Complete | Illumina |
| KY765319 | 2016 | Nicaragua | Asian | Human | Complete | Illumina |
| KY765320 | 2016 | Nicaragua | Asian | Human | Complete | Illumina |
| KY765321 | 2016 | Nicaragua | Asian | Human | Complete | Illumina |
| KY765322 | 2016 | Nicaragua | Asian | Human | Complete | Illumina |
| KY765323 | 2016 | Nicaragua | Asian | Human | Complete | Illumina |
| KU758875 | 2016 | French Guiana | Asian | Human | Partial | Sanger dideoxy sequencing |
| KY765324 | 2016 | Nicaragua | Asian | Human | Complete | Illumina |
| KY765325 | 2016 | Nicaragua | Asian | Human | Complete | Illumina |
| MW008559 | 2016 | Trinidad and Tobago | Asian | Human | Complete | Illumina |
| KY765326 | 2016 | Nicaragua | Asian | Human | Complete | Illumina |
| MG827392 | 2013 | French Polynesia | Asian | Human | Complete | Illumina |
| KY765327 | 2016 | Nicaragua | Asian | Human | Complete | Illumina |
| KY766069 | 2013 | French Polynesia | Asian | Human | Complete | IonTorrent |
| MW012427 | 2016 | Trinidad and Tobago | Asian | Human | Complete | Sanger dideoxy sequencing |
| MW012428 | 2016 | Trinidad and Tobago | Asian | Human | Complete | Sanger dideoxy sequencing |
| KY785409 | 2016 | Brazil | Asian | Human | Partial | Illumina; Swift LC |
| KU758876 | 2015 | Suriname | Asian | Human | Partial | Sanger dideoxy sequencing |
| KY785410 | 2016 | Brazil | Asian | Human | Complete | Illumina; Swift LC |
| KY785411 | 2016 | Brazil | Asian | Human | Partial | Illumina; Swift LC |
| KY785412 | 2016 | USA | Asian | Human | Complete | Illumina; Swift LC |
| KY785413 | 2016 | Dominican Republic | Asian | Human | Complete | Illumina; Swift LC |
| KY785414 | 2016 | Honduras | Asian | Human | Complete | Illumina; Swift LC |
| KY785415 | 2016 | Dominican Republic | Asian | Human | Complete | Illumina; Swift LC |
| KY785416 | 2016 | Honduras | Asian | Human | Partial | Illumina; Swift LC |
| KY785417 | 2016 | Colombia | Asian | Human | Complete | Illumina; Swift LC |
| KY785418 | 2016 | Honduras | Asian | Human | Complete | Illumina; Swift LC |
| KY785419 | 2016 | Jamaica | Asian | Human | Complete | Illumina; Swift LC |
| KU758877 | 2015 | French Guiana | Asian | Human | Complete | Sanger dideoxy sequencing |
| KY785420 | 2016 | Dominican Republic | Asian | Human | Complete | Illumina; Swift LC |
| KY785421 | 2016 | USA | Asian | Human | Partial | Illumina; Swift LC |
| KY785422 | 2016 | USA | Asian | Mosquito | Complete | Illumina; Swift LC |
| KY785423 | 2016 | Dominican Republic | Asian | Human | Complete | Illumina; Swift LC |
| KY785424 | 2016 | Jamaica | Asian | Human | Complete | Illumina; Swift LC |
| KY785425 | 2016 | Dominican Republic | Asian | Human | Partial | Illumina; Swift LC |
| KY785426 | 2016 | Brazil | Asian | Human | Complete | Illumina; Swift LC |
| KY785427 | 2016 | Brazil | Asian | Human | Complete | Illumina; Swift LC |
| KY785428 | 2016 | Dominican Republic | Asian | Human | Partial | Illumina; Swift LC |
| KY785429 | 2016 | Brazil | Asian | Human | Complete | Illumina; Swift LC |
| KU758878 | 2016 | Thailand | Asian | Human | Partial | Sanger dideoxy sequencing |
| MW015936 | 2006 | Thailand | Asian | Human | Complete | Sanger dideoxy sequencing |
| KY785430 | 2016 | Jamaica | Asian | Human | Complete | Illumina; Swift LC |
| KY785431 | 2016 | Honduras | Asian | Human | Partial | Illumina; Swift LC |
| KY785432 | 2016 | Jamaica | Asian | Human | Complete | Illumina; Swift LC |
| KY785433 | 2016 | Brazil | Asian | Human | Complete | Illumina; Swift LC |
| KY785434 | 2016 | Dominican Republic | Asian | Human | Partial | Illumina; Swift LC |
| KY785435 | 2016 | Dominican Republic | Asian | Human | Complete | Illumina; Swift LC |
| KY785436 | 2016 | Brazil | Asian | Human | Partial | Illumina; Swift LC |
| KY785437 | 2016 | Brazil | Asian | Human | Complete | Illumina; Swift LC |
| MK216687 | 2016 | Brazil | Asian | Human | Complete |  |
| KU761560 | 2016 | China | Asian | Human | Complete | IonTorrent |
| MK216688 | 2016 | Brazil | Asian | Human | Complete |  |
| KY785438 | 2016 | Jamaica | Asian | Human | Partial | Illumina; Swift LC |
| MK216689 | 2016 | Brazil | Asian | Human | Partial |  |
| KY785439 | 2016 | Brazil | Asian | Human | Complete | Illumina; Swift LC |
| MK216690 | 2016 | Brazil | Asian | Human | Complete |  |
| KY785440 | 2016 | USA | Asian | Human | Partial | Illumina; Swift LC |
| MK216691 | 2016 | Brazil | Asian | Human | Partial |  |
| KY785441 | 2016 | Dominican Republic | Asian | Human | Complete | Illumina; Swift LC |
| MK216692 | 2016 | Brazil | Asian | Human | Complete |  |
| KY785442 | 2016 | Honduras | Asian | Human | Complete | Illumina; Swift LC |
| KU761561 | 2016 | China | Asian | Human | Complete | IonTorrent |
| MK216693 | 2016 | Brazil | Asian | Human | Partial |  |
| KY785443 | 2016 | USA | Asian | Human | Partial | Illumina; Swift LC |
| MK216694 | 2016 | Brazil | Asian | Human | Partial |  |
| KY785444 | 2016 | Honduras | Asian | Human | Complete | Illumina; Swift LC |
| MK216695 | 2016 | Brazil | Asian | Human | Complete |  |
| KY785445 | 2016 | USA | Asian | Human | Complete | Illumina; Swift LC |
| MK216696 | 2016 | Brazil | Asian | Human | Complete |  |
| KY785446 | 2016 | Brazil | Asian | Human | Partial | Illumina; Swift LC |
| KY785447 | 2016 | Dominican Republic | Asian | Human | Complete | Illumina; Swift LC |
| MK216697 | 2016 | Brazil | Asian | Human | Complete |  |
| KU761564 | 2016 | China | Asian | Human | Complete | Sanger dideoxy sequencing |
| MK216698 | 2016 | Brazil | Asian | Human | Complete |  |
| KY785448 | 2016 | Honduras | Asian | Human | Complete | Illumina; Swift LC |
| KY785449 | 2016 | Dominican Republic | Asian | Human | Complete | Illumina; Swift LC |
| MK216699 | 2016 | Brazil | Asian | Human | Complete |  |
| KY785450 | 2016 | Brazil | Asian | Human | Complete | Illumina; Swift LC |
| MK216700 | 2017 | Brazil | Asian | Human | Partial |  |
| MK216701 | 2017 | Brazil | Asian | Human | Partial |  |
| KY785451 | 2016 | Martinique | Asian | Human | Complete | Illumina; Swift LC |
| MK216702 | 2016 | Brazil | Asian | Human | Partial |  |
| KY785452 | 2016 | Honduras | Asian | Human | Complete | Illumina; Swift LC |
| KU820897 | 2015 | Colombia | Asian | Human | Complete | Illumina |
| KY785453 | 2016 | Dominican Republic | Asian | Human | Complete | Illumina; Swift LC |
| MK216703 | 2016 | Brazil | Asian | Human | Partial |  |
| KY785454 | 2016 | El Salvador | Asian | Human | Partial | Illumina; Swift LC |
| MK216704 | 2016 | Brazil | Asian | Human | Partial |  |
| MK216705 | 2016 | Brazil | Asian | Human | Partial |  |
| KY785455 | 2016 | Brazil | Asian | Human | Complete | Illumina; Swift LC |
| KY785456 | 2016 | Brazil | Asian | Human | Complete | Illumina; Swift LC |
| MK216706 | 2016 | Brazil | Asian | Human | Partial |  |
| KY785457 | 2016 | USA | Asian | Human | Complete | Illumina; Swift LC |
| MK216707 | 2016 | Brazil | Asian | Human | Partial |  |
| KU820898 | 2016 | China | Asian | Human | Complete | Sanger dideoxy sequencing |
| KY785458 | 2016 | Honduras | Asian | Human | Partial | Illumina; Swift LC |
| MK216708 | 2016 | Brazil | Asian | Human | Partial |  |
| KY785459 | 2016 | USA | Asian | Human | Complete | Illumina; Swift LC |
| MK216709 | 2016 | Brazil | Asian | Human | Partial |  |
| KY785460 | 2016 | Dominican Republic | Asian | Human | Partial | Illumina; Swift LC |
| MK216710 | 2016 | Brazil | Asian | Human | Complete |  |
| KY785461 | 2016 | Honduras | Asian | Human | Complete | Illumina; Swift LC |
| MK216711 | 2016 | Brazil | Asian | Human | Partial |  |
| KY785462 | 2016 | Puerto Rico | Asian | Human | Complete | Illumina; Swift LC |
| MK216712 | 2016 | Brazil | Asian | Human | Partial |  |
| KU820899 | 2016 | China | Asian | Human | Complete | Sanger dideoxy sequencing; IonTorrent |
| KY785463 | 2016 | Dominican Republic | Asian | Human | Complete | Illumina; Swift LC |
| MK216713 | 2016 | Brazil | Asian | Human | Complete |  |
| KY785464 | 2016 | Puerto Rico | Asian | Human | Complete | Illumina; Swift LC |
| KY785465 | 2016 | Dominican Republic | Asian | Human | Complete | Illumina; Swift LC |
| MK216714 | 2016 | Brazil | Asian | Human | Partial |  |
| KY785466 | 2016 | Colombia | Asian | Human | Complete | Illumina; Swift LC |
| MK216715 | 2016 | Brazil | Asian | Human | Partial |  |
| KY785467 | 2016 | Brazil | Asian | Human | Partial | Illumina; Swift LC |
| MK216716 | 2016 | Brazil | Asian | Human | Partial |  |
| KY785468 | 2016 | USA | Asian | Mosquito | Complete | Illumina; Swift LC |
| KU844090 | 2016 | Russia | Asian | Human | Partial | Sanger dideoxy sequencing |
| MK216717 | 2016 | Brazil | Asian | Human | Partial |  |
| KY785469 | 2016 | Colombia | Asian | Human | Complete | Illumina; Swift LC |
| MK216718 | 2016 | Brazil | Asian | Human | Partial |  |
| KY785470 | 2016 | Dominican Republic | Asian | Human | Complete | Illumina; Swift LC |
| MK216719 | 2016 | Brazil | Asian | Human | Partial |  |
| KY785471 | 2016 | Honduras | Asian | Human | Partial | Illumina; Swift LC |
| MK216720 | 2016 | Brazil | Asian | Human | Partial |  |
| KY785472 | 2016 | USA | Asian | Mosquito | Complete | Illumina; Swift LC |
| MK216721 | 2016 | Brazil | Asian | Human | Partial |  |
| KY785473 | 2016 | Dominican Republic | Asian | Human | Partial | Illumina; Swift LC |
| KU853012 | 2016 | Italy | Asian | Human | Complete | Sanger dideoxy sequencing |
| MK216722 | 2016 | Brazil | Asian | Human | Partial |  |
| MK216723 | 2016 | Brazil | Asian | Human | Partial |  |
| KY785474 | 2016 | USA | Asian | Human | Complete | Illumina; Swift LC |
| KY785475 | 2016 | Dominican Republic | Asian | Human | Complete | Illumina; Swift LC |
| MK216724 | 2016 | Brazil | Asian | Human | Partial |  |
| KY785476 | 2016 | Dominican Republic | Asian | Human | Complete | Illumina; Swift LC |
| MK216725 | 2016 | Brazil | Asian | Human | Partial |  |
| MK216726 | 2016 | Brazil | Asian | Human | Partial |  |
| KY785477 | 2016 | Colombia | Asian | Human | Partial | Illumina; Swift LC |
| MK216727 | 2016 | Brazil | Asian | Human | Complete |  |
| KU853013 | 2016 | Italy | Asian | Human | Complete | Sanger dideoxy sequencing |
| KY785478 | 2016 | Dominican Republic | Asian | Human | Partial | Illumina; Swift LC |
| MK216728 | 2016 | Brazil | Asian | Human | Partial |  |
| KY785479 | 2016 | Brazil | Asian | Human | Complete | Illumina; Swift LC |
| MK216729 | 2015 | Brazil | Asian | Human | Partial |  |
| KY785480 | 2016 | Brazil | Asian | Human | Partial | Illumina; Swift LC |
| MK216730 | 2016 | Brazil | Asian | Human | Partial |  |
| KY785481 | 2016 | Puerto Rico | Asian | Human | Partial | Illumina; Swift LC |
| MK216731 | 2016 | Brazil | Asian | Human | Partial |  |
| KY785482 | 2016 | Haiti | Asian | Human | Partial | Illumina; Swift LC |
| MK216732 | 2016 | Brazil | Asian | Human | Partial |  |
| KU866423 | 2016 | China | Asian | Human | Complete | Sanger dideoxy sequencing |
| KY785483 | 2016 | Dominican Republic | Asian | Human | Partial | Illumina; Swift LC |
| MK216733 | 2016 | Brazil | Asian | Human | Partial |  |
| KX247632 | 2015 | Mexico | Asian | Mosquito | Complete | Illumina |
| KY785484 | 2016 | Dominican Republic | Asian | Human | Complete | Illumina; Swift LC |
| MK216734 | 2017 | Brazil | Asian | Human | Partial |  |
| KY785485 | 2016 | Brazil | Asian | Human | Partial | Illumina; Swift LC |
| MK216735 | 2017 | Brazil | Asian | Human | Partial |  |
| KX247638 | 2016 | Russia | Asian | Human | Partial | Sanger dideoxy sequencing |
| KX247646 | 2016 | Colombia | Asian | Human | Complete | Sanger dideoxy sequencing |
| MK216736 | 2017 | Brazil | Asian | Human | Partial |  |
| KU867812 | 2016 | China | Asian | Human | Partial | Sanger dideoxy sequencing |
| KX253994 | 2016 | Germany | Asian | Human | Partial | Sanger dideoxy sequencing |
| MK216737 | 2017 | Brazil | Asian | Human | Partial |  |
| MK216738 | 2017 | Brazil | Asian | Human | Partial |  |
| KX253995 | 2016 | Germany | Asian | Human | Partial | Sanger dideoxy sequencing |
| KX253996 | 2016 | China | Asian | Human | Complete | IonTorrent; 5' RACE and 3' RACE (Sanger |
| MK216739 | 2017 | Brazil | Asian | Human | Partial |  |
| MK216740 | 2017 | Brazil | Asian | Human | Partial |  |
| MK216741 | 2017 | Brazil | Asian | Human | Complete |  |
| MK216742 | 2017 | Brazil | Asian | Human | Complete |  |
| MK216743 | 2017 | Brazil | Asian | Human | Complete |  |
| KU870645 | 2016 | USA | Asian | Human | Complete |  |
| MK216744 | 2017 | Brazil | Asian | Human | Partial |  |
| MK216745 | 2015 | Brazil | Asian | Human | Complete |  |
| MK216746 | 2016 | Brazil | Asian | Human | Partial |  |
| MK216747 | 2015 | Brazil | Asian | Human | Partial |  |
| MK216748 | 2015 | Brazil | Asian | Human | Partial |  |
| MG877675 | 2016 | Colombia | Asian | Human | Partial |  |
| KX261851 | 2016 | Colombia | Asian | Human | Partial | Sanger dideoxy sequencing |
| KX261852 | 2016 | Colombia | Asian | Human | Partial | Sanger dideoxy sequencing |
| KX261853 | 2016 | Colombia | Asian | Human | Partial | Sanger dideoxy sequencing |
| KX261854 | 2016 | Colombia | Asian | Human | Partial | Sanger dideoxy sequencing |
| KU872850 | 2016 | Russia | Asian | Human | Partial | Sanger dideoxy sequencing |
| MK237993 | 2017 | Thailand | Asian | Human | Partial | Sanger dideoxy sequencing |
| KX261855 | 2016 | Colombia | Asian | Human | Partial | Sanger dideoxy sequencing |
| MK237994 | 2017 | Thailand | Asian | Human | Partial | Sanger dideoxy sequencing |
| KX262887 | 2016 | Honduras | Asian | Human | Complete | IonTorrent |
| MK237995 | 2017 | Thailand | Asian | Human | Partial | Sanger dideoxy sequencing |
| MK237996 | 2017 | Thailand | Asian | Human | Partial | Sanger dideoxy sequencing |
| MK237997 | 2017 | Thailand | Asian | Human | Partial | Sanger dideoxy sequencing |
| KY817930 | 2016 | Brazil | Asian | Human | Complete | MinIon R9.x, Oxford Nanopore Technologies |
| MK237998 | 2017 | Thailand | Asian | Human | Partial | Sanger dideoxy sequencing |
| MK237999 | 2017 | Thailand | Asian | Human | Partial | Sanger dideoxy sequencing |
| HQ234498 | 1947 | Uganda | East African | Animal | Partial |  |
| MK238035 | 2018 | India | Asian | Human | Complete |  |
| MK238036 | 2018 | India | Asian | Human | Partial |  |
| MK238037 | 2018 | India | Asian | Human | Complete |  |
| MK238038 | 2018 | India | Asian | Human | Complete |  |
| MK238039 | 2018 | India | Asian | Human | Partial |  |
| MK241415 | 2015 | Cape Verde | Asian | Human | Complete | Illumina |
| MK241416 | 2015 | Cape Verde | Asian | Human | Complete | Illumina |
| KX266255 | 2016 | China | Asian | Human | Complete | Sanger dideoxy sequencing |
| MK241417 | 2016 | Cape Verde | Asian | Human | Complete | Illumina |
| KX269878 | 2016 | Italy | Asian | Human | Complete |  |
| HQ234499 | 1966 | Malaysia | Asian | Mosquito | Partial |  |
| KX280026 | 2015 | Brazil | Asian | Human | Complete | Sanger dideoxy sequencing |
| KX346600 | 2014 | Bangladesh | Asian | Human | Partial | Sanger dideoxy sequencing |
| MW122373 | 2016 | Puerto Rico | Asian | Human | Complete | Illumina |
| MW122374 | 2016 | Puerto Rico | Asian | Human | Complete | Illumina |
| MW122375 | 2016 | Puerto Rico | Asian | Human | Complete | Illumina |
| MW122376 | 2016 | Puerto Rico | Asian | Human | Complete | Illumina |
| MW122377 | 2016 | Puerto Rico | Asian | Human | Complete | Illumina |
| MW122378 | 2016 | Puerto Rico | Asian | Human | Complete | Illumina |
| MW122379 | 2016 | Puerto Rico | Asian | Human | Complete | Illumina |
| MW122380 | 2016 | Puerto Rico | Asian | Human | Complete | Illumina |
| HQ234500 | 1968 | Nigeria | West African | Human | Partial |  |
| MW122381 | 2016 | Puerto Rico | Asian | Human | Complete | Illumina |
| MW122382 | 2016 | Puerto Rico | Asian | Human | Complete | Illumina |
| MW122383 | 2016 | Puerto Rico | Asian | Human | Complete | Illumina |
| MW122384 | 2016 | Puerto Rico | Asian | Human | Complete | Illumina |
| MW122385 | 2016 | Puerto Rico | Asian | Human | Complete | Illumina |
| MW122386 | 2016 | Puerto Rico | Asian | Human | Complete | Illumina |
| MW122387 | 2016 | Puerto Rico | Asian | Human | Complete | Illumina |
| MW122388 | 2016 | Puerto Rico | Asian | Human | Complete | Illumina |
| MW122389 | 2016 | Puerto Rico | Asian | Human | Complete | Illumina |
| MW122390 | 2016 | Puerto Rico | Asian | Human | Complete | Illumina |
| HQ234501 | 1984 | Senegal | West African | Mosquito | Partial |  |
| MW122391 | 2016 | Puerto Rico | Asian | Human | Complete | Illumina |
| MW122392 | 2016 | Puerto Rico | Asian | Human | Complete | Illumina |
| MW122393 | 2016 | Puerto Rico | Asian | Human | Complete | Illumina |
| MW122394 | 2016 | Puerto Rico | Asian | Human | Complete | Illumina |
| MW122395 | 2016 | Puerto Rico | Asian | Human | Complete | Illumina |
| MW122396 | 2016 | Puerto Rico | Asian | Human | Complete | Illumina |
| MW122397 | 2016 | Puerto Rico | Asian | Human | Complete | Illumina |
| MW122398 | 2016 | Puerto Rico | Asian | Human | Complete | Illumina |
| MW122399 | 2016 | Puerto Rico | Asian | Human | Complete | Illumina |
| MW122400 | 2016 | Puerto Rico | Asian | Human | Complete | Illumina |
| AY632535 |  | Uganda | East African | Animal | Complete |  |
| MW122401 | 2016 | Puerto Rico | Asian | Human | Complete | Illumina |
| MW122402 | 2016 | Puerto Rico | Asian | Human | Complete | Illumina |
| MW122403 | 2016 | Puerto Rico | Asian | Human | Complete | Illumina |
| MW122404 | 2016 | Puerto Rico | Asian | Human | Complete | Illumina |
| MW122405 | 2016 | Puerto Rico | Asian | Human | Complete | Illumina |
| MW122406 | 2016 | Puerto Rico | Asian | Human | Complete | Illumina |
| MW122407 | 2016 | Puerto Rico | Asian | Human | Complete | Illumina |
| MW122408 | 2016 | Puerto Rico | Asian | Human | Complete | Illumina |
| MW122409 | 2016 | Puerto Rico | Asian | Human | Complete | Illumina |
| MW122410 | 2016 | Puerto Rico | Asian | Human | Complete | Illumina |
| AB908162 | 2014 | French Polynesia | Asian | Human | Partial |  |
| MW122411 | 2016 | Puerto Rico | Asian | Human | Complete | Illumina |
| MW122412 | 2016 | Puerto Rico | Asian | Human | Complete | Illumina |
| MW122413 | 2016 | Puerto Rico | Asian | Human | Complete | Illumina |
| MW122414 | 2016 | Puerto Rico | Asian | Human | Complete | Illumina |
| MW122415 | 2017 | Puerto Rico | Asian | Human | Complete | Illumina |
| MW122417 | 2016 | Puerto Rico | Asian | Human | Complete | Illumina |
| MW122418 | 2016 | Puerto Rico | Asian | Human | Complete | Illumina |
| MW122419 | 2016 | Puerto Rico | Asian | Human | Complete | Illumina |
| MW122420 | 2016 | Puerto Rico | Asian | Human | Complete | Illumina |
| MW122421 | 2016 | Puerto Rico | Asian | Human | Complete | Illumina |
| MW122422 | 2016 | Puerto Rico | Asian | Human | Complete | Illumina |
| MW122423 | 2016 | Puerto Rico | Asian | Human | Complete | Illumina |
| MW122424 | 2016 | Puerto Rico | Asian | Human | Complete | Illumina |
| MW122425 | 2016 | Puerto Rico | Asian | Human | Complete | Illumina |
| MW122426 | 2016 | Puerto Rico | Asian | Human | Complete | Illumina |
| MW122427 | 2016 | Puerto Rico | Asian | Human | Complete | Illumina |
| MW122428 | 2016 | Puerto Rico | Asian | Human | Complete | Illumina |
| MW122429 | 2016 | Puerto Rico | Asian | Human | Complete | Illumina |
| MW122430 | 2016 | Puerto Rico | Asian | Human | Complete | Illumina |
| MW122431 | 2016 | Puerto Rico | Asian | Human | Complete | Illumina |
| KX358623 | 2016 | Venezuela | Asian | Human | Partial | Sanger dideoxy sequencing |
| KX369547 | 2013 | French Polynesia | Asian | Human | Complete | Illumina |
| MH882527 | 2016 | Brazil | Asian | Human | Complete | Illumina |
| MH882528 | 2016 | Brazil | Asian | Human | Complete | Illumina |
| MH882530 | 2016 | Brazil | Asian | Human | Complete | Illumina |
| MH882531 | 2016 | Brazil | Asian | Human | Complete | Illumina |
| MH882532 | 2016 | Brazil | Asian | Human | Complete | Illumina |
| MH882533 | 2016 | Brazil | Asian | Human | Complete | Illumina |
| MH882534 | 2016 | Brazil | Asian | Human | Complete | Illumina |
| MH882535 | 2016 | Brazil | Asian | Human | Complete | Illumina |
| MH882536 | 2016 | Brazil | Asian | Human | Complete | Illumina |
| MH882529 | 2016 | Brazil | Asian | Human | Complete | Illumina |
| MH882537 | 2016 | Brazil | Asian | Human | Partial | Illumina |
| MH882538 | 2016 | Brazil | Asian | Human | Complete | Illumina |
| MH882539 | 2016 | Brazil | Asian | Human | Complete | Illumina |
| MH882540 | 2016 | Brazil | Asian | Human | Complete | Illumina |
| KX377120 | 2016 | Spain | Asian | Human | Partial | Sanger dideoxy sequencing |
| MH882541 | 2016 | Brazil | Asian | Human | Complete | Illumina |
| MH882542 | 2016 | Brazil | Asian | Human | Complete | Illumina |
| KX377335 | 1947 | Uganda | East African | Animal | Complete | Sanger dideoxy sequencing |
| MH882543 | 2016 | Brazil | Asian | Human | Complete | Illumina |
| KX377336 | 1966 | Malaysia | Asian | Mosquito | Complete | Sanger dideoxy sequencing |
| MH882544 | 2016 | Brazil | Asian | Human | Complete | Illumina |
| KX377337 | 2015 | Puerto Rico | Asian | Human | Complete | Sanger dideoxy sequencing |
| MH882545 | 2016 | Brazil | Asian | Human | Complete | Illumina |
| MH882546 | 2016 | Brazil | Asian | Human | Complete | Illumina |
| MH882547 | 2016 | Brazil | Asian | Human | Complete | Illumina |
| MH882548 | 2016 | Brazil | Asian | Human | Complete | Illumina |
| MH882549 | 2016 | Brazil | Asian | Human | Partial | Illumina |
| KU886298 | 2016 | France | Asian | Human | Partial | Sanger dideoxy sequencing |
| KX380262 | 2016 | Mexico | Asian | Human | Partial | Sanger dideoxy sequencing |
| KX380263 | 2016 | Fiji | Asian | Human | Partial | Sanger dideoxy sequencing |
| MN170700 | 2018 | Brazil | Asian | Mosquito | Partial | Sanger dideoxy sequencing |
| MN170701 | 2018 | Brazil | Asian | Mosquito | Partial | Sanger dideoxy sequencing |
| MN170702 | 2018 | Brazil | Asian | Mosquito | Partial | Sanger dideoxy sequencing |
| MN170703 | 2018 | Brazil | Asian | Mosquito | Partial | Sanger dideoxy sequencing |
| MN170704 | 2018 | Brazil | Asian | Mosquito | Partial | Sanger dideoxy sequencing |
| MN170705 | 2018 | Brazil | Asian | Mosquito | Partial | Sanger dideoxy sequencing |
| MN170706 | 2018 | Brazil | Asian | Mosquito | Partial | Sanger dideoxy sequencing |
| MN170707 | 2018 | Brazil | Asian | Mosquito | Partial | Sanger dideoxy sequencing |
| KF258813 | 2012 | Indonesia | Asian | Human | Partial | Sanger dideoxy sequencing |
| MN170708 | 2018 | Brazil | Asian | Mosquito | Partial | Sanger dideoxy sequencing |
| MN170709 | 2018 | Brazil | Asian | Mosquito | Partial | Sanger dideoxy sequencing |
| MN170710 | 2018 | Brazil | Asian | Mosquito | Partial | Sanger dideoxy sequencing |
| MN170711 | 2018 | Brazil | Asian | Mosquito | Partial | Sanger dideoxy sequencing |
| MN170712 | 2018 | Brazil | Asian | Mosquito | Partial | Sanger dideoxy sequencing |
| MN170713 | 2018 | Brazil | Asian | Mosquito | Partial | Sanger dideoxy sequencing |
| MN170714 | 2018 | Brazil | Asian | Mosquito | Partial | Sanger dideoxy sequencing |
| MN170715 | 2018 | Brazil | Asian | Mosquito | Partial | Sanger dideoxy sequencing |
| MN171421 | 2015 | Puerto Rico | Asian | Human | Partial | Illumina |
| MN185324 | 2016 | Guadeloupe | Asian | Mosquito | Complete | IonTorrent |
| KF268948 | 1976 | Central African Republic | East African | Mosquito | Complete | Illumina |
| MN185325 | 2016 | Guadeloupe | Asian | Mosquito | Complete | IonTorrent |
| MN185326 | 2016 | French Guiana | Asian | Mosquito | Complete | IonTorrent |
| MN185327 | 2016 | Guadeloupe | Asian | Mosquito | Complete | IonTorrent |
| MN185328 | 2016 | French Guiana | Asian | Mosquito | Complete | IonTorrent |
| MN185329 | 2016 | Guadeloupe | Asian | Mosquito | Complete | IonTorrent |
| MN185330 | 2016 | Guadeloupe | Asian | Mosquito | Complete | IonTorrent |
| MN185331 | 2016 | Guadeloupe | Asian | Mosquito | Complete | IonTorrent |
| MN185332 | 2016 | Guadeloupe | Asian | Mosquito | Complete | IonTorrent |
| MN190155 | 2019 | China | Asian | Human | Complete |  |
| KX421193 | 1947 | Uganda | East African |  | Partial | Illumina |
| KF268949 |  | Central African Republic | East African | Mosquito | Complete | Illumina |
| KX421194 | 2016 | Nicaragua | Asian | Human | Complete | Illumina |
| KX421195 | 2016 | Nicaragua | Asian | Human | Complete | Illumina |
| MH900227 | 2016 | Mexico | Asian | Mosquito | Complete | Illumina |
| KY888678 | 2016 | China | Asian | Human | Partial | Sanger dideoxy sequencing |
| KY921911 | 2016 | Singapore | Asian | Human | Partial | Sanger dideoxy sequencing |
| KX443144 | 2016 | Brazil | Asian | Human | Partial | Sanger dideoxy sequencing |
| KX443145 | 2016 | Brazil | Asian | Human | Partial | Sanger dideoxy sequencing |
| KX446950 | 2016 | Mexico | Asian | Mosquito | Complete | Illumina |
| KX446951 | 2016 | Mexico | Asian | Mosquito | Complete | Illumina |
| KX447509 | 2013 | French Polynesia | Asian | Human | Complete | IonTorrent |
| KF268950 |  | Central African Republic | East African | Mosquito | Complete | Illumina |
| KX447511 | 2014 | French Polynesia | Asian | Human | Complete | IonTorrent |
| KX447512 | 2013 | French Polynesia | Asian | Human | Complete | IonTorrent |
| KX447513 | 2013 | French Polynesia | Asian | Human | Complete | IonTorrent |
| KX447514 | 2014 | French Polynesia | Asian | Human | Complete | IonTorrent |
| KX447515 | 2013 | French Polynesia | Asian | Human | Complete | IonTorrent |
| KX447516 | 2014 | French Polynesia | Asian | Human | Complete | IonTorrent |
| KX447517 | 2014 | French Polynesia | Asian | Human | Complete | IonTorrent |
| KX447518 | 2013 | French Polynesia | Asian | Human | Partial | IonTorrent |
| KX447519 | 2013 | French Polynesia | Asian | Human | Partial | IonTorrent |
| KF270886 | 2007 | Gabon | West African | Human | Partial | Sanger dideoxy sequencing |
| KX447520 | 2014 | French Polynesia | Asian | Human | Partial | IonTorrent |
| KX447521 | 2014 | French Polynesia | Asian | Human | Partial | IonTorrent |
| MK269353 | 2016 | USA | Asian | Human | Complete | Illumina |
| MK269354 | 2016 | USA | Asian | Human | Complete | Illumina |
| MK269355 | 2016 | USA | Asian | Human | Partial | Illumina |
| MK269356 | 2016 | USA | Asian | Human | Partial | Illumina |
| MK269357 | 2016 | USA | Asian | Human | Complete | Illumina |
| MK269358 | 2016 | USA | Asian | Human | Partial | Illumina |
| MK269359 | 2016 | USA | Asian | Human | Complete | Illumina |
| MK269360 | 2016 | USA | Asian | Human | Complete | Illumina |
| KF270887 | 2007 | Gabon | West African | Human | Partial | Sanger dideoxy sequencing |
| MK269361 | 2016 | USA | Asian | Human | Partial | Illumina |
| KX455424 | 2008 | Cambodia | Asian | Human | Partial | Sanger dideoxy sequencing |
| KX455425 | 2009 | Cambodia | Asian | Human | Partial | Sanger dideoxy sequencing |
| KX455426 | 2009 | Cambodia | Asian | Human | Partial | Sanger dideoxy sequencing |
| KY927808 | 2016 | China | Asian | Human | Complete | Sanger dideoxy sequencing |
| MK271800 | 2016 | Thailand | Asian | Mosquito | Partial |  |
| MK271801 | 2016 | Thailand | Asian | Mosquito | Partial |  |
| MK271802 | 2016 | Thailand | Asian | Mosquito | Partial |  |
| MK271803 | 2016 | Thailand | Asian | Mosquito | Partial |  |
| MK271804 | 2016 | Thailand | Asian | Mosquito | Partial |  |
| MK271805 | 2016 | Thailand | Asian | Mosquito | Partial |  |
| MK271806 | 2016 | Thailand | Asian | Mosquito | Partial |  |
| MK271807 | 2016 | Thailand | Asian | Mosquito | Partial |  |
| MK271808 | 2016 | Thailand | Asian | Mosquito | Partial |  |
| MK271809 | 2016 | Thailand | Asian | Mosquito | Partial |  |
| MK271810 | 2016 | Thailand | Asian | Mosquito | Partial |  |
| MK271811 | 2016 | Thailand | Asian | Mosquito | Partial |  |
| MK271812 | 2016 | Thailand | Asian | Mosquito | Partial |  |
| MK271813 | 2016 | Thailand | Asian | Mosquito | Partial |  |
| MK271814 | 2016 | Thailand | Asian | Mosquito | Partial |  |
| KF383015 | 2001 | Senegal | West African |  | Partial | Sanger dideoxy sequencing |
| MK271815 | 2016 | Thailand | Asian | Mosquito | Partial |  |
| MK271816 | 2016 | Thailand | Asian | Mosquito | Partial |  |
| MK271817 | 2016 | Thailand | Asian | Mosquito | Partial |  |
| MK271818 | 2016 | Thailand | Asian | Mosquito | Partial |  |
| MK271819 | 2016 | Thailand | Asian | Mosquito | Partial |  |
| MK271820 | 2016 | Thailand | Asian | Mosquito | Partial |  |
| MK271821 | 2016 | Thailand | Asian | Mosquito | Partial |  |
| MK271822 | 2016 | Thailand | Asian | Mosquito | Partial |  |
| MK271823 | 2016 | Thailand | Asian | Mosquito | Partial |  |
| MK271824 | 2016 | Thailand | Asian | Mosquito | Partial |  |
| KF383016 | 2001 | Senegal | West African |  | Partial | Sanger dideoxy sequencing |
| MK271825 | 2016 | Thailand | Asian | Mosquito | Partial |  |
| MK271826 | 2016 | Thailand | Asian | Mosquito | Partial |  |
| MK271827 | 2016 | Thailand | Asian | Mosquito | Partial |  |
| MK271828 | 2016 | Thailand | Asian | Mosquito | Partial |  |
| MK271829 | 2016 | Thailand | Asian | Mosquito | Partial |  |
| MK271830 | 2016 | Thailand | Asian | Mosquito | Partial |  |
| MK271831 | 2016 | Thailand | Asian | Mosquito | Partial |  |
| MK271832 | 2016 | Thailand | Asian | Mosquito | Partial |  |
| MK271833 | 2016 | Thailand | Asian | Mosquito | Partial |  |
| MK271834 | 2016 | Thailand | Asian | Mosquito | Partial |  |
| KF383017 | 2001 | Senegal | West African |  | Partial | Sanger dideoxy sequencing |
| MK271835 | 2016 | Thailand | Asian | Mosquito | Partial |  |
| MK271836 | 2016 | Thailand | Asian | Mosquito | Partial |  |
| MK271837 | 2016 | Thailand | Asian | Mosquito | Partial |  |
| MK271838 | 2016 | Thailand | Asian | Mosquito | Partial |  |
| MK271839 | 2016 | Thailand | Asian | Mosquito | Partial |  |
| MK271840 | 2016 | Thailand | Asian | Mosquito | Partial |  |
| MK271841 | 2016 | Thailand | Asian | Mosquito | Partial |  |
| MK271842 | 2016 | Thailand | Asian | Mosquito | Partial |  |
| MK271843 | 2016 | Thailand | Asian | Mosquito | Partial |  |
| MK271844 | 2016 | Thailand | Asian | Mosquito | Partial |  |
| KF383018 | 2000 | Senegal | West African |  | Partial | Sanger dideoxy sequencing |
| MK271845 | 2016 | Thailand | Asian | Mosquito | Partial |  |
| MK271846 | 2016 | Thailand | Asian | Mosquito | Partial |  |
| MK271847 | 2016 | Thailand | Asian | Mosquito | Partial |  |
| MK271848 | 2016 | Thailand | Asian | Mosquito | Partial |  |
| MK271849 | 2016 | Thailand | Asian | Mosquito | Partial |  |
| MK271850 | 2016 | Thailand | Asian | Mosquito | Partial |  |
| MK271851 | 2016 | Thailand | Asian | Mosquito | Partial |  |
| MK271852 | 2016 | Thailand | Asian | Mosquito | Partial |  |
| MK271853 | 2016 | Thailand | Asian | Mosquito | Partial |  |
| MK271854 | 2016 | Thailand | Asian | Mosquito | Partial |  |
| KF383019 | 1998 | Senegal | West African |  | Partial | Sanger dideoxy sequencing |
| MK271855 | 2016 | Thailand | Asian | Mosquito | Partial |  |
| MK271856 | 2016 | Thailand | Asian | Mosquito | Partial |  |
| MK271857 | 2016 | Thailand | Asian | Mosquito | Partial |  |
| MK271858 | 2016 | Thailand | Asian | Mosquito | Partial |  |
| MK271859 | 2016 | Thailand | Asian | Mosquito | Partial |  |
| MK271860 | 2016 | Thailand | Asian | Mosquito | Partial |  |
| MK271861 | 2016 | Thailand | Asian | Mosquito | Partial |  |
| MK271862 | 2016 | Thailand | Asian | Mosquito | Partial |  |
| MK271863 | 2016 | Thailand | Asian | Mosquito | Partial |  |
| MK271864 | 2016 | Thailand | Asian | Mosquito | Partial |  |
| KF383020 | 1980 | Cote dIvoire | West African |  | Partial | Sanger dideoxy sequencing |
| MK271865 | 2016 | Thailand | Asian | Mosquito | Partial |  |
| MK271866 | 2016 | Thailand | Asian | Mosquito | Partial |  |
| MK271867 | 2016 | Thailand | Asian | Mosquito | Partial |  |
| MK271868 | 2016 | Thailand | Asian | Mosquito | Partial |  |
| MK271869 | 2016 | Thailand | Asian | Mosquito | Partial |  |
| MK271870 | 2016 | Thailand | Asian | Mosquito | Partial |  |
| MK271871 | 2016 | Thailand | Asian | Mosquito | Partial |  |
| MK271872 | 2016 | Thailand | Asian | Mosquito | Partial |  |
| MK271873 | 2016 | Thailand | Asian | Mosquito | Partial |  |
| MK271874 | 2016 | Thailand | Asian | Mosquito | Partial |  |
| KF383021 | 1998 | Senegal | West African |  | Partial | Sanger dideoxy sequencing |
| MK271875 | 2016 | Thailand | Asian | Mosquito | Partial |  |
| MK271876 | 2016 | Thailand | Asian | Mosquito | Partial |  |
| MK271877 | 2016 | Thailand | Asian | Mosquito | Partial |  |
| MK271878 | 2016 | Thailand | Asian | Mosquito | Partial |  |
| KY962729 | 2016 | South Korea | Asian | Human | Partial | Illumina |
| KX520666 | 2015 | Brazil | Asian | Human | Complete | Sanger dideoxy sequencing |
| KT381874 | 2015 | Brazil | Asian | Human | Partial | Sanger dideoxy sequencing |
| MK282406 | 2016 | Thailand | Asian | Mosquito | Partial | Sanger dideoxy sequencing |
| MK282407 | 2016 | Thailand | Asian | Mosquito | Partial | Sanger dideoxy sequencing |
| MK282408 | 2016 | Thailand | Asian | Mosquito | Partial | Sanger dideoxy sequencing |
| KF383022 | 1997 | Senegal | West African |  | Partial | Sanger dideoxy sequencing |
| MK282409 | 2016 | Thailand | Asian | Mosquito | Partial | Sanger dideoxy sequencing |
| MK282410 | 2016 | Thailand | Asian | Mosquito | Partial | Sanger dideoxy sequencing |
| MK282411 | 2016 | Thailand | Asian | Mosquito | Partial | Sanger dideoxy sequencing |
| MK282412 | 2016 | Thailand | Asian | Human | Partial | Sanger dideoxy sequencing |
| KY967711 | 2016 | China | Asian | Human | Complete | Sanger dideoxy sequencing |
| KY968096 | 2016 | Brazil | Asian | Human | Partial | Sanger dideoxy sequencing |
| KY968097 | 2016 | Brazil | Asian | Human | Partial | Sanger dideoxy sequencing |
| KY968098 | 2016 | Brazil | Asian | Human | Partial | Sanger dideoxy sequencing |
| MG912101 | 2017 | Mexico | Asian | Human | Partial | Sanger dideoxy sequencing |
| MG912102 | 2017 | Mexico | Asian | Human | Partial | Sanger dideoxy sequencing |
| KF383023 | 1997 | Senegal | West African |  | Partial | Sanger dideoxy sequencing |
| KX548902 | 2015 | Colombia | Asian | Human | Complete | Illumina |
| KY989511 | 1947 | Uganda | East African | Laboratory | Complete |  |
| KY989971 | 2015 | Colombia | Asian | Human | Complete | Illumina |
| KX601166 | 1984 | Senegal | West African | Mosquito | Complete | Illumina |
| KX601167 | 1966 | Malaysia | Asian | Mosquito | Complete | Illumina |
| KX601168 | 2015 | Puerto Rico | Asian | Human | Complete | Illumina |
| KX601169 | 1947 | Uganda | East African | Animal | Complete | Illumina |
| MG967272 | 2014 | Cook Islands | Asian | Human | Partial | Sanger dideoxy sequencing |
| MG967273 | 2014 | Cook Islands | Asian | Human | Partial | Sanger dideoxy sequencing |
| MG967274 | 2014 | Cook Islands | Asian | Human | Partial | Sanger dideoxy sequencing |
| KF383024 | 1997 | Senegal | West African |  | Partial | Sanger dideoxy sequencing |
| MG967275 | 2014 | Cook Islands | Asian | Human | Partial | Sanger dideoxy sequencing |
| MG967276 | 2015 | Samoa | Asian | Human | Partial | Sanger dideoxy sequencing |
| MG967277 | 2015 | Samoa | Asian | Human | Partial | Sanger dideoxy sequencing |
| MG967278 | 2015 | Solomon Islands | Asian | Human | Partial | Sanger dideoxy sequencing |
| MG976700 | 2014 | French Polynesia | Asian | Human | Partial | Sanger dideoxy sequencing |
| MG982560 | 2014 | Chile | Asian | Human | Partial | Sanger dideoxy sequencing |
| MG982561 | 2014 | Chile | Asian | Human | Partial | Sanger dideoxy sequencing |
| MG982562 | 2014 | Chile | Asian | Human | Partial | Sanger dideoxy sequencing |
| MG982563 | 2014 | Chile | Asian | Human | Partial | Sanger dideoxy sequencing |
| MG982564 | 2014 | Chile | Asian | Human | Partial | Sanger dideoxy sequencing |
| KF383025 | 1997 | Senegal | West African |  | Partial | Sanger dideoxy sequencing |
| MG982565 | 2014 | Chile | Asian | Human | Partial | Sanger dideoxy sequencing |
| MG982566 | 2014 | Chile | Asian | Human | Partial | Sanger dideoxy sequencing |
| MG982567 | 2014 | Chile | Asian | Human | Partial | Sanger dideoxy sequencing |
| MK371391 | 2017 | Brazil | Asian | Mosquito | Partial | Sanger dideoxy sequencing |
| MG982568 | 2014 | Chile | Asian | Human | Partial | Sanger dideoxy sequencing |
| MG982569 | 2014 | Chile | Asian | Human | Partial | Sanger dideoxy sequencing |
| MK371392 | 2017 | Brazil | Asian | Mosquito | Partial | Sanger dideoxy sequencing |
| MK371393 | 2016 | Brazil | Asian | Mosquito | Partial | Sanger dideoxy sequencing |
| MG982570 | 2014 | Chile | Asian | Human | Partial | Sanger dideoxy sequencing |
| MG982571 | 2014 | Chile | Asian | Human | Partial | Sanger dideoxy sequencing |
| MW122432 | 2016 | Puerto Rico | Asian | Human | Complete | Illumina |
| KF383026 | 1997 | Senegal | West African |  | Partial | Sanger dideoxy sequencing |
| MG982572 | 2014 | Chile | Asian | Human | Partial | Sanger dideoxy sequencing |
| MG982573 | 2014 | Chile | Asian | Human | Partial | Sanger dideoxy sequencing |
| KX673530 | 2016 | United Kingdom | Asian | Human | Complete | Illumina |
| MK434848 | 2018 | Hungary | Asian | Human | Partial | Sanger dideoxy sequencing |
| KX694532 | 2013 | Thailand | Asian | Human | Complete | Illumina |
| KX694533 | 1966 | Malaysia | Asian | Mosquito | Complete | Illumina |
| KX694534 | 2015 | Honduras | Asian | Human | Complete | Illumina |
| KX702400 | 2016 | Venezuela | Asian | Human | Complete | Sanger dideoxy sequencing |
| KX766028 | 2016 | Dominican Republic | Asian | Human | Complete | IonTorrent |
| KX766029 | 2016 | Mexico | Asian | Human | Complete | IonTorrent |
| KF383027 | 1997 | Senegal | West African |  | Partial | Sanger dideoxy sequencing |
| LC002520 |  | Uganda | East African | Animal | Complete |  |
| KR815989 | 2015 | Brazil | Asian | Human | Partial | Sanger dideoxy sequencing |
| KR815990 | 2015 | Brazil | Asian | Human | Partial | Sanger dideoxy sequencing |
| KR816333 | 2015 | Brazil | Asian | Human | Partial | Sanger dideoxy sequencing |
| KR816334 | 2015 | Brazil | Asian | Human | Partial | Sanger dideoxy sequencing |
| KR816335 | 2015 | Brazil | Asian | Human | Partial | Sanger dideoxy sequencing |
| KR816336 | 2015 | Brazil | Asian | Human | Partial | Sanger dideoxy sequencing |
| KX806557 | 2016 | Australia | Asian | Human | Complete | Illumina |
| KX811222 | 2016 | Brazil | Asian | Laboratory | Complete | Illumina |
| KX813683 | 2016 | Singapore | Asian | Human | Complete | Sanger dideoxy sequencing |
| KF383028 | 2002 | Senegal | West African |  | Partial | Sanger dideoxy sequencing |
| KR872956 | 2015 | Brazil | Asian | Human | Complete | ION |
| MH013290 | 2017 | Thailand | Asian | Human | Complete | Illumina |
| KX827268 | 2016 | USA | Asian | Human | Complete | Illumina |
| KX827309 | 2016 | Singapore | Asian | Human | Complete | Sanger dideoxy sequencing |
| KX830930 | 2016 | Brazil | Asian | Human | Complete | Illumina |
| KX832731 | 2016 | USA | Asian | Human | Complete | Illumina |
| KX838904 | 2016 | USA | Asian | Mosquito | Complete | Illumina |
| KX838905 | 2016 | USA | Asian | Mosquito | Complete | Illumina |
| KX838906 | 2016 | USA | Asian | Mosquito | Complete | Illumina |
| KF383029 | 2002 | Senegal | West African |  | Partial | Sanger dideoxy sequencing |
| KX842449 | 2016 | USA | Asian | Human | Complete | Illumina |
| MH027651 | 2017 | Brazil | Asian | Mosquito | Partial | Sanger dideoxy sequencing |
| KX856011 | 2016 | Mexico | Asian | Mosquito | Complete |  |
| KX867786 | 2016 | Russia | Asian | Human | Partial | Sanger dideoxy sequencing |
| KX879603 | 2016 | Ecuador | Asian | Human | Complete | Illumina; Oxford Nanopore Technologies, |
| KX879604 | 2016 | Ecuador | Asian | Human | Complete | Illumina; Oxford Nanopore Technologies, |
| KX893855 | 2016 | Venezuela | Asian | Human | Complete | Sanger dideoxy sequencing |
| KX906952 | 2016 | Honduras | Asian | Human | Complete | Ion Torrent S5, Illumina MiSeq |
| KX906953 | 2016 | Malaysia | Asian | Human | Partial | Sanger dideoxy sequencing |
| KX906954 | 2016 | Malaysia | Asian | Human | Partial | Sanger dideoxy sequencing |
| KF383030 | 1981 | Burkina Faso | West African |  | Partial | Sanger dideoxy sequencing |
| KX906955 | 2016 | Malaysia | Asian | Human | Partial | Sanger dideoxy sequencing |
| KX906956 | 2016 | Malaysia | Asian | Human | Partial | Sanger dideoxy sequencing |
| KX922703 | 2016 | USA | Asian | Human | Complete | Illumina |
| KX922704 | 2016 | USA | Asian | Human | Complete | Illumina |
| KX922705 | 2016 | USA | Asian | Human | Complete | Illumina |
| KX922706 | 2016 | USA | Asian | Human | Complete | Illumina |
| KX922707 | 2016 | USA | Asian | Human | Complete | Illumina |
| KX922708 | 2016 | USA | Asian | Mosquito | Complete | Illumina |
| KF383031 | 1969 | Senegal | West African |  | Partial | Sanger dideoxy sequencing |
| KX928077 | 2016 | Honduras | Asian | Human | Partial | Sanger dideoxy sequencing |
| KF383032 | 1979 | Senegal | West African |  | Partial | Sanger dideoxy sequencing |
| MH055376 | 2016 | China | Asian |  | Complete |  |
| KF383033 | 1979 | Senegal | West African |  | Partial | Sanger dideoxy sequencing |
| KF383034 | 1979 | Senegal | West African |  | Partial | Sanger dideoxy sequencing |
| KF383035 | 1963 | Uganda | East African |  | Partial | Sanger dideoxy sequencing |
| MW122433 | 2016 | Puerto Rico | Asian | Human | Complete | Illumina |
| KF383036 | 1999 | Cote dIvoire | East African |  | Partial | Sanger dideoxy sequencing |
| KF383037 | 1996 | Cote dIvoire | East African |  | Partial | Sanger dideoxy sequencing |
| KF383038 | 1999 | Cote dIvoire | East African |  | Partial | Sanger dideoxy sequencing |
| KF383039 | 1991 | Senegal | West African |  | Partial | Sanger dideoxy sequencing |
| MH063259 | 2017 | Cuba | Asian | Human | Complete | Illumina |
| KF383040 | 1990 | Cote dIvoire | West African |  | Partial | Sanger dideoxy sequencing |
| MH063260 | 2017 | Cuba | Asian | Human | Complete | Illumina |
| MH063261 | 2017 | Cuba | Asian | Human | Complete | Illumina |
| MH063262 | 2017 | Cuba | Asian | Human | Complete | Illumina |
| MH063263 | 2017 | Cuba | Asian | Human | Partial | Illumina |
| MH063264 | 2017 | Cuba | Asian | Human | Complete | Illumina |
| KX986760 | 2016 | Brazil | Asian | Mosquito | Partial | Illumina |
| KX986761 | 2016 | Brazil | Asian | Mosquito | Partial | Illumina |
| KF383041 | 1990 | Cote dIvoire | West African |  | Partial | Sanger dideoxy sequencing |
| KY003152 | 2016 | Philippines | Asian | Human | Partial | Sanger dideoxy sequencing |
| KY003153 | 2016 | Italy | Asian | Human | Complete | Sanger dideoxy sequencing |
| KY003154 | 2016 | Italy | Asian | Human | Complete | Sanger dideoxy sequencing |
| KY003155 | 2016 | Italy | Asian | Human | Partial | Sanger dideoxy sequencing |
| KY003156 | 2016 | Italy | Asian | Human | Partial | Sanger dideoxy sequencing |
| KY003157 | 2016 | Italy | Asian | Human | Partial | Sanger dideoxy sequencing |
| KY005879 | 2016 | Ecuador | Asian | Mosquito | Partial |  |
| KY007221 | 2016 | Thailand | Asian | Human | Partial | Sanger dideoxy sequencing |
| KY014295 | 2016 | USA | Asian | Human | Complete | Illumina; Swift LC |
| KY014296 | 2016 | Brazil | Asian | Human | Complete | Illumina; Swift LC |
| KF383042 | 1990 | Cote dIvoire | West African |  | Partial | Sanger dideoxy sequencing |
| KY014297 | 2016 | Brazil | Asian | Human | Complete | Illumina; Swift LC |
| KY014298 | 2016 | USA | Asian | Human | Complete | Illumina; Swift LC |
| KY014299 | 2016 | USA | Asian | Mosquito | Complete | Illumina; Swift LC |
| KY014300 | 2016 | Dominican Republic | Asian | Human | Complete | Illumina; Swift LC |
| KY014301 | 2016 | Brazil | Asian | Human | Complete | Illumina; Swift LC |
| KY014302 | 2016 | Dominican Republic | Asian | Human | Complete | Illumina; Swift LC |
| KY014303 | 2016 | Dominican Republic | Asian | Human | Complete | Illumina; Swift LC |
| KY014304 | 2016 | Dominican Republic | Asian | Human | Complete | Illumina; Swift LC |
| KY014305 | 2016 | Dominican Republic | Asian | Human | Complete | Illumina; Swift LC |
| KY014306 | 2016 | Honduras | Asian | Human | Complete | Illumina; Swift LC |
| KF383043 | 1990 | Cote dIvoire | West African |  | Partial | Sanger dideoxy sequencing |
| KY014307 | 2016 | Brazil | Asian | Human | Complete | Illumina; Swift LC |
| KY014308 | 2016 | Brazil | Asian | Human | Complete | Illumina; Swift LC |
| KY014309 | 2016 | Brazil | Asian | Human | Partial | Illumina; Swift LC |
| KY014310 | 2016 | Honduras | Asian | Human | Complete | Illumina; Swift LC |
| KY014311 | 2016 | Honduras | Asian | Human | Partial | Illumina; Swift LC |
| KY014312 | 2016 | Honduras | Asian | Human | Complete | Illumina; Swift LC |
| KY014313 | 2016 | Brazil | Asian | Human | Complete | Illumina; Swift LC |
| KY014314 | 2016 | Dominican Republic | Asian | Human | Complete | Illumina; Swift LC |
| KY014315 | 2016 | Honduras | Asian | Human | Complete | Illumina; Swift LC |
| KY014316 | 2016 | USA | Asian | Human | Complete | Illumina; Swift LC |
| KF383044 | 1990 | Cote dIvoire | West African |  | Partial | Sanger dideoxy sequencing |
| KY014317 | 2016 | Brazil | Asian | Human | Complete | Illumina; Swift LC |
| KY014318 | 2016 | Dominican Republic | Asian | Human | Complete | Illumina; Swift LC |
| KY014319 | 2016 | Honduras | Asian | Human | Complete | Illumina; Swift LC |
| KY014320 | 2016 | Brazil | Asian | Human | Complete | Illumina; Swift LC |
| KY014321 | 2016 | Dominican Republic | Asian | Human | Complete | Illumina; Swift LC |
| KY014323 | 2016 | USA | Asian | Mosquito | Complete | Illumina; Swift LC |
| KY014324 | 2016 | USA | Asian | Mosquito | Complete | Illumina; Swift LC |
| KY014325 | 2016 | USA | Asian | Human | Complete | Illumina; Swift LC |
| KY014326 | 2016 | USA | Asian | Human | Complete | Illumina; Swift LC |
| KF383045 | 1990 | Cote dIvoire | West African |  | Partial | Sanger dideoxy sequencing |
| KY014327 | 2016 | Honduras | Asian | Human | Complete | Illumina; Swift LC |
| KY014328 | 2016 | Italy | Asian | Human | Partial | Sanger dideoxy sequencing |
| KY014329 | 2016 | Italy | Asian | Human | Partial | Sanger dideoxy sequencing |
| MH119185 | 2016 | Thailand | Asian | Human | Complete | Illumina |
| MH130042 | 2016 | Malaysia | Asian | Human | Partial | Sanger dideoxy sequencing |
| MH130043 | 2016 | Malaysia | Asian | Human | Partial | Sanger dideoxy sequencing |
| MH130044 | 2016 | Malaysia | Asian | Human | Partial | Sanger dideoxy sequencing |
| KY042039 | 2016 | Brazil | Asian | Human | Partial |  |
| MW122434 | 2016 | Puerto Rico | Asian | Human | Complete | Illumina |
| KF383046 | 1999 | Cote dIvoire | East African |  | Partial | Sanger dideoxy sequencing |
| KY042040 | 2016 | Philippines | Asian | Human | Partial |  |
| KY042041 | 2016 | Philippines | Asian | Human | Partial | Sanger dideoxy sequencing |
| KY042042 | 2016 | Philippines | Asian | Human | Partial |  |
| KY042043 | 2016 | Guatemala | Asian | Human | Partial | Sanger dideoxy sequencing |
| KY042044 | 2016 | Puerto Rico | Asian | Human | Partial | Sanger dideoxy sequencing |
| KF383047 | 1991 | Senegal | West African |  | Partial | Sanger dideoxy sequencing |
| KY042045 | 2016 | Viet Nam | Asian | Human | Partial | Sanger dideoxy sequencing |
| KF383048 | 1969 | Senegal | West African |  | Partial | Sanger dideoxy sequencing |
| KY064008 | 2014 | Bangladesh | Asian | Human | Partial | Sanger dideoxy sequencing |
| MH157195 | 2016 | Mexico | Asian | Human | Complete | Ion Torrent;Illumina |
| MH157196 | 2016 | Mexico | Asian | Human | Complete | Ion Torrent;Illumina |
| MH157197 | 2016 | Mexico | Asian | Human | Complete | Ion Torrent;Illumina |
| MH157198 | 2016 | Mexico | Asian | Human | Complete | Illumina |
| MH157199 | 2016 | Mexico | Asian | Human | Complete | Ion Torrent;Illumina |
| MH157200 | 2016 | Mexico | Asian | Human | Complete | Ion Torrent;Illumina |
| MH157201 | 2016 | Mexico | Asian | Human | Complete | Ion Torrent;Illumina |
| MH157202 | 2016 | Mexico | Asian | Human | Complete | Ion Torrent;Illumina |
| MH157203 | 2016 | Mexico | Asian | Human | Complete | Ion Torrent;Illumina |
| KF383049 | 1968 | Senegal | West African |  | Partial | Sanger dideoxy sequencing |
| MH157204 | 2016 | Mexico | Asian | Human | Complete | Ion Torrent;Illumina |
| MN473450 | 2016 | Canada | Asian | Human | Partial | Illumina |
| MH157205 | 2016 | Mexico | Asian | Human | Complete | Ion Torrent;Illumina |
| MN473451 | 2016 | Canada | Asian | Human | Complete | Illumina |
| MN473452 | 2016 | Canada | Asian | Human | Complete | Illumina |
| MH157206 | 2016 | Mexico | Asian | Human | Complete | Ion Torrent;Illumina |
| MH157207 | 2016 | Mexico | Asian | Human | Complete | Ion Torrent;Illumina |
| MN473453 | 2016 | Canada | Asian | Human | Complete | Illumina |
| MH157208 | 2016 | Mexico | Asian | Human | Complete | Ion Torrent;Illumina |
| MN473454 | 2016 | Canada | Asian | Human | Complete | Illumina |
| KF383050 | 1979 | Senegal | West African |  | Partial | Sanger dideoxy sequencing |
| MH157209 | 2016 | Mexico | Asian | Human | Complete | Ion Torrent;Illumina |
| MH157210 | 2016 | Mexico | Asian | Human | Complete | Ion Torrent;Illumina |
| MH157211 | 2016 | Mexico | Asian | Human | Complete | Ion Torrent;Illumina |
| MH157212 | 2016 | Mexico | Asian | Human | Complete | Ion Torrent;Illumina |
| MH157213 | 2016 | Mexico | Asian | Human | Complete | Ion Torrent;Illumina |
| MH157214 | 2016 | Mexico | Asian | Human | Complete | Ion Torrent;Illumina |
| MH158236 | 2010 | Cambodia | Asian | Human | Complete | Sanger dideoxy sequencing |
| MH158237 | 2015 | Puerto Rico | Asian | Human | Complete | Sanger dideoxy sequencing |
| KF383051 | 1979 | Senegal | West African |  | Partial | Sanger dideoxy sequencing |
| KF383052 | 2002 | Senegal | West African |  | Partial | Sanger dideoxy sequencing |
| MN527290 | 2017 | Thailand | Asian | Mosquito | Partial | Sanger dideoxy sequencing |
| MW122416 | 2016 | Puerto Rico | Asian | Human | Complete | Illumina |
| KF383053 | 2002 | Senegal | West African |  | Partial | Sanger dideoxy sequencing |
| KM078977 | 2014 | Chile | Asian | Human | Partial |  |
| KM078976 | 2014 | Chile | Asian | Human | Partial |  |
| KM078971 | 2014 | Chile | Asian | Human | Partial |  |
| KM078970 | 2014 | Chile | Asian | Human | Partial |  |
| KM078968 | 2014 | Chile | Asian | Human | Partial |  |
| KM078966 | 2014 | Chile | Asian | Human | Partial |  |
| KM078965 | 2014 | Chile | Asian | Human | Partial |  |
| KM078964 | 2014 | Chile | Asian | Human | Partial |  |
| KM078963 | 2014 | Chile | Asian | Human | Partial |  |
| KM078961 | 2014 | Chile | Asian | Human | Partial |  |
| KM078941 | 2014 | Chile | Asian | Human | Partial |  |
| KM078933 | 2014 | Chile | Asian | Human | Partial |  |
| KM078932 | 2014 | Chile | Asian | Human | Partial |  |
| KM014700 | 2014 | French Polynesia | Asian | Human | Partial |  |
| KM212961 | 2014 | New Caledonia | Asian | Human | Partial | Sanger dideoxy sequencing |
| KM212964 | 2014 | New Caledonia | Asian | Human | Partial | Sanger dideoxy sequencing |
| KJ873160 | 2014 | New Caledonia | Asian | Human | Partial | Sanger dideoxy sequencing |
| KM851038 | 2012 | Philippines | Asian | Human | Partial | Illumina |
| KY241768 | 2016 | Singapore | Asian | Human | Complete | Illumina |
| KY241724 | 2016 | Singapore | Asian | Human | Complete | Illumina |
| MW122436 | 2016 | Puerto Rico | Asian | Human | Complete | Illumina |
| KF383056 | 2001 | Senegal | West African |  | Partial | Sanger dideoxy sequencing |
| KY241720 | 2016 | Singapore | Asian | Human | Complete | Illumina |
| MZ008356 | 2019 | Cambodia | Asian | Human | Complete |  |
| MZ126476 | 2016 | Barbados | Asian | Mosquito | Partial | Illumina |
| MW143022 | 1947 | Uganda | East African | Laboratory | Complete | Oxford Nanopore MinION |
| MW143020 | 1947 | Uganda | East African | Laboratory | Complete | Oxford Nanopore MinION |
| KF383057 | 2001 | Senegal | West African |  | Partial | Sanger dideoxy sequencing |
| MW143017 | 1947 | Uganda | East African | Laboratory | Complete | Oxford Nanopore MinION |
| MW915414 | 2016 | Viet Nam | Asian | Human | Complete | IonTorrent |
| MW915412 | 2016 | Viet Nam | Asian | Human | Complete | IonTorrent |
| MW915410 | 2017 | Viet Nam | Asian | Human | Partial | IonTorrent |
| MW894613 | 2017 | Colombia | Asian | Mosquito | Partial | Sanger dideoxy sequencing |
| MW894612 | 2017 | Colombia | Asian | Mosquito | Partial | Sanger dideoxy sequencing |
| MW894610 | 2017 | Colombia | Asian | Mosquito | Partial | Sanger dideoxy sequencing |
| MT482109 | 2009 | Uganda | Asian | Animal | Partial | Sanger dideoxy sequencing |
| MT482108 | 2013 | Uganda | Asian | Animal | Partial | Sanger dideoxy sequencing |
| MT482107 | 2011 | Uganda | Asian | Animal | Partial | Sanger dideoxy sequencing |
| MT482106 | 2011 | Uganda | Asian | Animal | Partial | Sanger dideoxy sequencing |
| MW680970 | 2018 | China | Asian | Human | Complete | Sanger dideoxy sequencing |
| MW680969 | 2018 | China | Asian | Human | Complete |  |
| KF383059 | 2000 | Senegal | West African |  | Partial | Sanger dideoxy sequencing |
| MT377504 | 2006 | Thailand | Asian | Human | Complete | Sanger dideoxy sequencing |
| MT377503 | 2015 | Thailand | Asian | Human | Complete | Sanger dideoxy sequencing |
| MT377502 | 2006 | Thailand | Asian | Human | Complete | Sanger dideoxy sequencing |
| MT377500 | 2016 | Thailand | Asian | Human | Complete | Sanger dideoxy sequencing |
| MT377497 | 2016 | Thailand | Asian | Human | Complete | Sanger dideoxy sequencing |
| MT377496 | 2016 | Thailand | Asian | Human | Complete | Sanger dideoxy sequencing |
| MT377495 | 2017 | Thailand | Asian | Human | Complete | Sanger dideoxy sequencing |
| MT377493 | 2017 | Thailand | Asian | Human | Complete | Sanger dideoxy sequencing |
| MZ318419 | 2008 | Thailand | Asian | Human | Partial | Sanger dideoxy sequencing |
| MZ318418 | 2008 | Thailand | Asian | Human | Partial | Sanger dideoxy sequencing |
| KF383061 | 1998 | Senegal | West African |  | Partial | Sanger dideoxy sequencing |
| MZ318416 | 2008 | Thailand | Asian | Human | Partial | Sanger dideoxy sequencing |
| MZ318412 | 2008 | Thailand | Asian | Human | Partial | Sanger dideoxy sequencing |
| MZ318411 | 2009 | Thailand | Asian | Human | Partial | Sanger dideoxy sequencing |
| MZ318410 | 2009 | Thailand | Asian | Human | Partial | Sanger dideoxy sequencing |
| MZ318408 | 2007 | Thailand | Asian | Human | Partial | Sanger dideoxy sequencing |
| MZ318405 | 2009 | Thailand | Asian | Human | Partial | Sanger dideoxy sequencing |
| MZ318404 | 2009 | Thailand | Asian | Human | Partial | Sanger dideoxy sequencing |
| MW389537 | 2015 | China | Asian | Animal | Partial | Sanger dideoxy sequencing |
| KF383063 | 1997 | Senegal | West African |  | Partial | Sanger dideoxy sequencing |
| MW389536 | 2016 | China | Asian | Animal | Partial | Sanger dideoxy sequencing |
| MW389535 | 2016 | China | Asian | Animal | Partial | Sanger dideoxy sequencing |
| MW389534 | 2015 | China | Asian | Animal | Partial | Sanger dideoxy sequencing |
| MW389533 | 2015 | China | Asian | Animal | Partial | Sanger dideoxy sequencing |
| MW389532 | 2015 | China | Asian | Animal | Partial | Sanger dideoxy sequencing |
| OK054369 | 2016 | Nicaragua | Asian | Human | Partial | Illumina |
| OK054376 | 2016 | Nicaragua | Asian | Human | Partial | Illumina |
| OK054375 | 2016 | Nicaragua | Asian | Human | Partial | Illumina |
| OK054374 | 2016 | Nicaragua | Asian | Human | Partial | Illumina |
| OK054377 | 2016 | Nicaragua | Asian | Human | Partial | Illumina |
| OK054378 | 2016 | Nicaragua | Asian | Human | Partial | Illumina |
| OK054379 | 2016 | Nicaragua | Asian | Human | Partial | Illumina |
| KF383065 | 1997 | Senegal | West African |  | Partial | Sanger dideoxy sequencing |
| OK054382 | 2016 | Nicaragua | Asian | Human | Partial | Illumina |
| OK054385 | 2016 | Nicaragua | Asian | Human | Partial | Illumina |
| OK054388 | 2016 | Nicaragua | Asian | Human | Partial | Illumina |
| OK054387 | 2016 | Nicaragua | Asian | Human | Partial | Illumina |
| OK054389 | 2016 | Nicaragua | Asian | Human | Partial | Illumina |
| MW122435 | 2016 | Puerto Rico | Asian | Human | Complete | Illumina |
| KF383066 | 1997 | Senegal | West African |  | Partial | Sanger dideoxy sequencing |
| OK054390 | 2016 | Nicaragua | Asian | Human | Partial | Illumina |
| OK054391 | 2016 | Nicaragua | Asian | Human | Partial | Illumina |
| OK054397 | 2016 | Nicaragua | Asian | Human | Partial | Illumina |
| OK054398 | 2016 | Nicaragua | Asian | Human | Partial | Illumina |
| KF383067 | 1997 | Senegal | West African |  | Partial | Sanger dideoxy sequencing |
| OK054400 | 2016 | Nicaragua | Asian | Human | Partial | Illumina |
| OK054399 | 2016 | Nicaragua | Asian | Human | Partial | Illumina |
| OK054401 | 2016 | Nicaragua | Asian | Human | Partial | Illumina |
| OK054403 | 2016 | Nicaragua | Asian | Human | Partial | Illumina |
| OK054404 | 2016 | Nicaragua | Asian | Human | Partial | Illumina |
| OK054402 | 2016 | Nicaragua | Asian | Human | Partial | Illumina |
| OK054407 | 2016 | Nicaragua | Asian | Human | Partial | Illumina |
| OK054405 | 2016 | Nicaragua | Asian | Human | Partial | Illumina |
| OK054406 | 2016 | Nicaragua | Asian | Human | Partial | Illumina |
| KF383068 | 1997 | Senegal | West African |  | Partial | Sanger dideoxy sequencing |
| OK054408 | 2016 | Nicaragua | Asian | Human | Partial | Illumina |
| OK054410 | 2016 | Nicaragua | Asian | Human | Partial | Illumina |
| OK054411 | 2016 | Nicaragua | Asian | Human | Partial | Illumina |
| OK054412 | 2016 | Nicaragua | Asian | Human | Partial | Illumina |
| OK054415 | 2016 | Nicaragua | Asian | Human | Partial | Illumina |
| OK054414 | 2016 | Nicaragua | Asian | Human | Partial | Illumina |
| OK054416 | 2016 | Nicaragua | Asian | Human | Partial | Illumina |
| OK054419 | 2016 | Nicaragua | Asian | Human | Partial | Illumina |
| KF383069 | 1997 | Senegal | West African |  | Partial | Sanger dideoxy sequencing |
| OK054417 | 2016 | Nicaragua | Asian | Human | Partial | Illumina |
| OK054418 | 2016 | Nicaragua | Asian | Human | Partial | Illumina |
| OK054422 | 2016 | Nicaragua | Asian | Human | Partial | Illumina |
| OK054420 | 2016 | Nicaragua | Asian | Human | Partial | Illumina |
| OK054421 | 2016 | Nicaragua | Asian | Human | Partial | Illumina |
| OK054424 | 2016 | Nicaragua | Asian | Human | Partial | Illumina |
| OK054423 | 2016 | Nicaragua | Asian | Human | Partial | Illumina |
| OK054425 | 2016 | Nicaragua | Asian | Human | Partial | Illumina |
| KF383070 | 1968 | Central African Republic | East African |  | Partial | Sanger dideoxy sequencing |
| OK054428 | 2016 | Nicaragua | Asian | Human | Partial | Illumina |
| OK054429 | 2016 | Nicaragua | Asian | Human | Partial | Illumina |
| OK054430 | 2016 | Nicaragua | Asian | Human | Partial | Illumina |
| OK054432 | 2016 | Nicaragua | Asian | Human | Partial | Illumina |
| OK054431 | 2016 | Nicaragua | Asian | Human | Partial | Illumina |
| OK054433 | 2016 | Nicaragua | Asian | Human | Partial | Illumina |
| OK054435 | 2016 | Nicaragua | Asian | Human | Partial | Illumina |
| OK054434 | 2016 | Nicaragua | Asian | Human | Partial | Illumina |
| OK054436 | 2016 | Nicaragua | Asian | Human | Partial | Illumina |
| OK054437 | 2016 | Nicaragua | Asian | Human | Partial | Illumina |
| KF383071 | 1999 | Cote dIvoire | East African |  | Partial | Sanger dideoxy sequencing |
| OK054439 | 2016 | Nicaragua | Asian | Human | Partial | Illumina |
| OK054438 | 2016 | Nicaragua | Asian | Human | Partial | Illumina |
| OK054443 | 2016 | Nicaragua | Asian | Human | Partial | Illumina |
| OK054441 | 2016 | Nicaragua | Asian | Human | Partial | Illumina |
| OK054440 | 2016 | Nicaragua | Asian | Human | Partial | Illumina |
| OK054442 | 2016 | Nicaragua | Asian | Human | Partial | Illumina |
| OK054444 | 2016 | Nicaragua | Asian | Human | Partial | Illumina |
| OK054445 | 2016 | Nicaragua | Asian | Human | Partial | Illumina |
| OK054447 | 2016 | Nicaragua | Asian | Human | Partial | Illumina |
| OK054446 | 2016 | Nicaragua | Asian | Human | Partial | Illumina |
| KF383072 | 1999 | Cote dIvoire | East African |  | Partial | Sanger dideoxy sequencing |
| OK054450 | 2016 | Nicaragua | Asian | Human | Partial | Illumina |
| OK054451 | 2016 | Nicaragua | Asian | Human | Partial | Illumina |
| OK054448 | 2016 | Nicaragua | Asian | Human | Partial | Illumina |
| OK054449 | 2016 | Nicaragua | Asian | Human | Partial | Illumina |
| OK054452 | 2016 | Nicaragua | Asian | Human | Partial | Illumina |
| OK054453 | 2016 | Nicaragua | Asian | Human | Partial | Illumina |
| OK054454 | 2016 | Nicaragua | Asian | Human | Partial | Illumina |
| OK054457 | 2016 | Nicaragua | Asian | Human | Partial | Illumina |
| OK054455 | 2016 | Nicaragua | Asian | Human | Partial | Illumina |
| OK054456 | 2016 | Nicaragua | Asian | Human | Partial | Illumina |
| KF383073 | 1999 | Cote dIvoire | East African |  | Partial | Sanger dideoxy sequencing |
| OK054458 | 2016 | Nicaragua | Asian | Human | Partial | Illumina |
| OK054460 | 2016 | Nicaragua | Asian | Human | Partial | Illumina |
| OK054459 | 2016 | Nicaragua | Asian | Human | Partial | Illumina |
| OK054461 | 2016 | Nicaragua | Asian | Human | Partial | Illumina |
| OK054463 | 2016 | Nicaragua | Asian | Human | Partial | Illumina |
| OK054462 | 2016 | Nicaragua | Asian | Human | Partial | Illumina |
| OK054465 | 2016 | Nicaragua | Asian | Human | Partial | Illumina |
| OK054464 | 2016 | Nicaragua | Asian | Human | Partial | Illumina |
| OK054466 | 2016 | Nicaragua | Asian | Human | Partial | Illumina |
| OK054468 | 2016 | Nicaragua | Asian | Human | Partial | Illumina |
| KF383074 | 1996 | Cote dIvoire | East African |  | Partial | Sanger dideoxy sequencing |
| OK054467 | 2016 | Nicaragua | Asian | Human | Partial | Illumina |
| OK054469 | 2016 | Nicaragua | Asian | Human | Partial | Illumina |
| OK054470 | 2016 | Nicaragua | Asian | Human | Partial | Illumina |
| OK054471 | 2016 | Nicaragua | Asian | Human | Partial | Illumina |
| OK054472 | 2016 | Nicaragua | Asian | Human | Partial | Illumina |
| OK054473 | 2016 | Nicaragua | Asian | Human | Partial | Illumina |
| OK054474 | 2016 | Nicaragua | Asian | Human | Partial | Illumina |
| OK054475 | 2016 | Nicaragua | Asian | Human | Partial | Illumina |
| OK054478 | 2016 | Nicaragua | Asian | Human | Partial | Illumina |
| OK054477 | 2016 | Nicaragua | Asian | Human | Partial | Illumina |
| KF383075 | 1990 | Cote dIvoire | West African |  | Partial | Sanger dideoxy sequencing |
| OK054476 | 2016 | Nicaragua | Asian | Human | Partial | Illumina |
| OK054479 | 2016 | Nicaragua | Asian | Human | Partial | Illumina |
| OK054481 | 2016 | Nicaragua | Asian | Human | Partial | Illumina |
| OK054480 | 2016 | Nicaragua | Asian | Human | Partial | Illumina |
| OK054484 | 2016 | Nicaragua | Asian | Human | Partial | Illumina |
| OK054482 | 2016 | Nicaragua | Asian | Human | Partial | Illumina |
| OK054483 | 2016 | Nicaragua | Asian | Human | Partial | Illumina |
| OK054485 | 2016 | Nicaragua | Asian | Human | Partial | Illumina |
| OK054486 | 2016 | Nicaragua | Asian | Human | Partial | Illumina |
| OK054487 | 2016 | Nicaragua | Asian | Human | Partial | Illumina |
| MW122437 | 2016 | Puerto Rico | Asian | Human | Complete | Illumina |
| KF383076 | 1990 | Cote dIvoire | West African |  | Partial | Sanger dideoxy sequencing |
| MT437401 | 2019 | Brazil | East African | Animal | Partial | Illumina |
| OK571913 | 2014 | Haiti | Asian | Human | Complete | Sanger dideoxy sequencing |
| MW123921 | 2016 | Brazil | Asian | Human | Partial | Illumina |
| MW123922 | 2016 | Brazil | Asian | Human | Partial | Illumina |
| MW123923 | 2016 | Brazil | Asian | Human | Partial | Illumina |
| MW123924 | 2016 | Brazil | Asian | Human | Partial | Illumina |
| MW123925 | 2015 | Brazil | Asian | Human | Partial | Illumina |
| MW123926 | 2015 | Brazil | Asian | Human | Partial | Illumina |
| OL414716 | 2018 | Guinea | West African | Human | Complete | Sanger dideoxy sequencing |
| OL423647 | 2016 | Brazil | Asian | Human | Partial |  |
| KF383078 | 1981 | Burkina Faso | West African |  | Partial | Sanger dideoxy sequencing |
| OL423648 | 2016 | Brazil | Asian | Human | Partial |  |
| OL423649 | 2016 | Brazil | Asian | Human | Partial |  |
| OL423650 | 2016 | Brazil | Asian | Human | Partial |  |
| OL423651 | 2016 | Brazil | Asian | Human | Partial |  |
| OL423652 | 2016 | Brazil | Asian | Human | Partial |  |
| OL423653 | 2016 | Brazil | Asian | Human | Partial |  |
| OL423654 | 2016 | Brazil | Asian | Human | Partial |  |
| OL423655 | 2016 | Brazil | Asian | Human | Partial |  |
| OL423656 | 2017 | Brazil | Asian | Human | Partial |  |
| OL423657 | 2017 | Brazil | Asian | Human | Partial |  |
| KF383077 | 1990 | Cote dIvoire | West African |  | Partial | Sanger dideoxy sequencing |
| OL423658 | 2017 | Brazil | Asian | Human | Partial |  |
| OL423659 | 2017 | Brazil | Asian | Human | Partial |  |
| OL423660 | 2017 | Brazil | Asian | Human | Partial |  |
| OL423661 | 2017 | Brazil | Asian | Human | Partial |  |
| OL423662 | 2017 | Brazil | Asian | Human | Partial |  |
| OL423663 | 2018 | Brazil | Asian | Human | Partial |  |
| OL423664 | 2017 | Brazil | Asian | Human | Partial |  |
| OL423665 | 2018 | Brazil | Asian | Human | Partial |  |
| OL423666 | 2018 | Brazil | Asian | Human | Partial |  |
| OL423667 | 2018 | Brazil | Asian | Human | Partial |  |
| KF383079 | 1990 | Cote dIvoire | West African |  | Partial | Sanger dideoxy sequencing |
| OL423668 | 2017 | Brazil | Asian | Human | Partial |  |
| OL423669 | 2016 | Brazil | Asian | Human | Partial |  |
| OL450364 | 2015 | Haiti | Asian | Human | Complete | Sanger dideoxy sequencing |
| OL450365 | 2015 | Haiti | Asian | Human | Complete | Sanger dideoxy sequencing |
| OL450366 | 2015 | Haiti | Asian | Human | Complete | Sanger dideoxy sequencing |
| OM831146 | 2016 | Ecuador | Asian | Mosquito | Partial | Sanger dideoxy sequencing |
| OM831147 | 2016 | Ecuador | Asian | Mosquito | Partial | Sanger dideoxy sequencing |
| OM831148 | 2017 | Ecuador | Asian | Mosquito | Partial | Sanger dideoxy sequencing |
| OM831149 | 2017 | Ecuador | Asian | Mosquito | Partial | Sanger dideoxy sequencing |
| KF383080 | 1990 | Cote dIvoire | West African |  | Partial | Sanger dideoxy sequencing |
| OM831150 | 2017 | Ecuador | Asian | Mosquito | Partial | Sanger dideoxy sequencing |
| OM831151 | 2017 | Ecuador | Asian | Mosquito | Partial | Sanger dideoxy sequencing |
| OM831154 | 2017 | Ecuador | Asian | Mosquito | Partial | Sanger dideoxy sequencing |
| OM831153 | 2017 | Ecuador | Asian | Mosquito | Partial | Sanger dideoxy sequencing |
| OM831152 | 2017 | Ecuador | Asian | Mosquito | Partial | Sanger dideoxy sequencing |
| OK054351 | 2021 | India | Asian | Human | Complete |  |
| OM522327 | 2021 | Mexico | Asian | Human | Complete | Illumina |
| OM666891 | 2021 | India | Asian | Human | Complete |  |
| OM666892 | 2021 | India | Asian | Human | Complete | Illumina |
| OM666893 | 2021 | India | Asian | Human | Partial | Illumina |
| KF383081 | 1990 | Cote dIvoire | West African |  | Partial | Sanger dideoxy sequencing |
| ON103519 | 2021 | India | Asian | Human | Partial | Sanger dideoxy sequencing |
| MT125019 | 2017 | Thailand | Asian | Human | Partial | Sanger dideoxy sequencing |
| ON337477 | 2019 | Cuba | Asian | Human | Partial | Sanger dideoxy sequencing |
| ON337478 | 2019 | Cuba | Asian | Human | Partial | Sanger dideoxy sequencing |
| ON419458 | 2021 | India | Asian | Human | Partial | Sanger dideoxy sequencing |
| ON419459 | 2021 | India | Asian | Human | Partial | Sanger dideoxy sequencing |
| ON419460 | 2021 | India | Asian | Human | Partial | Sanger dideoxy sequencing |
| ON419461 | 2021 | India | Asian | Human | Partial | Sanger dideoxy sequencing |
| ON419463 | 2021 | India | Asian | Human | Partial | Sanger dideoxy sequencing |
| ON419462 | 2021 | India | Asian | Human | Partial | Sanger dideoxy sequencing |
| KF383082 | 1980 | Cote dIvoire | West African |  | Partial | Sanger dideoxy sequencing |
| ON419464 | 2021 | India | Asian | Human | Partial | Sanger dideoxy sequencing |
| ON419466 | 2021 | India | Asian | Human | Partial | Sanger dideoxy sequencing |
| ON419465 | 2021 | India | Asian | Human | Partial | Sanger dideoxy sequencing |
| ON419467 | 2021 | India | Asian | Human | Partial | Sanger dideoxy sequencing |
| ON419468 | 2021 | India | Asian | Human | Partial | Sanger dideoxy sequencing |
| ON419469 | 2021 | India | Asian | Human | Partial | Sanger dideoxy sequencing |
| ON419471 | 2021 | India | Asian | Human | Partial | Sanger dideoxy sequencing |
| ON419470 | 2021 | India | Asian | Human | Partial | Sanger dideoxy sequencing |
| ON419472 | 2021 | India | Asian | Human | Partial | Sanger dideoxy sequencing |
| ON419473 | 2021 | India | Asian | Human | Partial | Sanger dideoxy sequencing |
| KF383083 | 1979 | Senegal | West African |  | Partial | Sanger dideoxy sequencing |
| ON419474 | 2021 | India | Asian | Human | Partial | Sanger dideoxy sequencing |
| ON419475 | 2021 | India | Asian | Human | Partial | Sanger dideoxy sequencing |
| ON419476 | 2021 | India | Asian | Human | Partial | Sanger dideoxy sequencing |
| ON419477 | 2021 | India | Asian | Human | Partial | Sanger dideoxy sequencing |
| ON419478 | 2021 | India | Asian | Human | Partial | Sanger dideoxy sequencing |
| ON419479 | 2021 | India | Asian | Human | Partial | Sanger dideoxy sequencing |
| ON419480 | 2021 | India | Asian | Human | Partial | Sanger dideoxy sequencing |
| ON419481 | 2021 | India | Asian | Human | Partial | Sanger dideoxy sequencing |
| ON419482 | 2021 | India | Asian | Human | Partial | Sanger dideoxy sequencing |
| ON419485 | 2021 | India | Asian | Human | Partial | Sanger dideoxy sequencing |
| KF383084 | 1991 | Senegal | West African |  | Partial | Sanger dideoxy sequencing |
| ON419484 | 2021 | India | Asian | Human | Partial | Sanger dideoxy sequencing |
| ON419483 | 2021 | India | Asian | Human | Partial | Sanger dideoxy sequencing |
| ON419487 | 2021 | India | Asian | Human | Partial | Sanger dideoxy sequencing |
| ON419486 | 2021 | India | Asian | Human | Partial | Sanger dideoxy sequencing |
| ON586852 | 2016 | Mexico | Asian | Mosquito | Complete | Illumina |
| ON586853 | 2016 | Mexico | Asian | Human | Complete | Illumina |
| ON586854 | 2016 | Mexico | Asian | Mosquito | Complete | Illumina |
| ON631960 | 2006 | Thailand | Asian | Human | Partial | Sanger dideoxy sequencing |
| MZ670001 | 2021 | India | Asian | Human | Partial | Sanger dideoxy sequencing |
| KF383085 | 1969 | Senegal | West African |  | Partial | Sanger dideoxy sequencing |
| OK573257 |  | USA | Asian | Human | Partial | Illumina |
| OK573255 |  | USA | Asian | Human | Partial | Illumina |
| OK573256 |  | USA | Asian | Human | Partial | Illumina |
| OK573248 |  | USA | Asian | Human | Partial | Illumina |
| OK573252 |  | USA | Asian | Human | Partial | Illumina |
| OK573254 |  | USA | Asian | Human | Partial | Illumina |
| MW122438 | 2016 | Puerto Rico | Asian | Human | Complete | Illumina |
| KF383086 | 1999 | Cote dIvoire | East African |  | Partial | Sanger dideoxy sequencing |
| OK573253 |  | USA | Asian | Human | Partial | Illumina |
| OK573250 |  | USA | Asian | Human | Partial | Illumina |
| OK573249 |  | USA | Asian | Human | Partial | Illumina |
| OK573251 |  | USA | Asian | Human | Partial | Illumina |
| OK573247 |  | USA | Asian | Human | Partial | Illumina |
| KF383087 | 1979 | Senegal | West African |  | Partial | Sanger dideoxy sequencing |
| OK573283 |  | USA | Asian | Human | Partial | Illumina |
| OK573282 |  | USA | Asian | Human | Partial | Illumina |
| OK573293 |  | USA | Asian | Human | Partial | Illumina |
| KF383088 | 1979 | Senegal | West African |  | Partial | Sanger dideoxy sequencing |
| OK573281 |  | USA | Asian | Human | Partial | Illumina |
| OK573304 |  | USA | Asian | Human | Partial | Illumina |
| KF383089 | 2002 | Senegal | West African |  | Partial | Sanger dideoxy sequencing |
| OK573280 |  | USA | Asian | Human | Partial | Illumina |
| LC707381 | 2021 | Gabon | East African | Human | Partial |  |
| LC707380 | 2021 | Gabon | East African | Human | Partial |  |
| MZ686202 | 2021 | India | Asian | Mosquito | Partial | Sanger dideoxy sequencing |
| MZ686204 | 2021 | India | Asian | Human | Partial | Sanger dideoxy sequencing |
| MZ686203 | 2021 | India | Asian | Mosquito | Partial | Sanger dideoxy sequencing |
| OP281680 | 2006 | Thailand | Asian | Human | Complete | Sanger dideoxy sequencing |
| OM936182 | 2019 | Thailand | Asian | Human | Partial | Sanger dideoxy sequencing |
| OM936184 | 2019 | Thailand | Asian | Human | Partial | Sanger dideoxy sequencing |
| KF383090 | 2002 | Senegal | West African |  | Partial | Sanger dideoxy sequencing |
| OM936185 | 2019 | Thailand | Asian | Human | Partial | Sanger dideoxy sequencing |
| OM936183 | 2019 | Thailand | Asian | Human | Partial | Sanger dideoxy sequencing |
| OM936192 | 2019 | Thailand | Asian | Human | Partial | Sanger dideoxy sequencing |
| OM936195 | 2020 | Thailand | Asian | Human | Partial | Sanger dideoxy sequencing |
| OM936189 | 2019 | Thailand | Asian | Human | Partial | Sanger dideoxy sequencing |
| OM936188 | 2019 | Thailand | Asian | Human | Partial | Sanger dideoxy sequencing |
| OM964565 | 2019 | Thailand | Asian | Human | Complete | Sanger dideoxy sequencing |
| OM936190 | 2019 | Thailand | Asian | Human | Partial | Sanger dideoxy sequencing |
| OM964566 | 2019 | Thailand | Asian | Human | Complete | Sanger dideoxy sequencing |
| OM964568 | 2020 | Thailand | Asian | Human | Complete | Sanger dideoxy sequencing |
| KF383091 | 2001 | Senegal | West African |  | Partial | Sanger dideoxy sequencing |
| OM964567 | 2019 | Thailand | Asian | Human | Complete | Sanger dideoxy sequencing |
| OM936191 | 2019 | Thailand | Asian | Human | Partial | Sanger dideoxy sequencing |
| OM936187 | 2019 | Thailand | Asian | Human | Partial | Sanger dideoxy sequencing |
| OM936194 | 2020 | Thailand | Asian | Human | Partial | Sanger dideoxy sequencing |
| OM936193 | 2020 | Thailand | Asian | Human | Partial | Sanger dideoxy sequencing |
| OM936186 | 2019 | Thailand | Asian | Human | Partial | Sanger dideoxy sequencing |
| OP341468 | 2016 | Mexico | Asian | Mosquito | Partial | Sanger dideoxy sequencing |
| OP431394 | 2016 | Mexico | Asian | Mosquito | Partial | Sanger dideoxy sequencing |
| OP431392 | 2016 | Mexico | Asian | Mosquito | Partial | Sanger dideoxy sequencing |
| OP431393 | 2016 | Mexico | Asian | Mosquito | Partial | Sanger dideoxy sequencing |
| KF383093 | 2001 | Senegal | West African |  | Partial | Sanger dideoxy sequencing |
| KF383092 | 2001 | Senegal | West African |  | Partial | Sanger dideoxy sequencing |
| KF383094 | 2000 | Senegal | West African |  | Partial | Sanger dideoxy sequencing |
| KF383095 | 1998 | Senegal | West African |  | Partial | Sanger dideoxy sequencing |
| MW122439 | 2016 | Puerto Rico | Asian | Human | Complete | Illumina |
| KF383096 | 1998 | Senegal | West African |  | Partial | Sanger dideoxy sequencing |
| KF383097 | 1997 | Senegal | West African |  | Partial | Sanger dideoxy sequencing |
| KF383098 | 1997 | Senegal | West African |  | Partial | Sanger dideoxy sequencing |
| KF383099 | 1997 | Senegal | West African |  | Partial | Sanger dideoxy sequencing |
| KF383100 | 1997 | Senegal | West African |  | Partial | Sanger dideoxy sequencing |
| KF383101 | 1997 | Senegal | West African |  | Partial | Sanger dideoxy sequencing |
| KF383102 | 1997 | Senegal | West African |  | Partial | Sanger dideoxy sequencing |
| KF383103 | 1999 | Cote dIvoire | East African |  | Partial | Sanger dideoxy sequencing |
| KF383104 | 1999 | Cote dIvoire | East African |  | Partial | Sanger dideoxy sequencing |
| KF383105 | 1996 | Cote dIvoire | East African |  | Partial | Sanger dideoxy sequencing |
| MW122440 | 2016 | Puerto Rico | Asian | Human | Complete | Illumina |
| KF383106 | 1990 | Cote dIvoire | West African |  | Partial | Sanger dideoxy sequencing |
| KF383108 | 1990 | Cote dIvoire | West African |  | Partial | Sanger dideoxy sequencing |
| KF383109 | 1981 | Burkina Faso | West African |  | Partial | Sanger dideoxy sequencing |
| KF383110 | 1990 | Cote dIvoire | West African |  | Partial | Sanger dideoxy sequencing |
| KF383111 | 1990 | Cote dIvoire | West African |  | Partial | Sanger dideoxy sequencing |
| KF383112 | 1990 | Cote dIvoire | West African |  | Partial | Sanger dideoxy sequencing |
| KF383113 | 1980 | Cote dIvoire | West African |  | Partial | Sanger dideoxy sequencing |
| KF383114 | 1979 | Senegal | West African |  | Partial | Sanger dideoxy sequencing |
| MW122441 | 2016 | Puerto Rico | Asian | Human | Complete | Illumina |
| KU922923 | 2016 | Mexico | Asian | Human | Complete | IonTorrent |
| KU922960 | 2016 | Mexico | Asian | Human | Complete | IonTorrent |
| KU926309 | 2016 | Brazil | Asian | Human | Complete | Illumina |
| KU926310 | 2016 | Brazil | Asian | Human | Complete | Illumina |
| MW122442 | 2016 | Puerto Rico | Asian | Human | Complete | Illumina |
| KU926323 | 2016 | Israel | Asian | Human | Partial | Sanger dideoxy sequencing |
| KU926324 | 2016 | Israel | Asian | Human | Partial | Sanger dideoxy sequencing |
| KU926325 | 2016 | Israel | Asian | Human | Partial | Sanger dideoxy sequencing |
| KU926326 | 2015 | Israel | Asian | Human | Partial | Sanger dideoxy sequencing |
| KU937936 | 2016 | Suriname | Asian | Human | Complete | Sanger dideoxy sequencing; 454 |
| KU940224 | 2015 | Brazil | Asian | Human | Partial | Illumina |
| KU940227 | 2015 | Brazil | Asian | Human | Partial | Illumina |
| KU940228 | 2015 | Brazil | Asian | Human | Partial | Illumina |
| KU954085 | 2016 | Colombia | Asian | Human | Partial | Sanger dideoxy sequencing |
| KU955589 | 2016 | China | Asian | Human | Complete | Sanger dideoxy sequencing |
| MW122443 | 2016 | Puerto Rico | Asian | Human | Complete | Illumina |
| KU955590 | 2016 | China | Asian | Human | Complete | Sanger dideoxy sequencing |
| KU955591 | 1984 | Senegal | West African | Mosquito | Complete | Illumina |
| KU955592 | 1984 | Senegal | West African | Mosquito | Complete | Illumina |
| KU955593 | 2010 | Cambodia | Asian | Human | Complete | Illumina |
| KU955594 | 1947 | Uganda | East African | Animal | Complete | Illumina |
| KU955595 | 1984 | Senegal | West African | Mosquito | Complete | Illumina |
| MG494697 | 2016 | Mexico | Asian | Human | Complete | Illumina |
| MG548660 | 2016 | Thailand | Asian | Human | Complete | Illumina; IonTorrent; ONT |
| MG548661 | 2016 | Thailand | Asian | Human | Complete | Illumina; IonTorrent; ONT |
| KU963573 | 1947 | Uganda | East African | Animal | Complete | Illumina |
| MW122444 | 2016 | Puerto Rico | Asian | Human | Complete | Illumina |
| KU963574 | 1968 | Nigeria | West African | Human | Complete | Illumina |
| KU963796 | 2016 | China | Asian | Human | Complete | Sanger dideoxy sequencing |
| KU978616 | 2016 | Russia | Asian | Human | Partial | Sanger dideoxy sequencing |
| KU985087 | 2015 | Mexico | Asian | Human | Partial | Sanger dideoxy sequencing |
| KU985088 | 2015 | Mexico | Asian | Human | Partial | Sanger dideoxy sequencing |
| KU991811 | 2016 | Italy | Asian | Human | Complete | Sanger dideoxy sequencing |
| KU997667 | 2016 | China | Asian | Human | Complete | IonTorrent |
| KX013000 | 2016 | China | Asian | Human | Complete | IonTorrent |
| KX051560 | 2013 | Thailand | Asian | Human | Complete | Illumina |
| KX051561 | 2013 | Thailand | Asian | Human | Complete | Illumina |
| MW122445 | 2016 | Puerto Rico | Asian | Human | Complete | Illumina |
| KX051563 | 2016 | USA | Asian | Human | Complete | Sanger dideoxy sequencing |
| KX051562 | 2015 | Thailand | Asian | Human | Complete | Illumina |
| KX056898 | 2016 | China | Asian | Human | Complete | Sanger dideoxy sequencing |
| KX059013 | 2014 | Haiti | Asian | Human | Partial | Sanger dideoxy sequencing |
| KX059014 | 2014 | Haiti | Asian | Human | Partial | Sanger dideoxy sequencing |
| MG595216 | 2016 | Mexico | Asian | Human | Complete | Illumina |
| KX062044 | 2014 | Haiti | Asian | Human | Partial | Sanger dideoxy sequencing |
| KX062045 | 2014 | Haiti | Asian | Human | Partial | Sanger dideoxy sequencing |
| MG645981 | 2006 | Thailand | Asian | Human | Complete | Sanger dideoxy sequencing |
| MG674718 | 2016 | China | Asian | Human | Complete | Sanger dideoxy sequencing |
| MW122446 | 2016 | Puerto Rico | Asian | Human | Complete | Illumina |
| MG674719 | 2016 | China | Asian | Human | Complete | Sanger dideoxy sequencing |
| KX087102 | 2015 | Colombia | Asian | Human | Complete | Illumina |
| KX087101 | 2015 | Puerto Rico | Asian | Human | Complete | Illumina |
| KX101060 | 2015 | Brazil | Asian | Human | Partial | Illumina |
| KX101061 | 2015 | Brazil | Asian | Human | Partial | Illumina |
| KX101062 | 2015 | Brazil | Asian | Human | Partial | Illumina |
| KX101063 | 2015 | Brazil | Asian | Human | Partial | Illumina |
| KX101064 | 2015 | Brazil | Asian | Human | Partial | Illumina |
| KX101065 | 2016 | Brazil | Asian | Human | Partial | Illumina |
| KX101066 | 2015 | Brazil | Asian | Human | Partial | Illumina |
| MW122447 | 2016 | Puerto Rico | Asian | Human | Complete | Illumina |
| KX101067 | 2015 | Brazil | Asian | Human | Partial | Illumina |
| KJ680134 | 2013 | French Polynesia | Asian | Human | Partial |  |
| KJ680135 | 2014 | French Polynesia | Asian | Human | Partial |  |
| KJ776791 | 2013 | French Polynesia | Asian | Human | Complete | IonTorrent |
| KM078929 | 2014 | Chile | Asian | Human | Partial |  |
| KM078930 | 2014 | Chile | Asian | Human | Partial |  |
| MW122448 | 2016 | Puerto Rico | Asian | Human | Complete | Illumina |
| KM078931 | 2014 | Chile | Asian | Human | Partial |  |
| KM078934 | 2014 | Chile | Asian | Human | Partial |  |
| KM078935 | 2014 | Chile | Asian | Human | Partial |  |
| KM078936 | 2014 | Chile | Asian | Human | Partial |  |
| KM078937 | 2014 | Chile | Asian | Human | Partial |  |
| KM078938 | 2014 | Chile | Asian | Human | Partial |  |
| KM078939 | 2014 | Chile | Asian | Human | Partial |  |
| KM078940 | 2014 | Chile | Asian | Human | Partial |  |
| KM078942 | 2014 | Chile | Asian | Human | Partial |  |
| KM078943 | 2014 | Chile | Asian | Human | Partial |  |
| MW122449 | 2016 | Puerto Rico | Asian | Human | Complete | Illumina |
| KM078944 | 2014 | Chile | Asian | Human | Partial |  |
| KM078948 | 2014 | Chile | Asian | Human | Partial |  |
| KM078949 | 2014 | Chile | Asian | Human | Partial |  |
| KM078950 | 2014 | Chile | Asian | Human | Partial |  |
| KM078951 | 2014 | Chile | Asian | Human | Partial |  |
| KM078952 | 2014 | Chile | Asian | Human | Partial |  |
| KM078953 | 2014 | Chile | Asian | Human | Partial |  |
| KM078954 | 2014 | Chile | Asian | Human | Partial |  |
| KM078959 | 2014 | Chile | Asian | Human | Partial |  |
| MW122450 | 2017 | Puerto Rico | Asian | Human | Complete | Illumina |
| KM078960 | 2014 | Chile | Asian | Human | Partial |  |
| KM078962 | 2014 | Chile | Asian | Human | Partial |  |
| KM078967 | 2014 | Chile | Asian | Human | Partial |  |
| KM078969 | 2014 | Chile | Asian | Human | Partial |  |
| KM078972 | 2014 | Chile | Asian | Human | Partial |  |
| KM078973 | 2014 | Chile | Asian | Human | Partial |  |
| KM078974 | 2014 | Chile | Asian | Human | Partial |  |
| KM078975 | 2014 | Chile | Asian | Human | Partial |  |
| KM078955 | 2014 | Chile | Asian | Human | Partial |  |
| KM078978 | 2014 | Chile | Asian | Human | Partial |  |
| MW122451 | 2017 | Puerto Rico | Asian | Human | Complete | Illumina |
| KM078979 | 2014 | Chile | Asian | Human | Partial |  |
| KM212963 | 2014 | New Caledonia | Asian | Human | Partial | Sanger dideoxy sequencing |
| KM212966 | 2013 | New Caledonia | Asian | Human | Partial | Sanger dideoxy sequencing |
| KM212967 | 2013 | New Caledonia | Asian | Human | Partial | Sanger dideoxy sequencing |
| KF993678 | 2013 | Canada | Asian | Human | Partial | Sanger dideoxy sequencing |
| EU545988 | 2007 | Micronesia | Asian | Human | Complete |  |
| JN860885 | 2010 | Cambodia | Asian | Human | Partial |  |
| KM851039 | 2014 | Thailand | Asian | Human | Partial | Illumina |
| KJ461621 | 2013 | Norway | Asian | Human | Partial |  |
| KP099609 | 2014 | Cook Islands | Asian | Human | Partial |  |
| MW122452 | 2017 | Puerto Rico | Asian | Human | Complete | Illumina |
| KP099610 | 2014 | Cook Islands | Asian | Human | Partial |  |
| KJ579441 | 2013 | French Polynesia | Asian | Human | Partial |  |
| KJ579442 | 2013 | French Polynesia | Asian | Human | Partial |  |
| KJ634273 | 2014 | Cook Islands | Asian | Human | Partial | Sanger dideoxy sequencing |
| KY075932 | 2016 | Martinique | Asian | Human | Complete | Illumina |
| KY075933 | 2016 | USA | Asian | Human | Complete | Illumina |
| KY075934 | 2016 | USA | Asian | Human | Complete | Illumina |
| KY075935 | 2016 | USA | Asian | Human | Complete | Illumina |
| KY075936 | 2016 | USA | Asian | Human | Complete | Illumina |
| KY075937 | 2016 | USA | Asian | Mosquito | Complete | Illumina |
| MW122453 | 2016 | Puerto Rico | Asian | Human | Complete | Illumina |
| KY075938 | 2016 | USA | Asian | Mosquito | Complete | Illumina |
| KY075939 | 2016 | USA | Asian | Mosquito | Complete | Illumina |
| KY120348 | 2016 | Mexico | Asian | Human | Complete | Illumina |
| KY120349 | 2016 | Mexico | Asian | Human | Complete | Illumina |
| KY120352 | 2016 | South Korea | Asian | Human | Complete | Sanger dideoxy sequencing |
| KY120353 | 2016 | South Korea | Asian | Human | Complete | Sanger dideoxy sequencing |
| KY126345 | 2016 | Taiwan | Asian | Human | Partial | Sanger dideoxy sequencing |
| KY126346 | 2016 | Taiwan | Asian | Human | Partial | Sanger dideoxy sequencing |
| KY126347 | 2016 | Taiwan | Asian | Human | Partial | Sanger dideoxy sequencing |
| KY126348 | 2016 | Taiwan | Asian | Human | Partial | Sanger dideoxy sequencing |
| MW122454 | 2016 | Puerto Rico | Asian | Human | Complete | Illumina |
| KY126349 | 2016 | Taiwan | Asian | Human | Partial | Sanger dideoxy sequencing |
| KY126350 | 2016 | Taiwan | Asian | Human | Partial | Sanger dideoxy sequencing |
| KY126351 | 2016 | Taiwan | Asian | Human | Complete | Sanger dideoxy sequencing |
| KY131441 | 2013 | Viet Nam | Asian | Human | Partial | Illumina |
| KY131442 | 2013 | Viet Nam | Asian | Human | Partial | Illumina |
| MH179341 | 2015 | Colombia | Asian | Mosquito | Complete | Sanger dideoxy sequencing |
| MF036115 | 2016 | China | Asian | Human | Complete | Sanger dideoxy sequencing |
| MN566104 | 2016 | Haiti | Asian | Human | Complete | Sanger dideoxy sequencing |
| MN566105 | 2016 | Haiti | Asian | Human | Complete | Sanger dideoxy sequencing |
| MN566106 | 2016 | Haiti | Asian | Human | Complete | Sanger dideoxy sequencing |
| MW122455 | 2017 | Puerto Rico | Asian | Human | Complete | Illumina |
| MN566107 | 2016 | Haiti | Asian | Human | Complete | Sanger dideoxy sequencing |
| MN566108 | 2016 | Haiti | Asian | Human | Complete | Sanger dideoxy sequencing |
| MN577543 | 2014 | Haiti | Asian | Human | Complete | Sanger dideoxy sequencing |
| MN577544 | 2016 | Haiti | Asian | Human | Complete | Sanger dideoxy sequencing |
| MN577550 | 2016 | Nicaragua | Asian | Human | Complete | IonTorrent |
| LC171327 | 2016 | Japan | Asian | Human | Partial |  |
| LC190723 | 2016 | Japan | Asian | Human | Complete |  |
| LC191864 | 2016 | Japan | Asian | Human | Complete |  |
| MF048802 | 2016 | Brazil | Asian | Human | Partial | Sanger dideoxy sequencing |
| MF048803 | 2016 | Brazil | Asian | Human | Partial | Sanger dideoxy sequencing |
| MW165880 | 2016 | Virgin Islands | Asian | Human | Complete | Illumina; Oxford Nanopore |
| MF048804 | 2016 | Brazil | Asian | Human | Partial | Sanger dideoxy sequencing |
| MF048805 | 2016 | Brazil | Asian | Human | Partial | Sanger dideoxy sequencing |
| MF048806 | 2016 | Brazil | Asian | Human | Partial | Sanger dideoxy sequencing |
| MF048807 | 2016 | Brazil | Asian | Human | Partial | Sanger dideoxy sequencing |
| MF048808 | 2016 | Brazil | Asian | Human | Partial | Sanger dideoxy sequencing |
| MF073357 | 2016 | Brazil | Asian | Human | Complete | Sanger dideoxy sequencing |
| MF073358 | 2015 | Brazil | Asian | Human | Complete | Illumina |
| MF073359 | 2015 | Brazil | Asian | Human | Complete | Sanger dideoxy sequencing |
| MN600713 | 2019 | Malaysia | Asian | Human | Partial | Sanger dideoxy sequencing |
| MF077458 | 2016 | Brazil | Asian | Human | Partial | Sanger dideoxy sequencing |
| MW165881 | 2016 | Virgin Islands | Asian | Human | Complete | Illumina; Oxford Nanopore |
| MN611472 | 2019 | China | Asian | Human | Complete | Sanger dideoxy sequencing |
| MF077459 | 2016 | Brazil | Asian | Human | Partial | Sanger dideoxy sequencing |
| MF077460 | 2016 | Brazil | Asian | Human | Partial | Sanger dideoxy sequencing |
| MF077461 | 2016 | Brazil | Asian | Human | Partial | Sanger dideoxy sequencing |
| MF077462 | 2016 | Brazil | Asian | Human | Partial | Sanger dideoxy sequencing |
| MF077463 | 2016 | Brazil | Asian | Human | Partial | Sanger dideoxy sequencing |
| MF098764 | 2016 | Russia | Asian | Human | Partial | Sanger dideoxy sequencing |
| MF098765 | 2016 | Russia | Asian | Human | Partial | Sanger dideoxy sequencing |
| MF098766 | 2016 | Russia | Asian | Human | Complete | Sanger dideoxy sequencing |
| MF098767 | 2016 | Russia | Asian | Human | Partial | Sanger dideoxy sequencing |
| MW165882 | 2016 | Virgin Islands | Asian | Human | Complete | Illumina; Oxford Nanopore |
| MF098768 | 2016 | Russia | Asian | Human | Complete | Sanger dideoxy sequencing |
| MF098769 | 2016 | Russia | Asian | Human | Complete | Sanger dideoxy sequencing |
| MF098770 | 2016 | Russia | Asian | Human | Partial | Sanger dideoxy sequencing |
| MF098771 | 2017 | Russia | Asian | Human | Complete | Sanger dideoxy sequencing |
| MF099651 | 2016 | China | Asian | Mosquito | Partial | Sanger dideoxy sequencing |
| LC217862 | 2016 | Malaysia | Asian | Human | Partial |  |
| LC218742 | 2016 | Japan | Asian | Human | Partial |  |
| LC219720 | 2016 | Japan | Asian | Human | Complete |  |
| LC229347 | 2016 | Malaysia | Asian | Human | Partial |  |
| MW165883 | 2016 | Virgin Islands | Asian | Human | Complete | Illumina; Oxford Nanopore |
| LC322991 | 2016 | Malaysia | Asian | Human | Partial |  |
| LC322992 | 2016 | Malaysia | Asian | Human | Partial |  |
| LC331561 | 2016 | Japan | Asian | Human | Partial |  |
| LC369584 | 2017 | Japan | Asian | Human | Complete |  |
| MF159531 | 2017 | USA | Asian | Human | Complete | Illumina |
| MF167360 | 2016 | China | Asian | Human | Complete | Sanger dideoxy sequencing |
| MF173409 | 2016 | India | Asian | Human | Partial | Sanger dideoxy sequencing |
| MF173410 | 2016 | India | Asian | Human | Partial | Sanger dideoxy sequencing |
| MF173411 | 2016 | India | Asian | Human | Partial | Sanger dideoxy sequencing |
| KY241671 | 2016 | Singapore | Asian | Human | Complete | Illumina |
| MW165884 | 2016 | Virgin Islands | Asian | Human | Complete | Illumina; Oxford Nanopore |
| KY241673 | 2016 | Singapore | Asian | Human | Complete | Illumina |
| KY241674 | 2016 | Singapore | Asian | Human | Complete | Illumina |
| KY241675 | 2016 | Singapore | Asian | Human | Complete | Illumina |
| KY241678 | 2016 | Singapore | Asian | Human | Complete | Illumina |
| KY241679 | 2016 | Singapore | Asian | Human | Complete | Illumina |
| KY241680 | 2016 | Singapore | Asian | Human | Complete | Illumina |
| KY241681 | 2016 | Singapore | Asian | Human | Complete | Illumina |
| KY241682 | 2016 | Singapore | Asian | Human | Complete | Illumina |
| KY241683 | 2016 | Singapore | Asian | Human | Complete | Illumina |
| NC_012532 |  | Uganda | East African | Animal | Complete |  |
| KY241684 | 2016 | Singapore | Asian | Human | Complete | Illumina |
| KY241685 | 2016 | Singapore | Asian | Human | Complete | Illumina |
| KY241677 | 2016 | Singapore | Asian | Human | Complete | Illumina |
| KY241686 | 2016 | Singapore | Asian | Human | Complete | Illumina |
| KY241687 | 2016 | Singapore | Asian | Human | Complete | Illumina |
| KY241688 | 2016 | Singapore | Asian | Human | Complete | Illumina |
| KY241689 | 2016 | Singapore | Asian | Human | Complete | Illumina |
| KY241690 | 2016 | Singapore | Asian | Human | Complete | Illumina |
| KY241691 | 2016 | Singapore | Asian | Human | Complete | Illumina |
| KY241692 | 2016 | Singapore | Asian | Human | Complete | Illumina |
| NC_035889 | 2015 | Brazil | Asian | Human | Complete |  |
| KY241693 | 2016 | Singapore | Asian | Human | Complete | Illumina |
| KY241694 | 2016 | Singapore | Asian | Human | Complete | Illumina |
| KY241695 | 2016 | Singapore | Asian | Human | Complete | Illumina |
| KY241696 | 2016 | Singapore | Asian | Human | Complete | Illumina |
| KY241697 | 2016 | Singapore | Asian | Human | Complete | Illumina |
| KY241698 | 2016 | Singapore | Asian | Human | Complete | Illumina |
| KY241699 | 2016 | Singapore | Asian | Human | Complete | Illumina |
| KY241700 | 2016 | Singapore | Asian | Human | Complete | Illumina |
| KY241701 | 2016 | Singapore | Asian | Human | Complete | Illumina |
| KY241702 | 2016 | Singapore | Asian | Human | Complete | Illumina |
| KU179098 | 2014 | Indonesia | Asian | Human | Complete | Sanger dideoxy sequencing |
| KY241703 | 2016 | Singapore | Asian | Human | Complete | Illumina |
| KY241704 | 2016 | Singapore | Asian | Human | Complete | Illumina |
| KY241705 | 2016 | Singapore | Asian | Human | Complete | Illumina |
| KY241706 | 2016 | Singapore | Asian | Human | Complete | Illumina |
| KY241707 | 2016 | Singapore | Asian | Human | Complete | Illumina |
| KY241708 | 2016 | Singapore | Asian | Human | Complete | Illumina |
| KY241709 | 2016 | Singapore | Asian | Human | Complete | Illumina |
| KY241710 | 2016 | Singapore | Asian | Human | Complete | Illumina |
| KY241711 | 2016 | Singapore | Asian | Human | Complete | Illumina |
| KY241712 | 2016 | Singapore | Asian | Human | Complete | Illumina |
| KU232288 | 2015 | Brazil | Asian | Human | Partial | Sanger dideoxy sequencing |
| KY241713 | 2016 | Singapore | Asian | Human | Complete | Illumina |
| KY241714 | 2016 | Singapore | Asian | Human | Complete | Illumina |
| KY241715 | 2016 | Singapore | Asian | Human | Complete | Illumina |
| KY241716 | 2016 | Singapore | Asian | Human | Complete | Illumina |
| KY241717 | 2016 | Singapore | Asian | Human | Complete | Illumina |
| KY241718 | 2016 | Singapore | Asian | Human | Complete | Illumina |
| KY241719 | 2016 | Singapore | Asian | Human | Complete | Illumina |
| KY241721 | 2016 | Singapore | Asian | Human | Complete | Illumina |
| KY241722 | 2016 | Singapore | Asian | Human | Complete | Illumina |
| KU232290 | 2015 | Brazil | Asian | Human | Partial | Sanger dideoxy sequencing |
| KY241723 | 2016 | Singapore | Asian | Human | Complete | Illumina |
| KY241725 | 2016 | Singapore | Asian | Human | Complete | Illumina |
| KY241726 | 2016 | Singapore | Asian | Human | Complete | Illumina |
| KY241727 | 2016 | Singapore | Asian | Human | Complete | Illumina |
| KY241728 | 2016 | Singapore | Asian | Human | Complete | Illumina |
| KY241729 | 2016 | Singapore | Asian | Human | Complete | Illumina |
| KY241730 | 2016 | Singapore | Asian | Human | Complete | Illumina |
| KY241731 | 2016 | Singapore | Asian | Human | Complete | Illumina |
| KY241732 | 2016 | Singapore | Asian | Human | Complete | Illumina |
| KU232289 | 2015 | Brazil | Asian | Human | Partial | Sanger dideoxy sequencing |
| KY241733 | 2016 | Singapore | Asian | Human | Complete | Illumina |
| KY241734 | 2016 | Singapore | Asian | Human | Complete | Illumina |
| KY241735 | 2016 | Singapore | Asian | Human | Complete | Illumina |
| KY241736 | 2016 | Singapore | Asian | Human | Complete | Illumina |
| KY241737 | 2016 | Singapore | Asian | Human | Complete | Illumina |
| KY241738 | 2016 | Singapore | Asian | Human | Complete | Illumina |
| KY241739 | 2016 | Singapore | Asian | Human | Complete | Illumina |
| KY241740 | 2016 | Singapore | Asian | Human | Complete | Illumina |
| KY241741 | 2016 | Singapore | Asian | Human | Complete | Illumina |
| KY241742 | 2016 | Singapore | Asian | Human | Complete | Illumina |
| KU232293 | 2015 | Brazil | Asian | Human | Partial | Sanger dideoxy sequencing |
| KY241743 | 2016 | Singapore | Asian | Human | Complete | Illumina |
| KY241744 | 2016 | Singapore | Asian | Human | Complete | Illumina |
| KY241745 | 2016 | Singapore | Asian | Human | Complete | Illumina |
| KY241746 | 2016 | Singapore | Asian | Human | Complete | Illumina |
| KY241747 | 2016 | Singapore | Asian | Human | Complete | Illumina |
| KY241748 | 2016 | Singapore | Asian | Human | Complete | Illumina |
| KY241749 | 2016 | Singapore | Asian | Human | Complete | Illumina |
| KY241750 | 2016 | Singapore | Asian | Human | Complete | Illumina |
| KY241751 | 2016 | Singapore | Asian | Human | Complete | Illumina |
| KY241752 | 2016 | Singapore | Asian | Human | Complete | Illumina |
| KU232291 | 2015 | Brazil | Asian | Human | Partial | Sanger dideoxy sequencing |
| KY241753 | 2016 | Singapore | Asian | Human | Complete | Illumina |
| KY241754 | 2016 | Singapore | Asian | Human | Complete | Illumina |
| KY241755 | 2016 | Singapore | Asian | Human | Complete | Illumina |
| KY241756 | 2016 | Singapore | Asian | Human | Complete | Illumina |
| KY241757 | 2016 | Singapore | Asian | Human | Complete | Illumina |
| KY241758 | 2016 | Singapore | Asian | Human | Complete | Illumina |
| KY241759 | 2016 | Singapore | Asian | Human | Complete | Illumina |
| KY241760 | 2016 | Singapore | Asian | Human | Complete | Illumina |
| KY241761 | 2016 | Singapore | Asian | Human | Complete | Illumina |
| KY241762 | 2016 | Singapore | Asian | Human | Complete | Illumina |
| KU232292 | 2015 | Brazil | Asian | Human | Partial | Sanger dideoxy sequencing |
| KY241763 | 2016 | Singapore | Asian | Human | Complete | Illumina |
| KY241764 | 2016 | Singapore | Asian | Human | Complete | Illumina |
| KY241765 | 2016 | Singapore | Asian | Human | Complete | Illumina |
| KY241766 | 2016 | Singapore | Asian | Human | Complete | Illumina |
| KY241767 | 2016 | Singapore | Asian | Human | Complete | Illumina |
| KY241769 | 2016 | Singapore | Asian | Human | Complete | Illumina |
| KY241770 | 2016 | Singapore | Asian | Human | Complete | Illumina |
| KY241771 | 2016 | Singapore | Asian | Human | Complete | Illumina |
| KY241772 | 2016 | Singapore | Asian | Human | Complete | Illumina |
| KU232294 | 2015 | Brazil | Asian | Human | Partial | Sanger dideoxy sequencing |
| KY241773 | 2016 | Singapore | Asian | Human | Complete | Illumina |
| KY241774 | 2016 | Singapore | Asian | Mosquito | Complete | Illumina |
| KY241775 | 2016 | Singapore | Asian | Mosquito | Complete | Illumina |
| KY241776 | 2016 | Singapore | Asian | Mosquito | Complete | Illumina |
| KY241777 | 2016 | Singapore | Asian | Mosquito | Complete | Illumina |
| KY241778 | 2016 | Singapore | Asian | Mosquito | Complete | Illumina |
| KY241779 | 2016 | Singapore | Asian | Mosquito | Complete | Illumina |
| KY241780 | 2016 | Singapore | Asian | Mosquito | Complete | Illumina |
| KY241781 | 2016 | Singapore | Asian | Mosquito | Complete | Illumina |
| KY241782 | 2016 | Singapore | Asian | Mosquito | Complete | Illumina |
| KU232296 | 2015 | Brazil | Asian | Human | Partial | Sanger dideoxy sequencing |
| KY241783 | 2016 | Singapore | Asian | Mosquito | Complete | Illumina |
| KY241784 | 2016 | Singapore | Asian | Mosquito | Complete | Illumina |
| KY241785 | 2016 | Singapore | Asian | Mosquito | Complete | Illumina |
| KY241786 | 2016 | Singapore | Asian | Mosquito | Complete | Illumina |
| KY241787 | 2016 | Singapore | Asian | Mosquito | Complete | Illumina |
| KY241788 | 2016 | Singapore | Asian | Human | Complete | Illumina |
| MH249062 | 2016 | Brazil | Asian | Human | Partial | Sanger dideoxy sequencing |
| MH249063 | 2016 | Brazil | Asian | Human | Partial | Sanger dideoxy sequencing |
| MH249064 | 2016 | Brazil | Asian | Human | Partial | Sanger dideoxy sequencing |
| MH249065 | 2016 | Brazil | Asian | Human | Partial | Sanger dideoxy sequencing |
| KU232295 | 2015 | Brazil | Asian | Human | Partial | Sanger dideoxy sequencing |
| MH249066 | 2016 | Brazil | Asian | Human | Partial | Sanger dideoxy sequencing |
| MH249067 | 2016 | Brazil | Asian | Human | Partial | Sanger dideoxy sequencing |
| MH252914 | 2017 | Brazil | Asian | Mosquito | Partial | Sanger dideoxy sequencing |
| MH252915 | 2017 | Brazil | Asian | Mosquito | Partial | Sanger dideoxy sequencing |
| MH252918 | 2017 | Brazil | Asian | Mosquito | Partial | Sanger dideoxy sequencing |
| MH252919 | 2017 | Brazil | Asian | Mosquito | Partial | Sanger dideoxy sequencing |
| MH252920 | 2017 | Brazil | Asian | Mosquito | Partial | Sanger dideoxy sequencing |
| MH255601 | 2016 | Singapore | Asian | Human | Complete | Sanger dideoxy sequencing |
| MH255606 | 2016 | Grenada | Asian | Animal | Partial | Sanger dideoxy sequencing |
| KY272987 | 2016 | Thailand | Asian | Human | Complete | IonTorrent |
| KU232297 | 2015 | Brazil | Asian | Human | Partial | Sanger dideoxy sequencing |
| KY272991 | 2016 | Brazil | Asian | Human | Complete | Sanger dideoxy sequencing |
| KY288905 | 1962 | Uganda | East African | Mosquito | Complete | Illumina |
| KY293644 | 2016 | New Zealand | Asian | Human | Partial | Sanger dideoxy sequencing |
| KY293645 | 2016 | New Zealand | Asian | Human | Partial | Sanger dideoxy sequencing |
| MH306200 | 2015 | Brazil | Asian | Human | Partial | Sanger dideoxy sequencing |
| MH306201 | 2015 | Brazil | Asian | Human | Partial | Sanger dideoxy sequencing |
| MH306202 | 2015 | Brazil | Asian | Human | Partial | Sanger dideoxy sequencing |
| MH306203 | 2016 | Brazil | Asian | Human | Partial | Sanger dideoxy sequencing |
| MH306204 | 2015 | Brazil | Asian | Human | Partial | Sanger dideoxy sequencing |
| MH306209 | 2017 | Thailand | Asian | Mosquito | Partial | Sanger dideoxy sequencing |
| KU232298 | 2015 | Brazil | Asian | Human | Partial | Sanger dideoxy sequencing |
| MH306210 | 2017 | Thailand | Asian | Mosquito | Partial | Sanger dideoxy sequencing |
| MH306211 | 2017 | Thailand | Asian | Mosquito | Partial | Sanger dideoxy sequencing |
| MH306212 | 2017 | Thailand | Asian | Mosquito | Partial | Sanger dideoxy sequencing |
| MH306213 | 2017 | Thailand | Asian | Mosquito | Partial | Sanger dideoxy sequencing |
| MH306214 | 2017 | Thailand | Asian | Mosquito | Partial | Sanger dideoxy sequencing |
| KY307811 | 2016 | Brazil | Asian | Animal | Partial | Sanger dideoxy sequencing |
| MH306215 | 2017 | Thailand | Asian | Mosquito | Partial | Sanger dideoxy sequencing |
| MH306216 | 2017 | Thailand | Asian | Mosquito | Partial | Sanger dideoxy sequencing |
| KY317936 | 2016 | Colombia | Asian | Human | Complete | Illumina |
| KY317937 | 2016 | Colombia | Asian | Human | Complete | Illumina |
| KU232300 | 2015 | Brazil | Asian | Human | Partial | Sanger dideoxy sequencing |
| KY317938 | 2016 | Colombia | Asian | Human | Complete | Illumina |
| KY317939 | 2016 | Colombia | Asian | Human | Complete | Illumina |
| KY317940 | 2016 | Colombia | Asian | Human | Complete | Illumina |
| KY325464 | 2016 | USA | Asian | Human | Complete |  |
| KY325465 | 2016 | USA | Asian | Human | Complete |  |
| KY325466 | 2016 | USA | Asian | Human | Complete |  |
| KY325467 | 2016 | USA | Asian | Human | Complete |  |
| KY325468 | 2016 | USA | Asian | Human | Complete |  |
| KY325470 | 2016 | USA | Asian | Human | Partial |  |
| KY325469 | 2016 | USA | Asian | Human | Complete |  |
| KU232299 | 2015 | Brazil | Asian | Human | Partial | Sanger dideoxy sequencing |
| KY325471 | 2016 | USA | Asian | Human | Complete |  |
| KY325472 | 2016 | USA | Asian | Human | Complete |  |
| KY325473 | 2016 | USA | Asian | Human | Complete |  |
| KY325474 | 2016 | USA | Asian | Human | Partial |  |
| KY325475 | 2016 | USA | Asian | Human | Partial |  |
| KY325476 | 2016 | USA | Asian | Human | Complete |  |
| KY325477 | 2016 | USA | Asian | Human | Complete |  |
| KY325478 | 2016 | USA | Asian | Human | Partial |  |
| KY325479 | 2016 | USA | Asian | Human | Complete |  |
| KY325480 | 2016 | USA | Asian | Human | Complete |  |
| KU232301 | 2015 | Brazil | Asian | Human | Partial | Sanger dideoxy sequencing |
| KY325481 | 2016 | USA | Asian | Human | Complete |  |
| KY325482 | 2016 | USA | Asian | Human | Complete |  |
| KY325483 | 2016 | USA | Asian | Human | Complete |  |
| KY328289 | 2016 | Honduras | Asian | Human | Complete | Illumina |
| KY328290 | 2016 | China | Asian | Human | Complete | Sanger dideoxy sequencing |
| MN755616 | 1984 | Senegal | West African | Laboratory | Partial | Sanger dideoxy sequencing |
| MN755617 | 1968 | Nigeria | West African | Laboratory | Partial | Sanger dideoxy sequencing |
| MN755618 | 2016 | Mexico | Asian | Laboratory | Partial | Sanger dideoxy sequencing |
| MN755619 | 2013 | French Polynesia | Asian | Laboratory | Partial | Sanger dideoxy sequencing |
| MN755620 | 2015 | Colombia | Asian | Laboratory | Partial | Sanger dideoxy sequencing |
| KU312312 | 2015 | Suriname | Asian | Human | Complete | Sanger dideoxy sequencing |
| MN755621 | 2010 | Cambodia | Asian | Laboratory | Partial | Sanger dideoxy sequencing |
| MN755623 | 2015 | Puerto Rico | Asian | Laboratory | Partial | Sanger dideoxy sequencing |
| MN755624 | 1947 | Uganda | East African | Laboratory | Partial | Sanger dideoxy sequencing |
| MN755625 | 1966 | Malaysia | Asian | Laboratory | Partial | Sanger dideoxy sequencing |
| MN755626 | 2013 | Thailand | Asian | Laboratory | Partial | Sanger dideoxy sequencing |
| MN755622 | 2015 | Brazil | Asian | Laboratory | Partial | Sanger dideoxy sequencing |
| KY348640 | 2016 | Suriname | Asian | Human | Complete | Pacific Biosciences; Sanger dideoxy |
| KY348860 | 1984 | Senegal | West African | Mosquito | Complete | Illumina |
| KY354186 | 2015 | Brazil | Asian | Mosquito | Partial | Illumina |
| KY354187 | 2016 | Brazil | Asian | Mosquito | Partial | Illumina |
| KU312313 | 2015 | Suriname | Asian | Human | Partial | Sanger dideoxy sequencing |
| KY379148 | 2016 | China | Asian | Human | Partial | Sanger dideoxy sequencing |
| MH368551 | 2016 | Cambodia | Asian |  | Complete | Illumina |
| MH379777 | 2016 | Brazil | Asian | Human | Partial | Sanger dideoxy sequencing |
| MH379778 | 2016 | Brazil | Asian | Human | Partial | Sanger dideoxy sequencing |
| MH379779 | 2016 | Brazil | Asian | Human | Partial | Sanger dideoxy sequencing |
| MH379780 | 2016 | Brazil | Asian | Human | Partial | Sanger dideoxy sequencing |
| MH379781 | 2016 | Brazil | Asian | Human | Partial | Sanger dideoxy sequencing |
| MH379782 | 2016 | Brazil | Asian | Human | Partial | Sanger dideoxy sequencing |
| KU312314 | 2015 | Suriname | Asian | Human | Partial | Sanger dideoxy sequencing |
| MH379783 | 2016 | Brazil | Asian | Human | Partial | Sanger dideoxy sequencing |
| MH379784 | 2016 | Brazil | Asian | Human | Partial | Sanger dideoxy sequencing |
| MK451708 | 2018 | Brazil | East African | Animal | Partial | Sanger dideoxy sequencing |
| MH430250 | 2016 | Viet Nam | Asian | Human | Partial | Sanger dideoxy sequencing |
| MH430251 | 2016 | Viet Nam | Asian | Human | Partial | Sanger dideoxy sequencing |
| MH430252 | 2016 | Viet Nam | Asian | Human | Partial | Sanger dideoxy sequencing |
| MH430253 | 2016 | Viet Nam | Asian | Human | Partial | Sanger dideoxy sequencing |
| KU312315 | 2015 | Suriname | Asian | Human | Partial | Sanger dideoxy sequencing |
| KU321639 | 2015 | Brazil | Asian | Human | Complete | IonTorrent |
| MF988734 | 2017 | Singapore | Asian | Human | Complete | Sanger dideoxy sequencing |
| MF988743 | 2016 | USA | Asian | Mosquito | Complete |  |
| MF996804 | 2017 | Thailand | Asian | Human | Complete | IonTorrent |
| KU365777 | 2015 | Brazil | Asian | Human | Complete |  |
| KY415986 | 2014 | Haiti | Asian | Human | Complete | Sanger dideoxy sequencing |
| KY415987 | 2014 | Haiti | Asian | Human | Complete | Sanger dideoxy sequencing |
| KY415988 | 2014 | Haiti | Asian | Human | Complete | Sanger dideoxy sequencing |
| KY415989 | 2014 | Haiti | Asian | Human | Complete | Sanger dideoxy sequencing |
| KY415990 | 2014 | Haiti | Asian | Human | Complete | Sanger dideoxy sequencing |
| KY415991 | 2014 | Haiti | Asian | Human | Complete | Sanger dideoxy sequencing |
| KU365778 | 2015 | Brazil | Asian | Human | Complete |  |
| KU365780 | 2015 | Brazil | Asian | Human | Complete |  |
| KU365779 | 2015 | Brazil | Asian | Human | Complete |  |
| KU497555 | 2015 | Brazil | Asian | Human | Complete | Illumina |
| KU501216 | 2015 | Guatemala | Asian | Human | Complete | IonTorrent |
| MK560178 | 2017 | Cuba | Asian | Human | Partial |  |
| MK560179 | 2018 | Cuba | Asian | Human | Partial |  |
| MK566202 | 2015 | Brazil | Asian | Laboratory | Complete | Sanger dideoxy sequencing |
| KY441401 | 2016 | Brazil | Asian | Human | Complete | Sanger dideoxy sequencing |
| KU501215 | 2015 | Puerto Rico | Asian | Human | Complete | IonTorrent |
| KY441402 | 2016 | Brazil | Asian | Human | Complete | Sanger dideoxy sequencing |
| KY441403 | 2016 | Brazil | Asian | Human | Complete | Sanger dideoxy sequencing |
| KU501217 | 2015 | Guatemala | Asian | Human | Complete | IonTorrent |
| MG012226 | 2015 | Colombia | Asian | Human | Partial | Sanger dideoxy sequencing |
| MG012227 | 2015 | Colombia | Asian | Human | Partial | Sanger dideoxy sequencing |
| MG012228 | 2016 | Colombia | Asian | Human | Partial | Sanger dideoxy sequencing |
| MG012229 | 2016 | Colombia | Asian | Human | Partial | Sanger dideoxy sequencing |
| MG012230 | 2016 | Colombia | Asian | Human | Partial | Sanger dideoxy sequencing |
| KU509998 | 2014 | Haiti | Asian | Human | Complete | Sanger dideoxy sequencing |
| KY465607 | 2016 | Ecuador | Asian | Mosquito | Partial | Sanger dideoxy sequencing |
| MK637518 | 2017 | Argentina | Asian | Human | Partial | Sanger dideoxy sequencing |
| MK637519 | 2016 | Argentina | Asian | Human | Partial | Sanger dideoxy sequencing |
| LR792671 | 2016 | Guadeloupe | Asian | Human | Partial |  |
| KU527068 | 2015 | Brazil | Asian | Human | Complete |  |
| KY553111 | 2016 | South Korea | Asian | Human | Complete | Illumina |
| MG216928 | 2015 | Fiji | Asian | Human | Partial | Sanger dideoxy sequencing |
| KY558989 | 2015 | Brazil | Asian | Human | Partial | MinIon R9.x, Oxford Nanopore Technologies |
| KY558990 | 2016 | Brazil | Asian | Human | Partial | MinIon R9.x, Oxford Nanopore Technologies |
| KY558991 | 2016 | Brazil | Asian | Human | Partial | MinIon R9.x, Oxford Nanopore Technologies |
| KY558992 | 2016 | Brazil | Asian | Human | Partial | MinIon R9.x, Oxford Nanopore Technologies |
| KY558993 | 2016 | Brazil | Asian | Human | Partial | MinIon R9.x, Oxford Nanopore Technologies |
| KY558994 | 2016 | Brazil | Asian | Human | Partial | MinIon R9.x, Oxford Nanopore Technologies |
| KY558995 | 2015 | Brazil | Asian | Human | Partial | MinIon R9.x, Oxford Nanopore Technologies |
| KY558996 | 2015 | Brazil | Asian | Human | Partial | MinIon R9.x, Oxford Nanopore Technologies |
| KY558997 | 2015 | Brazil | Asian | Human | Partial | MinIon R9.x, Oxford Nanopore Technologies |
| KY558998 | 2015 | Brazil | Asian | Human | Partial | MinIon R9.x, Oxford Nanopore Technologies |
| MG216929 | 2015 | Fiji | Asian | Human | Partial | Sanger dideoxy sequencing |
| KY558999 | 2016 | Brazil | Asian | Human | Complete | MinIon R9.x, Oxford Nanopore Technologies |
| KY559001 | 2015 | Brazil | Asian | Human | Complete | MinIon R9.x, Oxford Nanopore Technologies |
| KY559002 | 2015 | Brazil | Asian | Human | Partial | MinIon R9.x, Oxford Nanopore Technologies |
| KY559003 | 2015 | Brazil | Asian | Human | Complete | MinIon R9.x, Oxford Nanopore Technologies |
| KY559004 | 2016 | Brazil | Asian | Human | Complete | MinIon R9.x, Oxford Nanopore Technologies |
| KY559005 | 2016 | Brazil | Asian | Human | Complete | MinIon R9.x, Oxford Nanopore Technologies |
| KY559006 | 2016 | Brazil | Asian | Human | Complete | MinIon R9.x, Oxford Nanopore Technologies |
| KY559007 | 2016 | Brazil | Asian | Human | Complete | MinIon R9.x, Oxford Nanopore Technologies |
| KY559008 | 2016 | Brazil | Asian | Human | Partial | MinIon R9.x, Oxford Nanopore Technologies |
| MG216930 | 2016 | Fiji | Asian | Human | Partial | Sanger dideoxy sequencing |
| KY559009 | 2016 | Brazil | Asian | Human | Complete | MinIon R9.x, Oxford Nanopore Technologies |
| KY559010 | 2016 | Brazil | Asian | Human | Complete | MinIon R9.x, Oxford Nanopore Technologies |
| KY559011 | 2016 | Brazil | Asian | Human | Complete | MinIon R9.x, Oxford Nanopore Technologies |
| KY559012 | 2016 | Brazil | Asian | Human | Complete | MinIon R9.x, Oxford Nanopore Technologies |
| KY559013 | 2016 | Brazil | Asian | Human | Complete | MinIon R9.x, Oxford Nanopore Technologies |
| KY559014 | 2016 | Brazil | Asian | Human | Complete | MinIon R9.x, Oxford Nanopore Technologies |
| KY559015 | 2016 | Brazil | Asian | Human | Complete | MinIon R9.x, Oxford Nanopore Technologies |
| KY559016 | 2016 | Brazil | Asian | Human | Partial | MinIon R9.x, Oxford Nanopore Technologies |
| KY559017 | 2016 | Brazil | Asian | Human | Complete | MinIon R9.x, Oxford Nanopore Technologies |
| KY559018 | 2016 | Brazil | Asian | Human | Complete | MinIon R9.x, Oxford Nanopore Technologies |
| MG216931 | 2016 | Fiji | Asian | Human | Partial | Sanger dideoxy sequencing |
| KY559019 | 2016 | Brazil | Asian | Human | Complete | MinIon R9.x, Oxford Nanopore Technologies |
| KY559020 | 2016 | Brazil | Asian | Human | Partial | MinIon R9.x, Oxford Nanopore Technologies |
| KY559021 | 2016 | Brazil | Asian | Human | Complete | MinIon R9.x, Oxford Nanopore Technologies |
| KY559022 | 2016 | Brazil | Asian | Human | Partial | MinIon R9.x, Oxford Nanopore Technologies |
| KY559023 | 2016 | Brazil | Asian | Human | Complete | MinIon R9.x, Oxford Nanopore Technologies |
| KY559024 | 2016 | Brazil | Asian | Human | Complete | MinIon R9.x, Oxford Nanopore Technologies |
| KY559025 | 2016 | Brazil | Asian | Human | Partial | MinIon R9.x, Oxford Nanopore Technologies |
| KY559026 | 2016 | Brazil | Asian | Human | Partial | MinIon R9.x, Oxford Nanopore Technologies |
| KY559027 | 2016 | Brazil | Asian | Human | Complete | MinIon R9.x, Oxford Nanopore Technologies |
| KY559028 | 2016 | Brazil | Asian | Human | Partial | MinIon R9.x, Oxford Nanopore Technologies |
| MG216932 | 2016 | Fiji | Asian | Human | Partial | Sanger dideoxy sequencing |
| KY559029 | 2016 | Brazil | Asian | Human | Partial | MinIon R9.x, Oxford Nanopore Technologies |
| KY559030 | 2016 | Brazil | Asian | Human | Partial | MinIon R9.x, Oxford Nanopore Technologies |
| KY559031 | 2016 | Brazil | Asian | Human | Complete | MinIon R9.x, Oxford Nanopore Technologies |
| KY559032 | 2016 | Brazil | Asian | Human | Complete | MinIon R9.x, Oxford Nanopore Technologies |
| KY561343 | 2016 | Brazil | Asian | Human | Partial | Illumina |
| KY561344 | 2016 | Brazil | Asian | Human | Partial | Illumina |
| KY561345 | 2016 | Brazil | Asian | Human | Partial | Illumina |
| MK696546 | 2018 | China | Asian | Mosquito | Partial |  |
| MK696547 | 2018 | China | Asian | Mosquito | Partial |  |
| MK696548 | 2018 | China | Asian | Mosquito | Partial |  |
| MG266396 | 2016 | Hungary | Asian | Human | Partial | Sanger dideoxy sequencing |
| MK696549 | 2018 | China | Asian | Mosquito | Partial |  |
| MK696550 | 2018 | China | Asian | Mosquito | Partial |  |
| MK696551 | 2018 | China | Asian | Mosquito | Complete |  |
| MK713748 | 2015 | Puerto Rico | Asian | Human | Complete | Illumina |
| MK713750 | 2016 | Honduras | Asian | Human | Complete | Ion Torrent;Illumina |
| MH513598 | 2015 | Brazil | Asian | Human | Complete |  |
| MH513599 | 2015 | Brazil | Asian | Human | Complete |  |
| MH513600 | 2015 | Brazil | Asian | Human | Complete |  |
| MH544701 | 2016 | Colombia | Asian | Animal | Complete | Illumina; MinION |
| KU556802 | 2015 | Mexico | Asian | Human | Partial | Sanger dideoxy sequencing |
| MT078742 | 2017 | Brazil | Asian | Human | Partial | Illumina |
| MT084112 | 2018 | Nigeria | East African | Human | Partial | Sanger dideoxy sequencing |
| KY576904 | 1989 | Central African Republic | West African | Mosquito | Partial | Sanger dideoxy sequencing |
| KU740184 | 2016 | China | Asian | Human | Complete | IonTorrent |
| KU744693 | 2016 | China | Asian | Human | Complete | IonTorrent |
| MK829152 | 2017 | Angola | Asian | Human | Partial |  |
| MK829153 | 2017 | Angola | Asian | Human | Partial |  |
| MK829154 | 2017 | Portugal | Asian | Human | Complete |  |
| MG279550 | 2016 | Brazil | Asian | Mosquito | Partial | Sanger dideoxy sequencing |
| MK852163 | 2018 | India | Asian | Mosquito | Partial | Sanger dideoxy sequencing |
| KT200609 | 2015 | Brazil | Asian | Human | Partial | Sanger dideoxy sequencing |
| MF352141 | 2015 | Brazil | Asian | Human | Complete | Illumina |
| MT309004 | 2018 | Mexico | Asian | Mosquito | Partial | Sanger dideoxy sequencing |
| MT309005 | 2018 | Mexico | Asian | Mosquito | Partial | Sanger dideoxy sequencing |
| MT309006 | 2018 | Mexico | Asian | Mosquito | Partial | Sanger dideoxy sequencing |
| MT309007 | 2019 | Mexico | Asian | Mosquito | Partial | Sanger dideoxy sequencing |
| MT309008 | 2018 | Mexico | Asian | Mosquito | Partial | Sanger dideoxy sequencing |
| MF384325 | 2016 | Haiti | Asian | Mosquito | Complete | Sanger dideoxy sequencing |
| KY606271 | 2016 | Mexico | Asian | Human | Partial | Illumina |
| MG287146 | 2017 | India | Asian | Human | Partial | Sanger dideoxy sequencing |
| KY606272 | 2016 | Mexico | Asian | Human | Complete | Illumina |
| KY606273 | 2016 | Mexico | Asian | Human | Complete | Illumina |
| KY606274 | 2016 | Mexico | Asian | Human | Complete | Illumina |
| MF434516 | 2016 | Nicaragua | Asian | Human | Complete | Illumina |
| MF434517 | 2016 | Nicaragua | Asian | Human | Complete | Illumina |
| MF434518 | 2016 | Nicaragua | Asian | Human | Complete | Illumina |
| MF434519 | 2016 | Nicaragua | Asian | Human | Partial | Illumina |
| MF434520 | 2016 | Nicaragua | Asian | Human | Complete | Illumina |
| MF434521 | 2016 | Nicaragua | Asian | Human | Complete | Illumina |
| MF434522 | 2016 | Nicaragua | Asian | Human | Complete | Illumina |
| MG366906 | 2006 | Thailand | Asian | Human | Partial | Sanger dideoxy sequencing |
| MF438286 | 2017 | Cuba | Asian | Human | Complete | Sanger dideoxy sequencing |
| MF510857 | 1984 | Senegal | West African | Mosquito | Complete | Illumina |
| MF574552 | 2015 | Colombia | Asian | Human | Complete | Ion Torrent;Illumina |
| MF574553 | 2015 | Colombia | Asian | Human | Complete | Illumina |
| MF574554 | 2015 | Colombia | Asian | Human | Complete | Illumina |
| MF574555 | 2015 | Colombia | Asian | Human | Complete | Ion Torrent;Illumina |
| MF574556 | 2015 | Colombia | Asian | Human | Complete | Ion Torrent;Illumina |
| MF574557 | 2015 | Colombia | Asian | Human | Complete | Ion Torrent;Illumina |
| MF574558 | 2015 | Colombia | Asian | Human | Complete | Ion Torrent;Illumina |
| MF574559 | 2015 | Colombia | Asian | Human | Complete | Illumina |
| MG366907 | 2015 | Thailand | Asian | Human | Partial | Sanger dideoxy sequencing |
| MF574560 | 2015 | Colombia | Asian | Human | Complete | Illumina |
| MF574561 | 2015 | Colombia | Asian | Human | Complete | Ion Torrent;Illumina |
| MF574562 | 2015 | Colombia | Asian | Human | Complete | Ion Torrent;Illumina |
| MF574563 | 2015 | Colombia | Asian | Human | Complete | Ion Torrent;Illumina |
| MF574564 | 2015 | Colombia | Asian | Human | Complete | Ion Torrent;Illumina |
| MF574565 | 2015 | Colombia | Asian | Human | Complete | Illumina |
| MF574566 | 2015 | Colombia | Asian | Human | Complete | Illumina |
| MF574567 | 2015 | Colombia | Asian | Human | Complete | Illumina |
| MF574568 | 2015 | Colombia | Asian | Human | Complete | Ion Torrent;Illumina |
| MF574569 | 2015 | Colombia | Asian | Human | Complete | Ion Torrent;Illumina |
| KU646827 | 2015 | Colombia | Asian | Human | Partial | Sanger dideoxy sequencing |
| MF574570 | 2015 | Colombia | Asian | Human | Complete | Illumina |
| MF574571 | 2015 | Colombia | Asian | Human | Complete | Ion Torrent;Illumina |
| MF574572 | 2015 | Colombia | Asian | Human | Complete | Illumina |
| MF574573 | 2015 | Colombia | Asian | Human | Complete | Ion Torrent;Illumina |
| MF574574 | 2015 | Colombia | Asian | Human | Complete | Ion Torrent;Illumina |
| MF574575 | 2015 | Colombia | Asian | Human | Complete | Illumina |
| MF574576 | 2015 | Colombia | Asian | Human | Complete | Ion Torrent;Illumina |
| MF574577 | 2015 | Colombia | Asian | Human | Complete | Ion Torrent;Illumina |
| MF574578 | 2016 | Colombia | Asian | Human | Complete | Ion Torrent;Illumina |
| MF574579 | 2016 | Colombia | Asian | Human | Complete | Illumina |
| KU647676 | 2015 | Martinique | Asian | Human | Complete | IonTorrent |
| MF574580 | 2016 | Colombia | Asian | Human | Complete | Ion Torrent;Illumina |
| MF574581 | 2016 | Colombia | Asian | Human | Complete | Ion Torrent;Illumina |
| MF574582 | 2016 | Colombia | Asian | Human | Complete | Ion Torrent;Illumina |
| MF574583 | 2016 | Colombia | Asian | Human | Complete | Illumina |
| MF574584 | 2016 | Colombia | Asian | Human | Complete | Ion Torrent;Illumina |
| MF574585 | 2016 | Colombia | Asian | Human | Complete | Illumina |
| MF574586 | 2016 | Colombia | Asian | Human | Complete | Ion Torrent;Illumina |
| MK972825 | 2019 | Brazil | Asian | Mosquito | Partial | Sanger dideoxy sequencing |
| MF574587 | 2016 | Colombia | Asian | Human | Complete | Ion Torrent;Illumina |
| MF574588 | 2016 | Colombia | Asian | Human | Complete | Ion Torrent;Illumina |
| KU646828 | 2015 | Colombia | Asian | Human | Partial | Sanger dideoxy sequencing |
| MK972826 | 2018 | Brazil | East African | Mosquito | Partial | Sanger dideoxy sequencing |
| MF593625 | 2016 | China | Asian | Human | Complete |  |
| MN025403 | 2018 | Guinea | West African | Human | Complete | Illumina; Sanger dideoxy sequencing |
| MT439638 | 2016 | Brazil | Asian | Human | Partial | MinIon - Oxford Nanopore |
| MT439639 | 2016 | Brazil | Asian | Human | Partial | MinIon - Oxford Nanopore |
| MT439640 | 2016 | Brazil | Asian | Human | Partial | MinIon - Oxford Nanopore |
| MT439641 | 2016 | Brazil | Asian | Human | Complete | MinIon - Oxford Nanopore |
| MT439642 | 2016 | Brazil | Asian | Human | Complete | MinIon - Oxford Nanopore |
| MT439643 | 2016 | Brazil | Asian | Human | Complete | MinIon - Oxford Nanopore |
| MT439644 | 2016 | Brazil | Asian | Human | Complete | MinIon - Oxford Nanopore |

**Table S1** Summary of Zika virus sequences retrieved from GenBank (https://www.ncbi.nlm.nih.gov/nuccore) from 1947 to October 2022. Metadata includes accession number, country and year of isolation, lineage, host, and genome type (complete or partial).
