# Supplementary material for: ZIKV Phylogenetic Characterization Reveals Evolutionary Diversity, Regional Dissemination, and Emergence of African Lineages in Brazil
Source: J Basic Microbiol. 2025 Oct 28;66(1):e70122. doi: 10.1002/jobm.70122 (PMC12706136; doi:10.1002/jobm.70122)

| **Sample** | **Sample Type** |
| --- | --- |
| Sample 0 | Saliva |
| Sample 1 | Urine |
| Sample 2 | Saliva |
| Sample 3 | Urine |
| Sample 4 | Saliva |
| Sample 5 | Urine |
| Sample 6 | Saliva |
| Sample 7 | Urine |
| Sample 8 | Saliva |
| Sample 9 | Saliva |
| Sample 10 | Saliva |
| Sample 35 | Serum |
| Sample 38 | Serum |
| Sample 39 | Serum |
| Sample 40 | Serum |
| Sample 43 | Serum |
| Sample 44 | Saliva |
| Sample 45 | Urine |
| Sample 51 | Saliva |
| Sample 54 | Saliva |
| OIV1 | Eggs |
| LN1 | Larvae |
| MC2 | Mosquitoes |
| MC4 | Mosquitoes |
| FARG 3 | Mosquitoes |
| FARG 4 | Mosquitoes |
| FARG 6 | Mosquitoes |
| FARG 7 | Mosquitoes |
| FARG 10 | Mosquitoes |

**Table S2** Identification of the 29 ZIKV samples analyzed in this study based on the C-prM region. Clinical samples were obtained from saliva, urine, and serum of infected individuals, while entomological samples were derived from mosquito pools (eggs, larvae, and adult mosquitoes).

**Figure S1** Illustrative image of the alignment of the C-prM genomic region from the 826 sequences used in the polymorphism analysis of ZIKV lineages


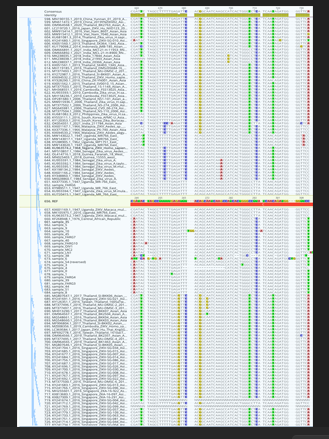

Supplement: Supplementary file 3 — Table S2: Identification of the 29 ZIKV samples analyzed in this study based on the C‐prM region. Clinical samples were obtained from saliva, urine, and serum of infected individuals, while entomological samples were derived from mosquito pools (eggs, larvae, and adult mosquitoes). [file JOBM-66-e70122-s003.docx]
